# Synthesis of Benzofused *O*- and *N*-Heterocycles through Cascade Carbopalladation/Cross-Alkylation of Alkynes Involving the C–C Cleavage of Cyclobutanols

**DOI:** 10.1021/acs.organomet.2c00015

**Published:** 2022-03-03

**Authors:** Marta Pérez-Gómez, Piedad Herrera-Ramírez, Delia Bautista, Isabel Saura-Llamas, José-Antonio García-López

**Affiliations:** † Grupo de Química Organometálica, Departamento de Química Inorgánica, Facultad de Química, Universidad de Murcia, E−30100 Murcia, Spain; ‡ ACTI, Universidad de Murcia, E−30100 Murcia, Spain

## Abstract

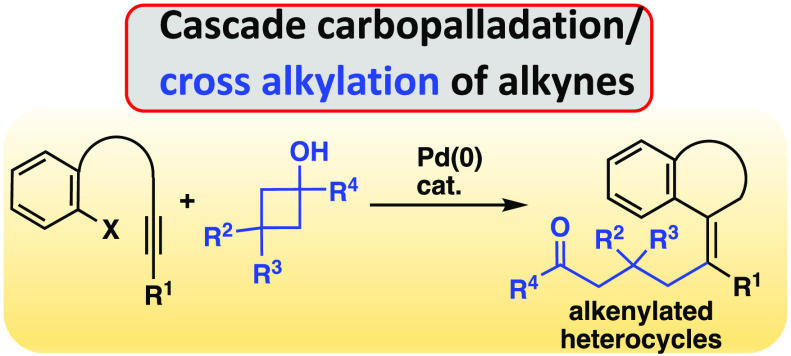

We report a Pd-catalyzed route to heterocycles bearing a tetrasubstituted alkene fragment. Our approach merges the intramolecular carbopalladation of tethered alkynes with an alkylation step produced by the C–C cleavage of cyclobutanol derivatives. An alkenyl-Pd(II) intermediate has been isolated and characterized by X-ray diffraction studies. Interestingly, the nature of the tethering alkynyl chain influences the *E*/*Z* stereochemistry of the alkenyl fragment in the functionalized heterocycles.

## Introduction

**Scheme 1:**
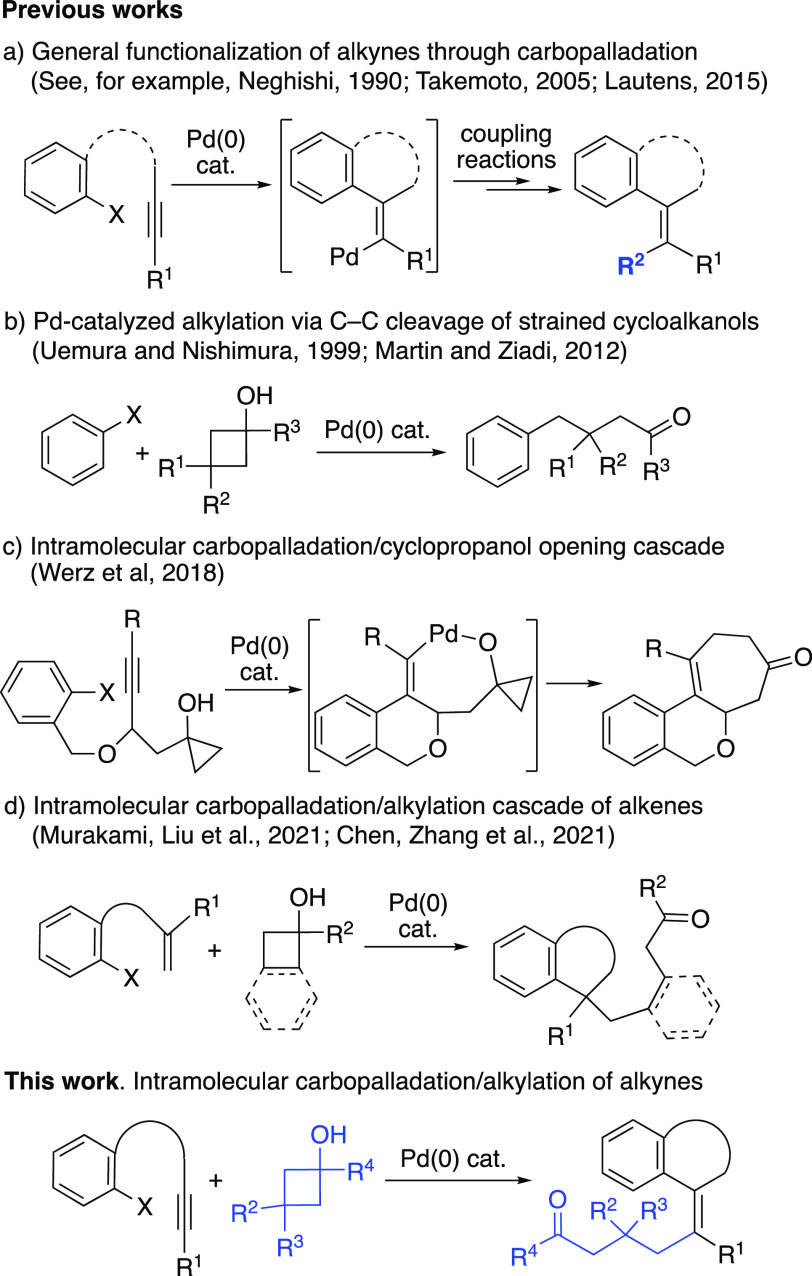
Merger of Carbopalladation of Alkynes and C–C Cleavage of Cycloalkanols

The development of Pd-catalyzed cascade reactions based on the carbopalladation of alkynes has become a direct entry to the synthesis of substituted alkenes.^1−9^ Such reactions have been performed in either intra- or intermolecular fashion, with the resulting alkenyl-Pd intermediate being coupled afterward with different species, such as boronic acids,^10−12^ organotin reagents,^13−18^ and *C*-,^19^*N*-,^20,21^ and *O*-nucleophiles,^22^ among many others (**a**, Scheme 1).^23−28^

Parallel studies have demonstrated the ability of Pd to perform the opening of strained cycloalkanols through β-carbon elimination (**b**, Scheme 1).^29,30^ This process leads to a σ-alkyl-Pd(II) intermediate, which can evolve in different manners, depending on the substitution pattern of the cycloalkanol.^31−37^ For instance, they can participate in further intramolecular steps, or be cross-coupled with aryl-,^38−42^ alkenyl-,^43,44^ and alkynylhalides,^45^ or propargylcarbonates,^46^ among others.^29,47,48^ Therefore, cyclopropyl- or cyclobutyl alcohols can behave as alkylating reagents under the appropriate conditions.

The merging of both aspects of palladium chemistry (carbopalladation/alkylation via opening of cycloalkanols) has rarely been reported in the literature. Werz et al. disclosed an interesting cascade reaction relying on the formal *anti*-carbopalladation of an internal alkyne, evolving through further intramolecular trapping of the alkenyl-Pd(II) intermediate by a tethered cyclopropanol moiety (**c**, Scheme 1).^49^ Very recently, Murakami, Chen, and co-workers reported the synthesis of 2,3-dihydrobenzofurans through the use of alkenyl-tethered aryliodides and benzocyclobutanols (**d**, Scheme 1).^50,51^

With these precedents in mind, and given our interest in the topics of Pd chemistry and the processes related to C–C cleavage,^52−57^ we aimed to extend the applicability of these types of cascades to the synthesis of heterocycles bearing an alkylated olefine moiety (Scheme 1).

## Results and Discussion

We studied the feasibility to perform the envisioned carbopalladation/alkylation cascade reaction employing the 2-bromoarylether **1a** and the cyclobutanol derivative **2a** (Table 1). Initial screening of experimental conditions revealed the formation of some amounts of the byproduct **4a**, likely arising from the protodepalladation of the plausible alkenyl-Pd(II) intermediate generated upon the carbopalladation of the internal alkyne moiety. The use of 10 mol% of [Pd(dba)_2_] along with 20 mol% of PPh_3_ showed good selectivity to give the desired compound **3a** in THF or toluene as solvents (entries 3 and 4, Table 1). Replacing PPh_3_ by other ligands such as JohnPhos, PCy_3_, or Xantphos did not improve the yields of **3a** (entries 5–7, Table 1). The increase of the amount of Cs_2_CO_3_ in the reaction mixture could not suppress the protodepalladation process leading to the byproduct **4a**, and other organic bases like NEt_3_ precluded the formation of **3a**. We tested Pd sources like Pd(OAc)_2_, [PdCl_2_(PPh_3_)_2_], and [Pd(PPh_3_)_4_]. While the first two were not effective for this transformation, [Pd(PPh_3_)_4_] showed a comparable activity to [Pd(dba)_2_], reaching a 70% yield of the desired product.

**Table 1:**
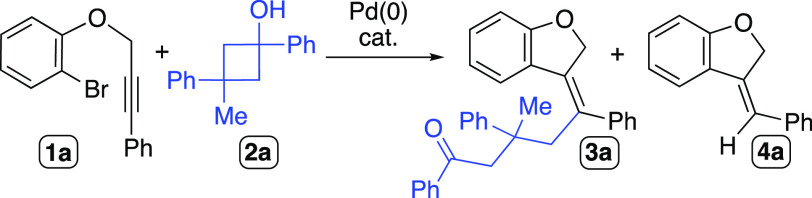
Optimization of the Carbopalladation/Alkylation Cascade^a^

entry^a^	Pd source (10 mol %)	ligand (20 mol %)	solvent	yield **3a**^b^
1	[Pd(dba)_2_]	PPh_3_	1,2-DCE	traces
2	[Pd(dba)_2_]	PPh_3_	1,4- dioxane	traces
3	[Pd(dba)_2_]	PPh_3_	THF	62
4	[Pd(dba)_2_]	PPh_3_	toluene	68
5	[Pd(dba)_2_]	JohnPhos	toluene	–
6	[Pd(dba)_2_]	PCy_3_	toluene	60
7	[Pd(dba)_2_]	Xantphos	toluene	32
8	[Pd(OAc)_2_]	PPh_3_	toluene	traces
9	[PdCl_2_(PPh_3_)_2_]	–	toluene	traces
10	[Pd(PPh_3_)_4_]	–	toluene	70 (67)^c^

a The reactions were carried out using 0.14 mmol of 1-bromo-2-((3-phenylprop-2-yn-1-yl)oxy)benzene (**1a**), 1.2 equiv of 3-methyl,-1,3-diphenylcyclobutan-1-ol (**2a**), and 1.2 equiv of Cs_2_CO_3_ in 4 mL of dry solvent, under nitrogen atmosphere at 100 °C, in a Carius tube for 16 h.

b NMR yields using trimethylbenzene-1,3,5-tricarboxylate as standard.

c Isolated yield.

With the optimized conditions in hand, we proceeded to study the scope and limitations of the reaction. Several aspects were assessed: the presence of electron-donating/withdrawing groups in the haloaryl moiety, the nature and length of the chain tethering the internal alkyne, and the use of different substituted cyclobutanols.

**Scheme 2:**
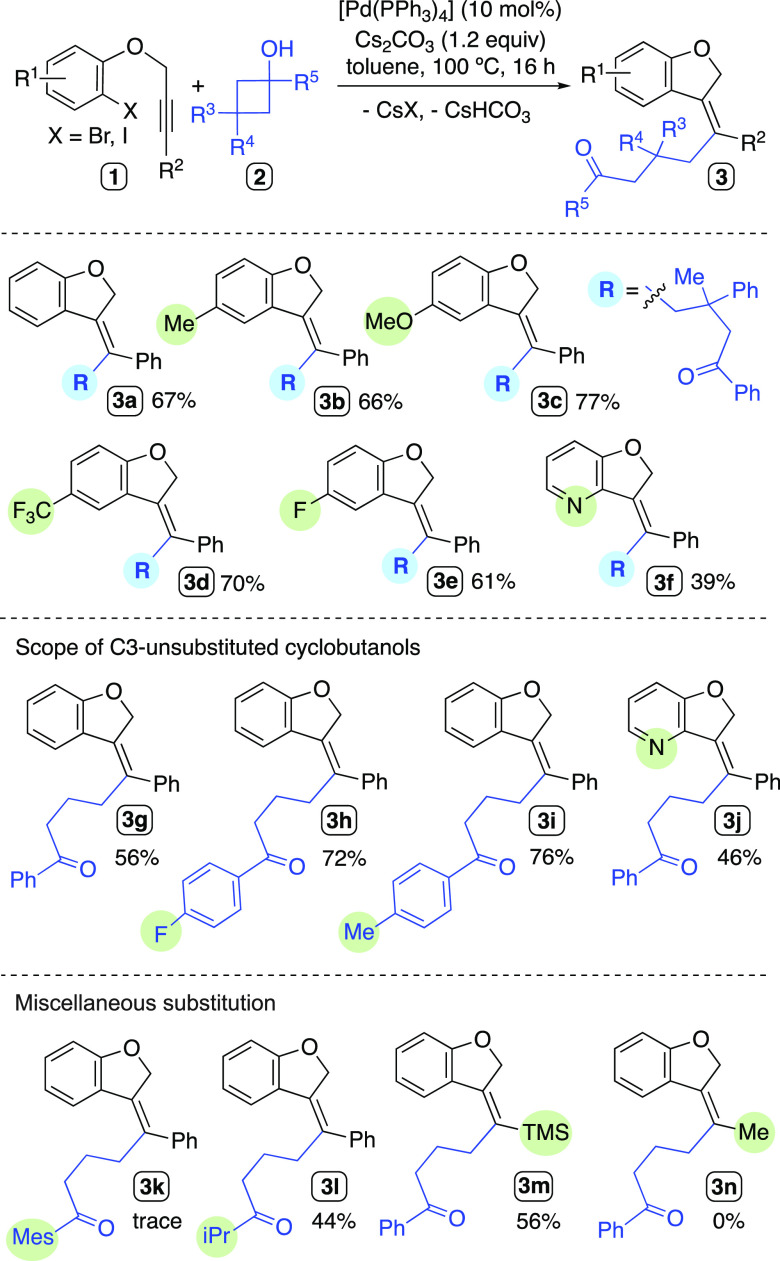
Scope of the Carbopalladation/Alkylation Cascade for the Synthesis of Dihydrobenzofurane Derivatives

The reactions of haloaryl ethers bearing methyl, methoxy, fluoro, or trifluoromethyl substituents with the 3,3-substituted cyclobutanol **2a** afforded good yields of the expected dihydrobenzofuran derivatives **3b**–**3e** (Scheme 2). The pyridine derivative **1g** gave rise to the heterocycle **3f**, albeit in moderate yield, perhaps due to competing coordination of the pyridine moiety to Pd(II). C3-unsubstituted cyclobutanol derivatives **2** were also productive in the cascade reaction, giving the functionalized dihydrobenzofuran derivatives **3g**–**j** in comparable yields to those obtained with **2a** (Scheme 2); therefore, the possible byproduct formation arising from β-H elimination processes seem to be overridden. The cyclobutanol derivative bearing a mesityl group in α-position led to mixtures where the desired compound **3k** could not be identified. The compound **3l** could be isolated in 44% yield from the reaction carried out employing the tertiary cyclobutanol bearing an *i*-Pr group.

Finally, the cross-coupling reactions of **2b** and Me- or TMS-substituted alkynyl substrates were tested. We observed that among such substrates, only the silylated alkyne was competent to deliver the desired product **3m** in 56% yield (Scheme 2). Possibly, the substrate leading to **3n** could experience a β-H elimination upon the carbopalladation step to render an allenyl moiety, as described in other Pd-catalyzed reactions dealing with alkyl-substituted alkynes.^58,59^

**Scheme 3:**
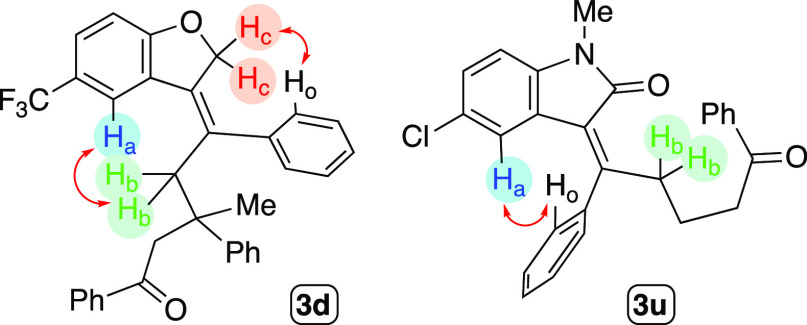
Selected NOE Contacts Observed for Dihydrobenzofurane and Oxindole Derivatives

In order to assess the stereochemistry of the exocyclic double bond present in the dihydrobenzofuran cores, a NOESY NMR experiment was carried out for compound **3d**. The NOE contacts between the methylene group CH_2c_ and the *o*-H atoms from the Ph ring, as well as the H_a_ of the heterocycle with the CH_2b_ group of the aliphatic chain, pointed out the *Z*-stereochemistry for these compounds (Scheme 3).

As a general feature of compounds **3a**–**3m**, we observed their relative sensitivity to chromatography purification in either silica gel or alumina. The decomposition of the compounds could be minored by using silica gel previously deactivated with Et_3_N, and Et_3_N/hexane/EtOAc mixtures as eluents. Solutions of these compounds in CDCl_3_ also evolved to more complex mixtures over time (see the Supporting Information). The instability of these compounds might be due to the migration of the exocyclic double bond to form benzofuran derivatives, a process that could be catalyzed by Lewis acids.^60^

**Scheme 4:**
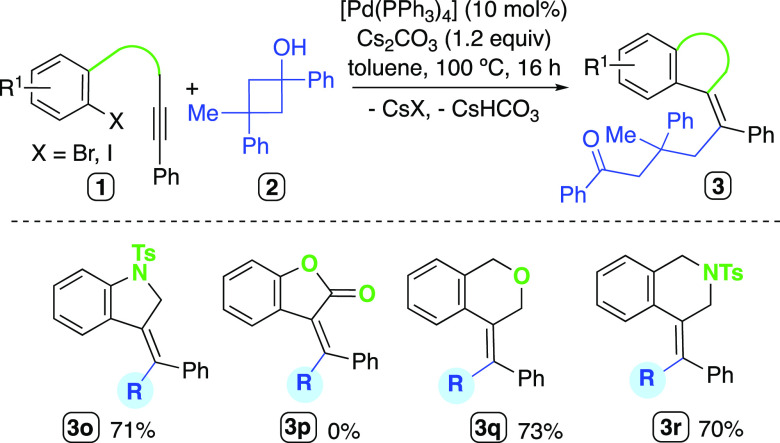
Scope of the Carbopalladation/Alkylation Cascade Varying the Nature of the Linking Chain

**Scheme 5:**
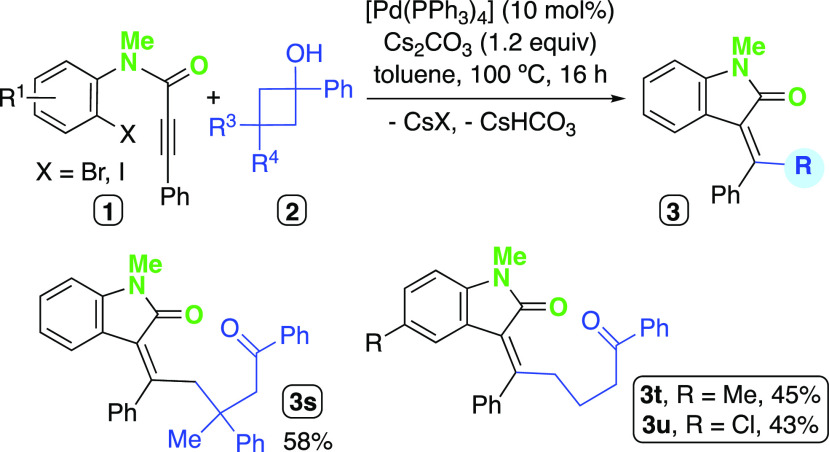
Use of Propiolamide Substrates

We examined the influence of the length and nature of the chain linking the 2-haloryl and alkyne fragments. The alkenylated indoline derivative **3o** was obtained in good yield from the corresponding amine precursor (Scheme 4). Nevertheless, no desired product **3p** was produced from the related ester starting material. Substrates with one extra carbon atom in the chain reacted smoothly under the optimized conditions to produce the six-membered heterocycles **3q** and **3r**. The ^1^H NMR of the crude reaction mixture arising from *N*-(2-bromo-phenyl)-*N*-methyl-3-phenylpropiolamide showed the formation of the corresponding coupling product **3s** as the main component, which could be isolated in 58% yield (Scheme 5). Similarly, the oxindole derivatives **3t** and **3u** could be isolated in moderate yields from the reactions of the corresponding propiolamides and the C3-unsubstituted cyclobutanol **2b**. The ^1^H NMR spectra of compounds **3s**–**u** showed an aromatic signal belonging to the oxindole core at a relatively low chemical shift (5.8–6.0 ppm). This shielding on H_a_ (compound **3u**, Scheme 3) is provoked by the phenyl ring on the exocyclic olefine moiety, as observed in related structures reported in the literature.^23,61,62^ In addition, the NOESY NMR analysis of **3u** also confirmed the *E*-stereochemistry of the exocyclic double bond. The presence of minor *Z*-stereoisomers in the reaction mixtures leading to **3s**–**u** cannot be discarded; however, we were unable to isolate such minor components of the crude mixtures and identify their nature unambiguously.

**Chart 1:**
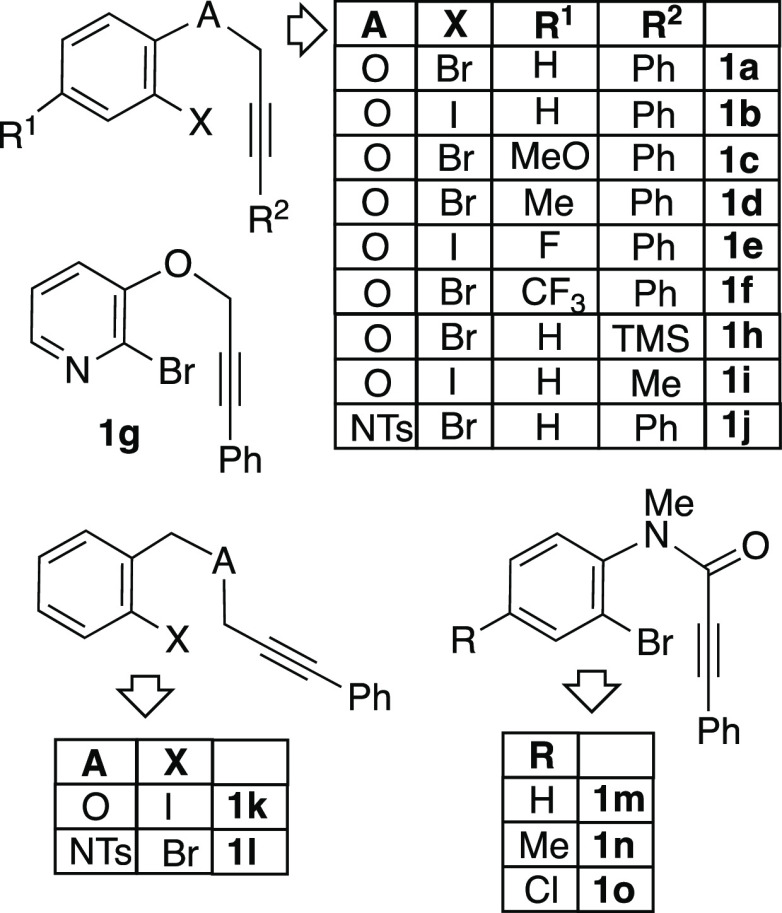
Structure and Numbering of the Staring Materials **1**

**Scheme 6:**
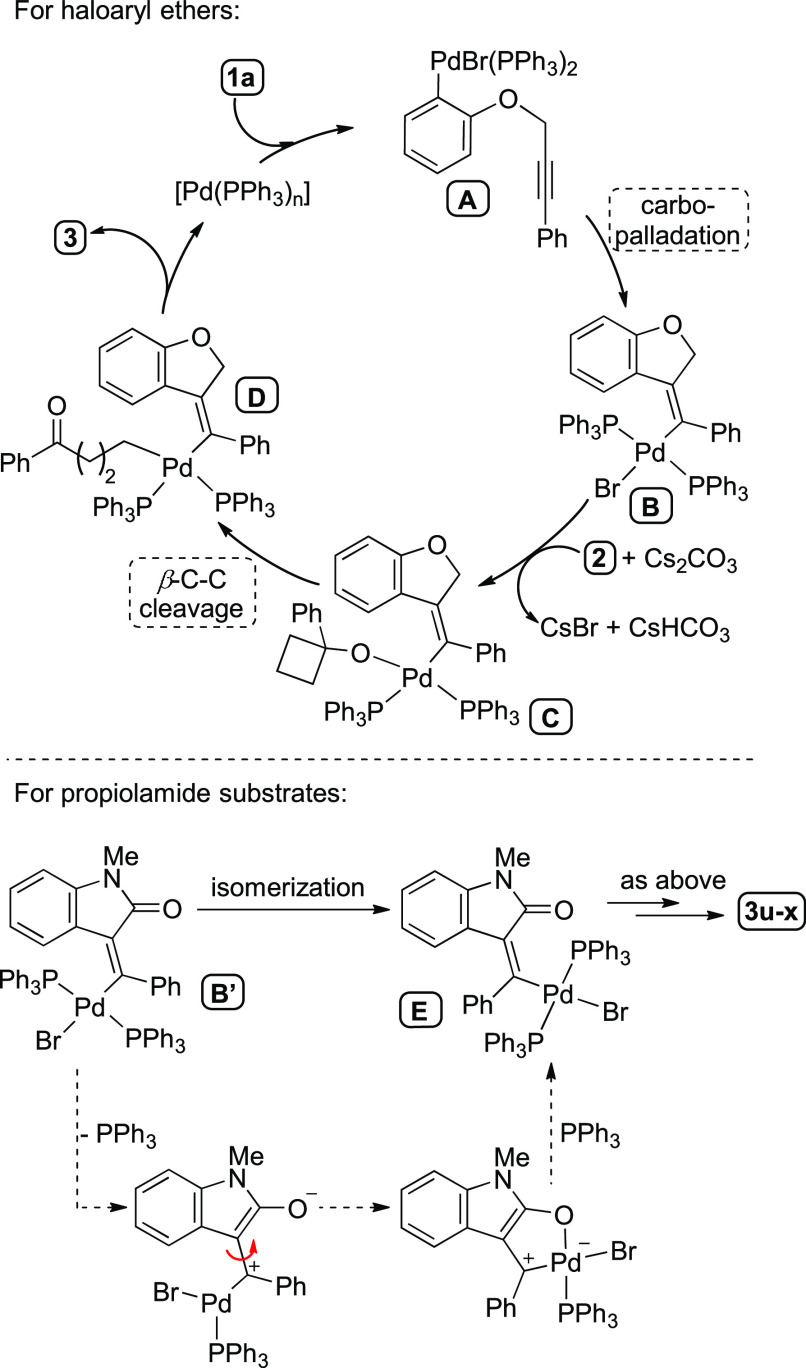
Proposed Reaction Mechanism

The plausible mechanistic pathway for this reaction is depicted in Scheme 6. The aryl-Pd species **A** would form upon oxidative addition of the C–Br bond present in the starting material **1a** to Pd(0) (Chart 1). Next, the intramolecular *syn* carbopalladation of the tethered alkyne would render the intermediate **B**. At this stage, Cs_2_CO_3_ would assist the deprotonation of the cycloalkanol, along with the removal of the halogen ligand from the coordination sphere, allowing the formation of the alkoxide complex **C**. The opening of the strained cycloalkanol through β-C cleavage would render the σ-alkyl-Pd(II) intermediate **D**, from which reductive elimination could take place to deliver the substituted olefin **3a** upon C(sp^2^)–C(sp^3^) bond formation.

The fact that propiolamide substrates afford the *E-*alkenylated oxindoles **3s**–**u** as main coupling products reveals that in those cases the alkenyl-Pd(II) intermediate, arising from the *syn* carbopalladation step, could undergo an isomerization process. There are several precedents in the literature of related Pd-catalyzed cascade reactions involving the *syn* carbopalladation of alkynes and subsequent isomerization prior to the final C–Pd bond functionalization.^14,22,25,63−67^ Generally, the isomerization of the alkenyl-Pd intermediates is driven by steric factors. Nevertheless, α-alkyl-substituted alkynyl substrates, such as **1a**, require the use of bulky phosphine ligands (Q-Phos, X-Phos, or P^*t*^Bu_3_ among others) to increase the steric hindrance around the Pd center and therefore promote the isomerization.^25,63,64^ In the case of α-acyl-substituted alkynyl substrates, such as propiolamides **1m**–**o**, the isomerization is a frequent feature in a range of different conditions, probably due to the conjugation of the alkenyl-Pd moiety and the carbonyl group, which might lower the energy barrier for the C–C rotation process (Scheme 6).^28,62,68,69^ Likely the coordination of the carbonyl moiety might facilitate such processes. Nevertheless, the opposite isomerization has been observed in related systems (that is, the steric factors seemed to predominate over the possible coordination of the carbonyl group in intermediates such as **E**).^68,69^

**Scheme 7:**
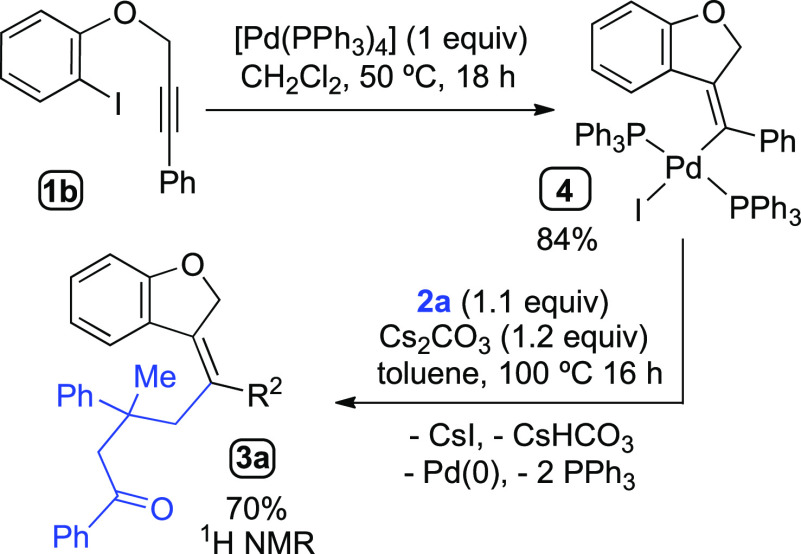
Synthesis of Intermediate **B**

We carried out the reaction of substrate **1b** with a stoichiometric amount of [Pd(PPh_3_)_4_] in CH_2_Cl_2_ at 50 °C for 18 h under N_2_ atmosphere (Scheme 7). From the reaction mixture, the vinyl-Pd(II) complex **4** (analogous to the intermediate **B**) could be isolated in 84% yield. The complex **4** was subsequently heated in toluene at 100 °C in the presence of cyclobutanol **2a** and Cs_2_CO_3_. The ^1^H NMR spectra of the crude reaction mixture confirmed the formation of the functionalized dihydrobenzofuran **3a** in 70% yield.

**Chart 2:**
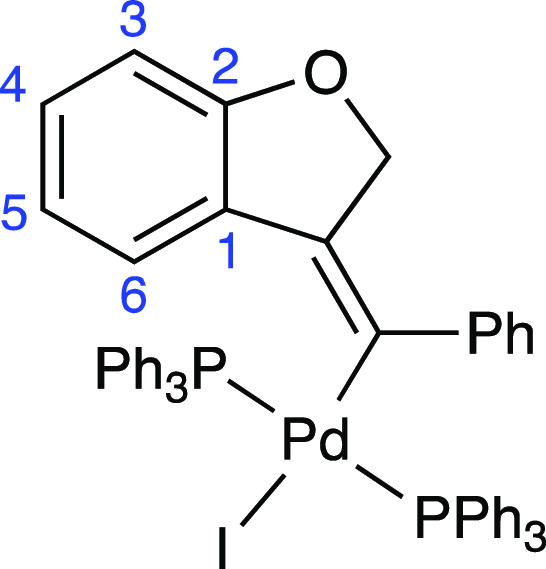
Structure and Numbering of the Intermediate Complex **4**

**Figure 1:**
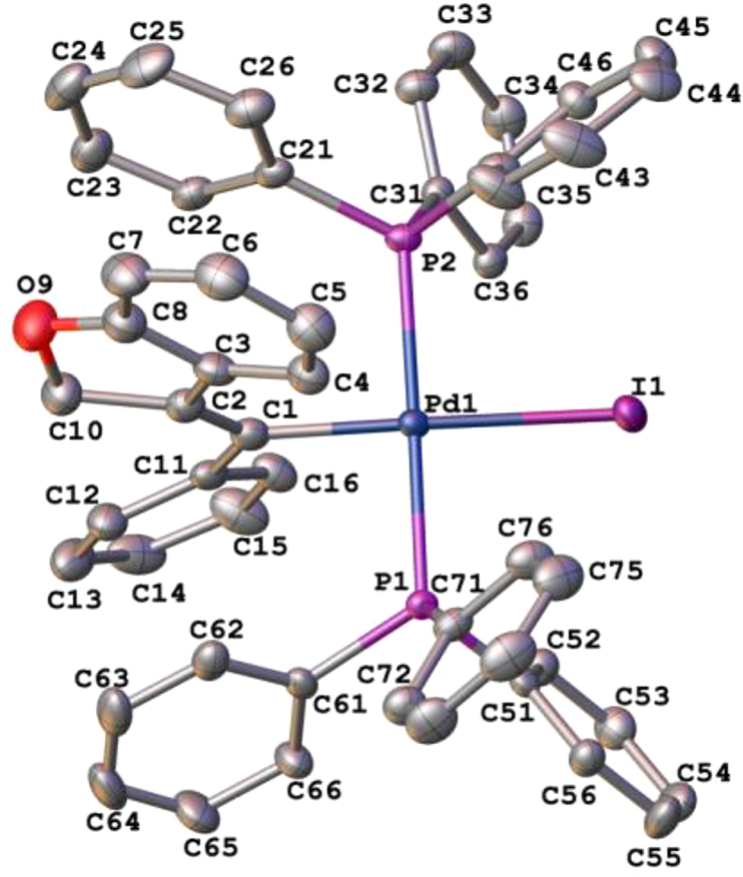
Thermal ellipsoid plot (50% probability) of complex **4** along with the labeling scheme. The hydrogen atoms have been omitted for clarity. Selected bond lengths (Å) and angles (deg): Pd(1)–I(1) = 2.6995(4), Pd(1)–P(1) = 2.3376(8), Pd(1)–P(2) = 2.3501(9), Pd(1)–C(1) = 2.051(4), C(1)–C(2) = 1.339(5), C(1)–C(11) = 1.505(5); I(1)–Pd(1)–P(1) = 90.85(2), P(1)–Pd(1)–C(1) = 89.59(10), C(1)–Pd(1)–P(2) = 89.91(10), P(2)–Pd(1)–I(1) = 90.15(2), C(2)–C(1)–Pd(1) = 123.4(3), C(2)–C(1)–C(11) = 122.9(3), C(11)–C(1)–Pd(1) = 113.7(2).

The crystal structure of complex **4** was solved by X-ray diffraction studies (Figure 1, Chart 2). The PPh_3_ ligands adopted a *trans* disposition. The palladium atom was in a slightly distorted square-planar environment, with a mean deviation of the Pd(II) coordination plane of 0.088 Å. The exocyclic double bond exhibited a *E* geometry, with the phenyl ring located *cis* to the methylene group of the dihydrobenzofuran ring. The heterocyclic nucleus formed angles of 38.1° and 77.1° with the phenyl substituent at the double bond and the Pd(II) coordination plane, respectively. This way, the phenyl ring was rotated 23.3° with respect to the exocyclic double bond plane.

## Conclusion

In summary, we have expanded the versatility of Pd cascades relying on intramolecular carbopalladation processes through its merging with the opening of strained cycloalkanols. Thus, the carbopalladation of tethered alkynes followed by an alkylation process delivers interesting *O-* and *N*-heterocyclic cores bearing a fully substituted exocyclic double bond. In addition, we observed a different behavior of haloarylether and propiolamide substrates, being the last ones prone to afford the coupling products arising from isomerization of the alkenyl-Pd(II) intermediate.

## Experimental Section

### General Remarks

Infrared spectra were recorded on a PerkinElmer spectrum 100 spectrophotometer. High-resolution ESI mass spectra were recorded on an Agilent 6220 Accurate Mass TOF LC-MS spectrometer. Melting points were determined using a Reichert apparatus and are uncorrected. Nuclear magnetic resonance (NMR) spectra were recorded on a 300, 400, or 600 MHz Bruker NMR spectrometers in CDCl_3_ at 298 K (unless stated otherwise). All chemical shift values are reported in parts per million (ppm) with coupling constant (*J*) values reported in Hz. All spectra were referenced to TMS for ^1^H NMR and the CDCl_3_ solvent peak for ^13^C{^1^H} NMR. The anhydrous solvents were purchased from commercial sources and used as received. TLC tests were run on TLC Alugram Sil G plates and visualized under UV light at 254 nm. Chromatography: Separations were carried out on silica gel. The general procedures and characterization for the substrates **1a**–**o** are included in the Supporting Information.

### Representative Procedure A for the Synthesis of the Carbopalladation/Alkylation Cascade Products **3**

A Carius tube equipped with a magnetic stirrer was charged with [Pd(PPh_3_)_4_] (16 mg, 10 mol %), Cs_2_CO_3_ (51 mg, 0.17 mmol, 1.2 equiv), 3-methyl,-1,3-diphenylcyclobutan-1-ol (40 mg, 0.17 mmol, 1.2 equiv), and the corresponding substrate **(1a)** (40 mg, 0.14 mmol). The tube was set under a nitrogen atmosphere, and dry toluene (4 mL) was added. The tube was sealed, and the reaction mixture was stirred for 16 h at 100 °C. After cooling the tube, the crude was diluted with CH_2_Cl_2_ (50 mL) and filtered through a plug of Celite. The filtrate was concentrated under a vacuum, and the crude mixture was purified by column chromatography to afford the desired cascade product **(3a)**. Compounds **3a**–**o** are sensitive to purification in silica gel chromatography; therefore, the silica gel was previously deactivated with Et_3_N. In addition, *n*-hexane containing 1% Et_3_N and EtOAc mixtures were used as eluents.

### Compound (*Z*)-5-(Benzofuran-3(2*H*)-ylidene)-3-methyl-1,3,5-triphenylpentan-1-one (**3a**)

Prepared according to the representative procedure A from 0.14 mmol of substrate **1a** and 0.17 mmol of 3-methyl-1,3-diphenylcyclobutan-1-ol (**2a**). The crude was purified by column chromatography over silica gel using 0 to 15% gradient EtOAc in *n*-hexane to afford the heterocycle **3a** as an orange oil (42 mg, 0.095 mmol, 67%). IR (cm^–1^) ν̅ 1599 (s), 1493 (s), 1445 (s), 1242 (s), 1113 (s), 1039 (s), 1024 (s), 755 (s), 691 (s). ^1^H NMR (300 MHz, CDCl_3_) δ 7.62 (dd, *J* = 7.9, 1.3 Hz, 1 H), 7.56–7.50 (m, 2 H), 7.48–7.39 (m, 2 H), 7.37–7.24 (m, 6 H), 7.22–7.07 (m, 6 H), 6.92–6.76 (m, 2 H), 5.10–4.60 (m, 2 H), 3.44–3.39 (m, 3 H), 3.10 (d, *J* = 17.2 Hz, 1 H), 1.65 (s, 3 H). ^13^C NMR (75.45 MHz, CDCl_3_) δ 197.9 (s, C_q_), 164.4 (s, C_q_), 147.5 (s, C_q_), 143.9 (s, C_q_), 137.7 (s, C_q_), 135.8 (s, C_q_), 132.5 (s, CH), 130.9 (s, C_q_), 129.8 (s, CH), 128.7 (s, CH), 128.2 (s, CH), 128.0 (s, CH), 127.7 (s, CH), 127.6 (s, CH), 126.9 (s, CH), 125.7 (s, CH), 125.6 (s, CH), 125.1 (s, C_q_), 124.1 (s, CH), 120.3 (s, CH), 110.5 (s, CH), 75.4 (s, CH_2_), 49.3 (s, CH_2_), 46.1 (s, CH_2_), 42.0 (s, C_q_), 24.2 (s, CH_3_). HRMS (+ESI) *m*/*z* calculated for C_32_H_28_NaO_2_ [M + Na]^+^ 467.1981, found 467.1986.

### Compound (*Z*)-3-Methyl-5-(5-methylbenzofuran-3(2*H*)-ylidene)-1,3,5-triphenylpentan-1-one (**3b**)

Prepared according to the representative procedure A from 0.14 mmol of substrate **1d** and 0.17 mmol of 3-methyl,-1,3-diphenylcyclobutan-1-ol (**2a**). The crude was purified by column chromatography over silica gel using 0 to 10% gradient EtOAc in *n*-hexane containing 1% Et_3_N to afford the heterocycle **3b** as a yellow oil (41 mg, 0.09 mmol, 64%). IR (cm^–1^) ν̅ 1688.4 (s), 1596.8 (s), 1492.6 (s), 1480.1 (s), 1445.4 (s), 1213.9 (s), 755.7 (s), 691.3 (s). ^1^H NMR (300 MHz, CDCl_3_) δ 7.60–7.52 (m, 2 H), 7.50–7.41 (m, 1 H), 7.39–7.37 (m, 1 H), 7.36–7.31 (m, 3 H), 7.31–7.24 (m, 3 H), 7.23–7.18 (m, 2 H), 7.18–7.12 (m, 3 H), 7.11–7.04 (m, 1 H), 6.99–6.93 (m, 1 H), 6.70 (d, *J* = 8.1 Hz, 1 H), 4.87 (br s, 2 H), 3.61–3.26 (m, 3 H), 3.09 (d, *J* = 17.2 Hz, 1 H), 2.28 (s, 3 H), 1.66 (s, 3 H). ^13^C NMR (75.45 MHz, CDCl_3_) δ 198.0 (s, C_q_), 162.6 (s, C_q_), 147.6 (s, C_q_), 144. 2(s, C_q_), 137.9 (s, C_q_), 136.2 (s, C_q_), 132.7 (s, CH), 130.6 (s, C_q_), 130.5 (s, CH), 129.5 (s, C_q_), 128.8 (s, CH), 128.4 (s, CH), 128.2 (s, CH), 127.9 (s, CH), 127.8 (s, CH), 127.0 (s, CH), 125.8 (s, CH), 125.7 (s, CH), 125.2 (s, C_q_), 124.7 (s, CH), 110.1 (s, CH), 75.7 (s, CH_2_), 49.3 (s, CH_2_), 46.6 (s, CH_2_), 42.3 (s, C_q_), 24.3 (s, CH_3_), 21.2 (s, CH_3_). HRMS (+ESI) *m*/*z* calculated for C_33_H_30_NaO_2_ [M + Na]^+^ 481.2138, found 481.2130.

### Compound (*Z*)-5-(5-Methoxybenzofuran-3(2*H*)-ylidene)-3-methyl-1,3,5-triphenylpentan-1-one (**3c**)

Prepared according to the representative procedure A from 0.14 mmol of substrate **1c** and 0.17 mmol of 3-methyl,-1,3-diphenylcyclobutan-1-ol **(2a)**. The crude was purified by column chromatography over silica gel using gradient from 0 to 20% EtOAc in *n*-hexane containing 1% Et_3_N to afford the heterocycle **3c** as a light-yellow oil (52 mg, 0.11 mmol, 78%). IR (cm^–1^) ν̅ 1681 (s), 1598 (s), 1481 (s), 1202 (s), 1021 (s), 755 (s), 691 (s). ^1^H NMR (400 MHz, CDCl_3_) δ 7.60–7.53 (m, 2 H), 7.46 (ddt, *J* = 7.8, 6.9, 1.3 Hz, 1 H), 7.36–7.31 (m, 4 H), 7.31–7.26 (m, 2 H), 7.25–7.19 (m, 2 H), 7.20–7.16 (m, 2 H), 7.16–7.11 (m, 2 H), 7.12–7.02 (m, 1 H), 6.83–6.71 (m, 2 H), 5.05–4.78 (m, 2 H), 3.77 (s, 3 H), 3.52–3.48 (m, 1 H), 3.39–3.34 (m, 2 H), 3.11 (d, *J* = 17.2 Hz, 1 H), 1.69 (s, 3 H). ^13^C NMR (101 MHz, CDCl_3_) δ 197.8 (s, C_q_), 158.8 (s, C_q_), 153.6 (s, C_q_), 147.5 (s, C_q_), 143.9 (s, C_q_), 137.7 (s, C_q_), 136.2 (s, C_q_), 132.6 (s, CH), 130.9 (s, C_q_), 128.6 (s, CH), 128.2 (s, CH), 128.0 (s, CH), 127.7 (s, CH), 127.6 (s, CH),126.9 (s, CH), 125.8 (s, CH), 125.6 (s, CH), 125.5 (s, C_q_), 116.4 (s, CH), 110.5 (s, CH), 109.1 (s, CH), 75.8 (s, CH_2_), 56.1 (s, CH_3_), 49.4 (s, CH_2_), 46.1 (s, CH_2_), 42.0 (s, C_q_), 24.0 (s, CH_3_). HRMS (+ESI) *m*/*z* calculated for C_33_H_30_NaO_3_ [M + Na]^+^ 497.2087, found 497.2066.

### Compound (*Z*)-3-Methyl-1,3,5-triphenyl-5-(5-(trifluoromethyl)benzofuran-3-(2*H*)-ylidene)pentan-1-one (**3d**)

Prepared according to the representative procedure A from 0.14 mmol of substrate **1f** and 0.17 mmol of 3-methyl,-1,3-diphenylcyclobutan-1-ol **(2a)**. The crude was purified by column chromatography over silica gel using 0 to 5% gradient EtOAc in *n*-hexane containing 1% Et_3_N to afford the heterocycle **3d** as a light-yellow oil (50 mg, 0.097 mmol, 69%). IR (cm^–1^) ν̅ 1688 (s), 1597 (s), 1442 (m), 1481 (m), 1333 (m), 1316 (s), 1114 (s), 734 (s), 698 (s). ^1^H NMR (300 MHz, CDCl_3_) δ 7.79 (br d, *J* = 1.8 Hz, 1 H), 7.63–7.55 (m, 2 H), 7.49–7.43 (m, 2 H), 7.41 (ddd, *J* = 8.5, 2.0, 0.8 Hz, 1 H), 7.36–7.27 (m, 5 H), 7.24–7.21 (m, 2 H), 7.19–7.14 (m, 3 H), 7.10–7.04 (m, 1 H), 6.85–6.82 (m, 1 H), 5.02–4.89 (m, 2 H), 3.47–3.26 (m, 3 H), 3.08 (d, *J* = 17.3 Hz, 1 H), 1.66 (s, 3 H). ^13^C NMR (75.45 MHz, CDCl_3_) δ 197.7 (s, C_q_), 166.5 (q, *J*_*CF*_ = 1.0 Hz, C_q_), 146.7 (s, C_q_), 143.5 (s, C_q_), 137.7 (s, C_q_), 134.2 (s, C_q_), 133.2 (s, C_q_), 132.6 (s, CH), 128.8 (s, CH), 128.3 (s, CH), 128.2 (s, CH), 127.7 (s, CH), 127.4 (s, CH), 127.3 (s, CH), 127.2 (q, *J*_*CF*_ = 3.3 Hz, CH), 126.0 (s, CH), 125.7 (s, C_q_), 125.5 (s, CH), 122.6 (q, *J*_*CF*_ = 32,1 Hz, C_q_), 121.3 (q, *J*_*CF*_ = 3.9 Hz, CH), 110.4 (s, CH), 76.3 (s, CH_2_), 48.9 (s, CH_2_), 46.7 (s, CH_2_), 42.2 (s, C_q_), 24.4 (s, CH_3_). One quaternary carbon signal is overlapped. ^19^F-NMR (376.5 MHz, CDCl_3_) δ −61.02 (s). HRMS (+ESI) *m*/*z* calculated for C_33_H_27_F_3_NaO_2_ [M + Na]^+^ 535.1855, found 535.1850.

### Compound (*Z*)-5-(5-Fluorobenzofuran-3(2*H*)-ylidene)-3-methyl-1,3,5-triphenylpentan-1-one (**3e**)

Prepared according to the representative procedure A from 0.14 mmol of substrate **1e** and 0.17 mmol of 3-methyl,-1,3-diphenylcyclobutan-1-ol (**2a**). The crude was purified by column chromatography over silica gel using 0 to 10% gradient EtOAc in *n*-hexane containing 1% Et_3_N to afford the heterocycle **3e** as a yellow oil (39 mg, 0.084 mmol, 60%). IR (cm^–1^) ν̅ 1690 (s), 1597 (s), 1474 (s), 1323 (s), 1117 (s), 743 (s), 697 (s). ^1^H NMR (300 MHz, CDCl_3_) δ 7.59–7.52 (m, 2 H), 7.52–7.40 (m, 1 H), 7.33 (dt, *J* = 8.4, 0.9 Hz, 4 H), 7.31–7.26 (m, 2 H), 7.26–7.24 (m, 1 H), 7.24–7.16 (m, 3 H), 7.15–7.09 (m, 2 H), 7.09–7.04 (m, 1 H), 6.86 (td, *J* = 8.7, 2.7 Hz, 1 H), 6.71 (dd, *J* = 8.8, 4.4 Hz, 1 H), 4.90 (s, 2 H), 3.46–3.27 (m, 3 H), 3.09 (d, *J* = 17.2 Hz, 1 H), 1.66 (s, 3 H). ^13^C NMR (75.45 MHz, CDCl_3_) δ 197.8 (s, C_q_), 160.3 (s, C_q_), 156.9 (d, J_CF_ = 235.4 Hz, C_q_), 147.0 (s, C_q_), 143.5 (s, C_q_), 137.7 (s, C_q_), 135.4 (d, *J* = 2.9 Hz, C_q_), 132.6 (s, CH), 132.2 (s, C_q_), 128.7 (s, CH), 128.2 (s, CH), 128.1 (s, CH), 127.7 (s, CH), 127.5 (s, CH), 127.1 (s, CH), 125.9 (s, CH), 125.6 (s, CH), 116.0 (d, *J* = 24.6 Hz, CH), 110.8 (d, *J* = 26.5 Hz, CH), 110.4 (d, *J* = 8.7 Hz, CH), 76.1 (s, CH_2_), 49.3 (s, CH_2_), 46.0 (s, CH_2_), 42.0 (s, C_q_), 24.3 (s, CH_3_). The signal of one C_q_ is overlapped. ^19^F-NMR (376.5 MHz, CDCl_3_) δ −123.57 (s). HRMS (+ESI) *m*/*z* calculated for C_32_H_27_FNaO_2_ [M + Na]^+^ 485.1887, found 485.1868.

### Compound (*Z*)-5-(Furo[3,2-*b*]pyridin-3(2*H*)-ylidene)-3-methyl-1,3,5-triphenylpentan-1-one (**3f**)

Prepared according to the representative procedure A from 0.10 mmol of substrate **1g** and 0.12 mmol of 3-methyl,-1,3-diphenylcyclobutan-1-ol **(2a)**. The crude was purified by column chromatography over silica gel using 0 to 10% gradient EtOAc in *n*-hexane containing 1% Et_3_N to afford the heterocycle **3f** as a light-yellow oil (22 mg, 0.05 mmol, 49%). IR (cm^–1^) ν̅ 1690 (s), 1597 (s), 1436 (s), 1253 (s), 798 (s), 699 (s). ^1^H NMR (300 MHz, CDCl_3_) δ 8.21 (t, *J* = 3.1 Hz, 1 H), 7.58 (dd, *J* = 8.4, 1.4 Hz, 2 H), 7.45–7.31 (m, 3 H), 7.27–7.15 (m, 6 H), 7.15–7.07 (m, 2 H), 7.06–6.97 (m, 4 H), 5.18–4.74 (m, 2 H), 4.10–3.99 (m, 1 H), 3.89 (d, *J* = 17.1 Hz, 1 H), 3.64 (d, *J* = 13.0 Hz, 1 H), 3.22 (d, *J* = 17.1 Hz, 1 H), 1.41 (s, 3 H). ^13^C NMR (75.45 MHz, CDCl_3_) δ 198.8 (s, C_q_), 158.5 (s, C_q_), 148.0 (s, C_q_), 147.7 (s, C_q_), 143.3 (s, C_q_), 141.6 (s, CH), 138.2 (s, C_q_), 136.3 (s, C_q_), 132.9 (s, C_q_), 132.3 (s, CH), 128.5 (s, CH), 128.1 (s, CH), 127.8 (s, CH), 127.7 (s, CH), 127.3 (s, CH) 127.2 (s, CH), 126.2 (s, CH), 125.4 (s, CH), 123.0 (s, CH), 116.4 (s, CH), 75.1 (s, CH_2_), 48.4 (s, CH_2_), 45.1 (s, CH_2_), 42.7 (s, C_q_), 24.8 (s, CH_3_). HRMS (+ESI) *m*/*z* calculated for C_31_H_27_NNaO_2_ [M + Na]^+^ 468.1934, found 468.1927.

### Compound (*Z*)-5-(Benzofuran-3(2*H*)-ylidene)-1,5-diphenylpentan-1-one (**3g**)

Prepared according to the representative procedure A from 0.14 mmol of substrate **1a** and 0.17 mmol of 1-phenylcyclobutan-1-ol **(2b)**. The crude was purified by column chromatography over silica gel using 0 to 10% gradient EtOAc in *n*-hexane containing 1% Et_3_N to afford the heterocycle **3g** as a yellow oil (28 mg, 0.08 mmol, 56%). IR (cm^–1^) ν̅ 1678 (s), 1595, 1497 (s), 1231 (s), 1123 (s), 998 (s), 752 (s), 697 (s). ^1^H NMR (300 MHz, CDCl_3_) δ 7.96–7.89 (m, 2 H), 7.65 (dd, *J* = 7.7, 1.3 Hz, 1 H), 7.57–7.51 (m, 1 H), 7.46–7.43 (m, 2 H), 7.39–7.40 (m, 1 H), 7.38–7.35 (m, 1 H), 7.31–7.24 (m, 1 H), 7.23–7.19 (m, 2 H), 7.18–7.15 (m, 1 H), 6.93 (td, *J* = 7.6, 1.1 Hz, 1 H), 6.84 (dd, *J* = 8.0, 1.0 Hz, 1 H), 4.91 (s, 2 H), 3.04 (t, *J* = 7.1 Hz, 2 H), 2.90–2.84 (m, 2 H), 2.03–1.93 (m, 2 H). ^13^C NMR (75.45 MHz, CDCl_3_) δ 199.8 (s, C_q_), 164.1 (s, C_q_), 142.9 (s, C_q_), 136.9 (s, C_q_), 133.3 (s, C_q_), 133.0 (s, CH), 132.5 (s, C_q_), 129.5 (s, CH), 128.8 (s, CH), 128.5 (s, CH), 128.0 (s, CH), 127.4 (s, CH), 127.2 (s, CH), 125.5 (s, C_q_), 124.1 (s, CH), 120.7 (s, CH), 110.4 (s, CH), 75.1 (s, CH_2_), 38.0 (s, CH_2_), 33.6 (s, CH_2_), 17.0 (s, CH_2_). HRMS (+ESI) *m*/*z* calculated for C_25_H_22_NaO_2_ [M + Na]^+^ 377.1512, found 377.1494.

### Compound (*Z*)-5-(Benzofuran-3(2*H*)-ylidene)-1-(4-fluorophenyl)-5-phenylpentan-1-one (**3h**)

Prepared according to the representative procedure A from 0.12 mmol of substrate **1b** and 0.14 mmol of 1-(4-fluorophenyl)cyclobutan-1-ol **(2d)**. The crude was purified by column chromatography over silica gel using 0 to 5% gradient EtOAc in *n*-hexane containing 1% Et_3_N to afford the heterocycle **3h** as a light-yellow oil (32 mg, 0.086 mmol, 72%). IR (cm^–1^) ν̅ 3060 (m), 2933 (m), 1682 (s), 1599 (s), 1454 (m), 1408 (m), 1228 (s), 1156 (m), 1098 (w), 832 (w), 747 (s), 700 (s). ^1^H NMR (300 MHz, CDCl_3_) δ 8.00–7.87 (m, 2 H), 7.65 (dd, *J* = 7.8, 1.3 Hz, 1 H), 7.43–7.34 (m, 2 H), 7.32–7.27 (m, 1 H), 7.24–7.15 (m, 3 H), 7.15–7.05 (m, 2 H), 6.93 (td, *J* = 7.6, 1.1 Hz, 1 H), 6.85 (dt, *J* = 8.1, 0.7 Hz, 1 H), 4.91 (s, 2 H), 3.01 (t, *J* = 7.1 Hz, 2 H), 2.87 (tt, *J* = 7.3, 1.4 Hz, 2 H), 2.22–1.89 (m, 2 H). ^13^C NMR (75.45 MHz, CDCl_3_) δ 198.3 (s, C_q_), 165.6 (d, J_CF_ = 254.3 Hz, C_q_), 164.2 (s, C_q_), 142.9 (s, C_q_), 133.3 (d, J_CF_ = 3.1 Hz, C_q_), 133.2 (s, C_q_), 132.6 (s, C_q_), 131.6 (d, *J*_CF_ = 9.4 Hz, CH), 129.6 (s, CH), 128.8 (s, CH), 127.4 (s, CH), 127.3 (s, CH), 125.5 (s, C_q_), 124.0 (s, CH), 120.7 (s, CH), 116.6 (d, *J*_CF_ = 21.8 Hz, CH), 110.4 (s, CH), 75.2 (s, CH_2_), 37.9 (s, CH_2_), 33.6 (s, CH_2_), 22.4 (s, CH_2_). ^19^F-NMR (282.4 MHz, CDCl_3_) δ −104.7 (s). HRMS (+ESI) *m*/*z* calculated for C_25_H_21_FNaO_2_ [M + Na]^+^ 395.1418, found 395.1406.

### Compound (*Z*)-5-(Benzofuran-3(2*H*)-ylidene)-5-phenyl-1-(*p*-tolyl)pentan-1-one (**3i**)

Prepared according to the representative procedure A from 0.12 mmol of substrate **1b** and 0.14 mmol of 1-(*p*-tolyl)cyclobutan-1-ol **(2e)**. The crude was purified by column chromatography over alumina using 0 to 5% gradient EtOAc in *n*-hexane containing 1% Et_3_N to afford the heterocycle **3i** as a light-yellow oil (34 mg, 0.09 mmol, 76%). IR (cm^–1^) ν̅ 2924 (m), 1683 (s), 1603 (s), 1464 (s), 1362 (m), 1226 (s), 1181 (m), 985 (m), 807 (s), 746 (s). ^1^H NMR (300 MHz, CDCl_3_) δ 7.85–7.76 (m, 2 H), 7.69–7.55 (m, 1 H), 7.38 (ddd, *J* = 7.7, 6.6, 1.3 Hz, 2 H), 7.32–7.13 (m, 6 H), 6.99–6.89 (m, 1 H), 6.87–6.76 (m, 1 H), 4.91 (br s, 2 H), 3.01 (td, *J* = 7.2, 1.1 Hz, 2 H), 2.93–2.62 (m, 2 H), 2.40 (s, 3 H), 2.06–1.78 (m, 2 H). ^13^C NMR (75.45 MHz, CDCl_3_) δ 199.6 (s, C_q_), 164.2 (s, C_q_), 152.2 (s, C_q_), 143.7 (s, C_q_), 143.0 (s, C_q_), 134.5 (s, C_q_), 133.4 (s, C_q_), 132.5 (s, C_q_), 129.5 (s, CH), 129.2 (s, CH), 128.8 (s, CH), 128.1 (s, CH),127.4 (s, CH), 127.2 (s, CH), 124.1 (s, CH), 120.7 (s, CH), 110.4 (s, CH), 75.2 (s, CH_2_), 38.0 (s, CH_2_), 33.7 (s, CH_2_), 22.6 (s, CH_2_), 21.6 (s, CH_3_). HRMS (+ESI) *m*/*z* calculated for C_26_H_24_NaO_2_ [M + Na]^+^ 391.1669, found 391.1656.

### Compound (*Z*)-5-(Furo[3,2-*b*]pyridine-3(2*H*)-ylidene)-1,5-diphenylpentan-1-one (**3j**)

Prepared according to the representative procedure A from 0.14 mmol of substrate **1g** and 0.17 mmol of 1-phenylcyclobutan-1-ol **(2b)**. The crude was purified by column chromatography over silica gel using 0 to 10% gradient EtOAc in *n*-hexane containing 1% Et_3_N to afford the heterocycle **3j** as a yellow oil (23 mg, 0.065 mmol, 46%). IR (cm^–1^) ν̅ 1678 (s), 1594 (s), 1427 (s), 1258 (m), 1125 (m), 897 (s), 764 (s), 699 (s). ^1^H NMR (600 MHz, CDCl_3_) δ 8.05 (dd, *J* = 4.8, 1.5 Hz, 1 H), 7.84 (dd, *J* = 8.4, 1.3 Hz, 2 H), 7.44 (ddt, *J* = 8.7, 7.1, 1.3 Hz, 1 H), 7.36–7.29 (m, 4 H), 7.22 (tt, *J* = 7.0, 1.4 Hz, 1 H), 7.20–7.15 (m, 2 H), 6.99–6.90 (m, 2 H), 4.97 (s, 2 H), 3.60–3.19 (m, 2 H), 3.13–2.80 (m, 2 H), 2.16–1.59 (m, 2 H). ^13^C NMR (151 MHz, CDCl_3_) δ 200.5 (s, C_q_), 158.3 (s, C_q_), 148.0 (s, C_q_), 141.9 (s, C_q_), 141.8 (s, CH), 138.2 (s, C_q_), 137.1 (s, C_q_), 133.1 (s, CH), 129.9 (s, C_q_), 128.8 (s, CH), 128.4 (s, CH), 128.1 (s, CH), 127.5 (s, CH), 127.1 (s, CH), 122.7(s, CH), 116.22 (s, CH), 74.8 (s, CH_2_), 38.2 (s, CH_2_), 31.7 (s, CH_2_), 23.3 (s, CH_2_). HRMS (+ESI) *m*/*z* calculated for C_24_H_22_NO_2_ [M + H]^+^ 356.1645, found 356.1654.

### Compound (*Z*)-7-(Benzofuran-3(2*H*)-ylidene)-2-methyl-7-phenylheptan-3-one (**3l**)

Prepared according to the representative procedure A from 0.12 mmol of substrate **1b** and 0.14 mmol of 1-isopropyl-clobutan-1-ol **(2f)**. The crude was purified by column chromatography over silica gel using 0 to 10% gradient EtOAc in *n*-hexane containing 1% Et_3_N to afford the heterocycle **3l** as a light-yellow oil (17 mg, 0.053 mmol, 44%). IR (cm^–1^) ν̅ 1708 (s), 1606 (s), 1586 (s), 1465 (s), 1223 (m), 1128 (m), 1087 (m), 755 (s), 697 (s). ^1^H NMR (300 MHz, CDCl_3_) δ 7.66–7.63 (m, 1 H), 7.43–7.35 (m, 2 H), 7.31–7.27 (m, 1 H), 7.22–7.16 (m, 3 H), 6.97 (td, *J* = 7.6, 1.1 Hz, 1 H), 6.84 (ddd, *J* = 8.0, 1.1, 0.5 Hz, 1 H), 4.90 (s, 2 H), 2.79–2.74 (m, 2 H), 2.57–2.48 (m, 3 H), 1.85–1.75 (m, 2 H), 1.05 (d, *J* = 6.9 Hz, 6 H). ^13^C NMR (151 MHz, CDCl_3_) δ 214.4 (s, C_q_), 164.2 (s, C_q_), 143.0 (s, C_q_), 133.4 (s, C_q_), 132.5 (s, C_q_), 129.6 (s, CH), 128.8 (s, CH), 127.4 (s, CH), 127.2 (s, CH), 125.5 (s, C_q_), 124.1 (s, CH), 120.7 (s, CH), 110.4 (s, CH), 75.1 (s, CH_2_), 40.8 (s, CH), 39.6 (s, CH_2_), 33.5 (s, CH_2_), 21.9 (s, CH_2_), 18.2 (s, CH_3_). HRMS (+ESI) *m*/*z* calculated for C_22_H_24_NaO_2_ [M + Na]^+^ 343.1668, found 343.1659.

### Compound (*Z*)-5-(Benzofuran-3(2*H*)-ylidene)-1-phenyl-5-(trimethylsilyl)pentan-1-one (**3m**)

Prepared according to the representative procedure A from 0.14 mmol of substrate **1h** and 0.17 mmol of 1-phenylcyclobutan-1-ol **(2b)**. The crude was purified by column chromatography over silica gel using 0 to 10% gradient EtOAc in *n*-hexane containing 1% Et_3_N to afford the heterocycle **3m** as a light-yellow oil (28 mg, 0.08 mmol, 57%). IR (cm^–1^) ν̅ 1685 (s), 1648 (s), 1498 (s), 1379 (s), 1253 (m), 1124 (m), 876 (s), 787 (s), 695 (s). ^1^H NMR (400 MHz, CDCl_3_) δ 7.80–7.77 (m, 2 H), 7.40–7.35 (m, 2 H), 7.29–7.25 (m, 2 H), 6.99–6.95 (m, 1 H), 6.68–6.64 (m, 2 H), 4.87 (s, 2 H), 2.90 (t, *J* = 7.1 Hz, 2 H), 2.40 (ddd, *J* = 11.5, 4.8, 2.8 Hz, 2 H), 1.75–1.67 (m, 2 H), 0.00 (s, 9 H). ^13^C NMR (100.1 MHz, CDCl_3_) δ 200.0 (s, C_q_), 164.5 (s, C_q_), 144.2 (s, C_q_), 136.9 (s, C_q_), 133.0 (s, CH), 131.1 (s, C_q_), 129.9 (s, CH), 128.6 (s, CH), 128.1 (s, CH), 126.2 (s, C_q_), 125.3 (s, CH), 120.6 (s, CH), 110.6 (s, CH), 75.1 (s, CH_2_), 38.6 (s, CH_2_), 31.2 (s, CH_2_), 23.40 (s, CH_2_), 0.74 (s, CH_3_). HRMS (+ESI) *m*/*z* calculated for C_22_H_27_O_2_Si [M + H]^+^ 351.1780, found 351.1769.

### Compound (*Z*)-3-Methyl-1,3,5-triphenyl-5-(1-tosylindolin-3-ylidene)pentan-1-one (**3o**)

Prepared according to the representative procedure A from 0.14 mmol of substrate **1j** and 0.17 mmol of 1,3-diphenylcyclobutan-1-ol **(2a)**. The crude was purified by column chromatography over silica gel using 0 to 30% gradient EtOAc in *n*-hexane containing 1% Et_3_N to afford the heterocycle **3o** as a yellow oil (60 mg, 0.10 mmol, 71%). IR (cm^–1^) ν̅ 1693 (s), 1593 (s), 1489 (s), 1365 (s), 1136 (s), 905 (s), 763 (s), 698 (s). ^1^H NMR (300 MHz, CDCl_3_) δ 7.75–7.64 (m, 3 H), 7.57–7.51 (m, 4 H), 7.51–7.48 (m, 1 H), 7.48–7.41 (m, 3 H), 7.35–7.27 (m, 3 H), 7.25–7.17 (m, 4 H), 7.17–7.12 (m, 2 H), 7.11–7.05 (m, 1 H), 7.05–6.99 (m, 2 H), 4.37–4.26 (m, 2 H), 3.50–3.17 (m, 3 H), 2.99 (d, *J* = 17.2 Hz, 1H), 2.38 (s, 3 H), 1.56 (s, 3 H). ^13^C NMR (75.45 MHz, CDCl_3_) δ 197.7 (s, C_q_), 147.2 (s, C_q_), 145.1 (s, C_q_), 144.0 (s, C_q_), 143.6 (s, C_q_), 137.6 (s, C_q_), 133.9 (s, C_q_), 133.8 (s, C_q_), 132.6 (s, CH), 129.6 (s, CH), 129.1 (s, CH), 128.8 (s, CH), 128.2 (s, CH), 128.0 (s, CH), 127.64 (s, CH), 127.60 (s, CH), 127.19 (s, CH), 127.15 (s, CH), 125.7 (s, CH), 125.5 (s, CH), 124.3 (s, CH), 123.6 (s, CH), 115.6 (s, CH), 55.8 (s, CH_2_), 49.4 (s, CH_2_), 45.8 (s, CH_2_), 41.7 (s, C_q_), 24.0 (s, CH_3_), 21.5 (s, CH_3_). Some C_q_ signals are overlapped. HRMS (+ESI) *m*/*z* calculated for C_39_H_35_NNaO_3_S [M + Na]^+^ 620.2230, found 620.2202.

### Compound (*Z*)-5-(Isochroman-4-ylidene)-3-methyl-1,3,5-triphenylpentan-1-one (**3q**)

Prepared according to the representative procedure A from 0.12 mmol of substrate **1k** and 0.14 mmol of 3-methyl,-1,3-diphenylcyclobutan-1-ol **(2a)**. The crude was purified by column chromatography over silica gel using 0 to 10% gradient EtOAc in *n*-hexane containing 1% Et_3_N to afford the heterocycle **3q** as a white solid (40 mg, 0.087 mmol, 73%). mp 130 °C. IR (cm^–1^) ν̅ 1690 (s), 1589 (s), 1494 (s), 1436 (s), 1224 (s), 1112 (s), 1024 (s), 757 (s), 692 (s). ^1^H NMR (300 MHz, CDCl_3_) δ 7.53–7.48 (m, 2 H), 7.49–7.42 (m, 1 H), 7.34–7.27 (m, 4 H), 7.25–7.20 (m, 2 H), 7.19–7.10 (m, 8 H), 7.08–7.06 (m, 2 H), 4.57 (s, 2 H), 4.14–4.05 (m, 2 H), 3.47–3.37 (m, 2 H), 3.23 (d, *J* = 17.2 Hz, 1 H), 2.90 (d, *J* = 17.2 Hz, 1 H), 1.44 (s, 3 H). ^13^C NMR (75.45 MHz, CDCl_3_) δ 197.9 (s, C_q_), 147.0 (s, C_q_), 142.1 (s, C_q_), 137.8 (s, C_q_), 137.2 (s, C_q_), 137.1 (s, C_q_), 134.7 (s, C_q_), 132.5 (s, CH), 131.8 (s, C_q_), 129.1 (s, CH), 128.3 (s, CH), 128.1 (s, CH), 127.9 (s, CH), 127.7 (s, CH), 127.6 (s, CH), 127.0 (s, CH), 126.9 (s, CH), 126.2 (s, CH), 125.9 (s, CH), 125.6 (s, CH), 124.6 (s, CH), 67.7 (s, CH_2_), 67.1 (s, CH_2_), 49.5 (s, CH_2_), 46.4 (s, CH_2_), 41.8 (s, C_q_), 25.4 (s, CH_3_). HRMS (+ESI) *m*/*z* calculated for C_33_H_30_NaO_2_ [M + Na]^+^ 481.2138, found 481.2146.

### Compound (*Z*)-3-Methyl-1,3,5-triphenyl-5-(2-tosyl-2,3-dihydroisoquinolin-4(*1H*)-ylidene)pentan-1-one (**3r**)

Prepared according to the representative procedure A from 0.14 mmol of substrate **1l** and 0.17 mmol of 3-methyl-1,3-diphenylcyclobutan-1-ol **(2a)**. The crude was purified by column chromatography over silica gel using 0 to 15% gradient EtOAc in *n*-hexane containing 1% Et_3_N to afford the heterocycle **3r** as a yellow oil (60 mg, 0.10 mmol, 70%). IR (cm^–1^) ν̅ 1688 (s), 1597 (s), 1462 (s), 1158 (s), 905 (s), 726 (s), 699 (s). ^1^H NMR (300 MHz, CDCl_3_) δ 7.53 (dd, *J* = 8.3, 1.4 Hz, 2 H), 7.48–7.40 (m, 3 H), 7.32 (dd, *J* = 8.2, 7.1 Hz, 2 H), 7.21 (m, 5 H), 7.14 (d, *J* = 8.4 Hz, 3 H), 7.10–7.02 (m, 4 H), 7.00–6.93 (m, 2 H), 6.91–6.85 (m, 2 H), 4.08 (m, 2 H), 3.75–3.56 (m, 2 H), 3.51–3.28 (m, 2 H), 3.09 (d, *J* = 17.1 Hz, 1 H), 2.84 (d, *J* = 17.0 Hz, 1 H), 2.37 (s, 3 H), 1.22 (s, 3 H). ^13^C NMR (75.45 MHz, CDCl_3_) δ 197.9 (s, C_q_), 146.6 (s, C_q_), 143.0 (s, C_q_), 141.0 (s, C_q_), 139.02 (s, C_q_), 137.8 (s, C_q_), 136.3 (s, C_q_), 134.5 (s, C_q_), 135.0 (s, C_q_), 132.6 (s, CH), 129.9 (s, C_q_), 129.4 (s, CH), 128.9 (s, CH), 128.4 (s, CH), 128.2 (s, CH), 127.8 (s, CH), 127.74 (s, CH), 127.70 (s, CH), 127.3 (s, CH), 127.2 (s, CH), 127.1 (s, CH), 126.8 (s, CH), 126.3 (s, CH), 125.8 (s, CH), 125.6 (s, CH), 49.1 (s, CH_2_), 47.6 (s, CH_2_), 45.1 (s, CH_2_), 41.8 (s, CH_2_), 29.7 (s, C_q_), 25.7 (s, CH_3_), 21.5 (s, CH_3_). HRMS (+ESI) *m*/*z* calculated for C_40_H_38_NO_3_S [M + H]^+^ 612.2567, found 612.2568.

### Compound (*E*)-1-Methyl-3-(3-methyl-5-oxo-1,3,5-triphenylpentylidene)indolin-2-one (**3s**)

Prepared according to the representative procedure A from 0.14 mmol of substrate **1m** and 0.17 mmol of 3-methyl-1,3-diphenylcyclobutan-1-ol **(2a)**. The crude was purified by column chromatography over silica gel using 0 to 35% gradient EtOAc in *n*-hexane containing 1% Et_3_N to afford the heterocycle **3s** as a yellow oil (38 mg, 0.081 mmol, 58%). IR (cm^–1^) ν̅ 1694 (s), 1616 (s), 1595 (s), 1490 (s), 1122 (s), 904 (s), 787 (s), 693 (s). ^1^H NMR (600 MHz, CDCl_3_) δ 7.69–7.67 (m, 2 H), 7.45 (ddt, *J* = 8.7, 7.1, 1.3 Hz, 1 H), 7.40–7.37 (m, 2 H), 7.35–7.31 (m, 3 H), 7.31–7.28 (m, 3 H), 7.16–7.10 (m, 4 H), 7.04 (ddt, *J* = 7.7, 6.9, 1.2 Hz, 1 H), 6.75 (ddd, *J* = 7.8, 1.0, 0.5 Hz, 1 H), 6.57 (td, *J* = 7.7, 1.1 Hz, 1 H), 6.06–5.99 (m, 1 H), 4.17 (d, *J* = 13.2 Hz, 1 H), 4.06 (d, *J* = 13.2 Hz, 1 H), 3.69 (d, *J* = 17.0 Hz, 1 H), 3.30 (s, 3 H), 3.24 (d, *J* = 17.1 Hz, 1 H), 1.48 (s, 3 H). ^13^C NMR (151 MHz, CDCl_3_) δ 198.3 (s, C_q_), 168.0 (s, C_q_), 157.2 (s, C_q_), 147.4 (s, C_q_), 142.2 (s, C_q_), 141.5 (s, C_q_), 138.0 (s, C_q_), 132.4 (s, CH), 128.8 (s, CH), 128.44 (s, CH), 128.37 (s, CH), 128.2 (s, CH), 127.9 (s, CH), 127.8 (s, CH), 127.6 (s, CH), 126.2 (s, CH), 125.6 (s, CH), 123.1 (s, CH), 122.8 (s, C_q_), 121.4 (s, CH), 107.4 (s, CH), 49.3 (s, CH_2_), 46.2 (s, CH_2_), 42.5 (s, C_q_), 25.9 (s, CH_3_), 24.8 (s, CH_3_). Some signals are overlapped. HRMS (+ESI) *m*/*z* calculated for C_33_H_30_NO_2_ [M + H]^+^ 472.2271, found 472.2276.

### Compound (*E*)-1,5-Dimethyl-3-(5-oxo-1,5-diphenylpentylidene)indolin-2-one (**3t**)

Prepared according to the representative procedure A from 0.14 mmol of substrate **1n** and 1-phenylcyclobutan-1-ol **(2b)**. The crude was purified by column chromatography over silica gel using 0 to 20% gradient EtOAc in *n*-hexane containing 1% Et_3_N to afford the 3-alkylideneoxindole **3t** as a yellow oil (25 mg, 0.063 mmol, 45%). IR (cm^–1^) ν̅ 1683 (s), 1646 (s), 1617 (s), 1593 (s), 1489 (s), 1368 (m), 1325 (m), 1098 (m), 767 (s), 698 (s). ^1^H NMR (300 MHz, CDCl_3_) δ 7.95–7.92 (m, 2 H), 7.56–7.40 (m, 6 H), 7.29–7.26 (m, 2 H), 6.94 (ddd, *J* = 7.9, 1.7, 0.8 Hz, 1 H), 6.64 (d, *J* = 7.9 Hz, 1 H), 5.84–5.83 (m, 1 H), 3.46–3.41 (m, 2 H), 3.23 (s, 3 H), 3.14–3.09 (m, 2 H), 2.01–1.91 (m, 5 H). Some signals are overlapped. ^13^C NMR (75.45 MHz, CDCl_3_) δ 200.0 (s, C_q_), 167.8 (s, C_q_), 157.9 (s, C_q_), 141.2 (s, C_q_), 140.2 (s, C_q_), 137.0 (s, C_q_), 132.8 (s, CH), 130.6 (s, C_q_), 129.1 (s, CH), 128.53 (s, CH), 128.45 (s, CH), 128.4 (s, CH), 128.03 (s, CH), 126.9 (s, CH), 124.0 (s, C_q_), 123.9 (s, CH), 122.6 (s, C_q_), 107.1 (s, CH), 38.3 (s, CH_2_), 34.1 (s, CH_2_), 25.7 (s, CH_3_), 22.5 (s, CH_2_), 21.1 (s, CH_3_). HRMS (+ESI) *m*/*z* calculated for C_27_H_26_NO_2_ [M + H]^+^ 396.1958, found 396.1964.

### Compound (*E*)-5-Chloro-1-methyl-3-(5-oxo-1,5-diphenylpentylidene)indolin-2-one (**3u**)

Prepared according to the representative procedure A from 0.14 mmol of substrate **1o** and 0.17 mmol of 1-phenylcyclobutan-1-ol **(2b)**. The crude was purified by column chromatography over silica gel using 0 to 25% gradient EtOAc in *n*-hexane containing 1% Et_3_N to afford the heterocycle **3u** as a yellow oil (25 mg, 0.06 mmol, 43%). IR (cm^–1^) ν̅ 1685 (s), 1602 (s), 1498 (s), 1338 (m), 1098 (m), 778 (s), 697 (s). ^1^H NMR (300 MHz, CDCl_3_) δ 7.93 (m, 2 H), 7.55–7.48 (m, 4 H), 7.45–7.41 (m, 2 H), 7.27–7.25 (m, 3 H), 6.66 (d, *J* = 8.4 Hz, 1 H), 5.98 (d, *J* = 2 Hz, 1 H), 3.47–3.39 (m, 2 H), 3.24 (s, 3 H), 3.11 (t, *J* = 7.6 Hz, 2 H), 1.96 (q, *J* = 7.8 Hz, 2 H). ^13^C NMR (100.1 MHz, CDCl_3_) δ 199.8 (s, C_q_), 167.4 (s, C_q_), 160.3 (s, C_q_), 140. Eight (s, C_q_), 140.5 (s, C_q_), 136.9 (s, C_q_), 132.9 (s, CH), 129.3 (s, CH), 128.9 (s, CH), 128.5 (s, CH), 128.0 (s, CH), 127.9 (s, CH), 126.74 (s, C_q_), 126.70 (s, CH), 123.9 (s, C_q_), 123.24 (s, CH), 108.2 (s, CH), 38.3 (s, CH_2_), 34.3 (s, CH_3_), 25.8 (s, CH_2_), 22.4 (s, CH_2_). Some signals are overlapped. HRMS (+ESI) *m*/*z* calculated for C_26_H_23_ClNO_2_ [M + H]^+^ 416.1412, found 416.1421.

### Synthesis of Complex **4**

A Carius tube was charged with the substrate **1b** (100 mg, 0.30 mmol), [Pd(PPh_3_)_4_] (350 mg, 0.30 mmol), and a magnetic stirrer. The tube was set under a nitrogen atmosphere, and dry CH_2_Cl_2_ was added (7 mL). The tube was sealed, and the mixture was stirred at 50 °C for 18 h. After the tube was cooled, the solution was filtered through a Celite plug. The filtrate was concentrated to ca. 2 mL, and *n*-pentane (15 mL) was added. The suspension was filtered, and the solid was washed with *n*-pentane (2 × 3 mL) and air-dried to give crude **4** as a bright yellow solid. Yield: 243 mg, 0.25 mmol, 84%. Crude complex **4** was recrystallized from CH_2_Cl_2_/Et_2_O to give analytically pure **4**. mp 204 °C (dec). ^1^H NMR (400.9 MHz, CDCl_3_) δ 9.24 (d, ^3^*J*_HH_ = 7.2 Hz, 1 H, H6, C_6_H_4_), 7.52–7.42 (m, 12 H, *o*-H, PPh_3_), 7.37–7.30 (m, 6 H, *p*-H, PPh_3_), 7.25–7.18 (m, 12 H, *m*-H, PPh_3_), 7.03 (td, ^3^*J*_HH_ = 7.8, ^4^*J*_HH_ = 1.2 Hz, 1 H, H4, C_6_H_4_), 6.98 (“t”, ^3^*J*_HH_ = 7.3 Hz, 1 H, *p*-H, Ph), 6.87 (td, ^3^*J*_HH_ = 7.4, ^4^*J*_HH_ = 0.8 Hz, 1 H, H5, C_6_H_4_), 6.84 (t, ^3^*J*_HH_ = 7.7 Hz, 2 H, *m*-H, Ph), 6.51 (d, ^3^*J*_HH_ = 7.3 Hz, 2 H, *o*-H, Ph), 6.45 (“d″, ^3^*J*_HH_ = 7.7 Hz, 1 H, H3, C_6_H_4_), 4.35 (“t”, ^2^*J*_HH_ = 3.2 Hz, 2 H, CH_2_). ^13^C NMR (100.8 MHz, CDCl_3_) δ 163.3 (s, C2), 155.5 (t, *J*_PH_ = 2.1 Hz, C_q_), 143.9 (t, *J*_PH_ = 2.9 Hz, *i*-C, Ph), 135.2 (t, *J*_PH_ = 5.9 Hz, *o*-CH, PPh_3_), 134.4 (t, *J*_PH_ = 5.1 Hz, C_q_), 131.9 (t, *J*_PH_ = 22.9 Hz, *i*-C, PPh_3_), 130.2 (s, C1), 130.0 (s, *p*-CH, PPh_3_), 129.0 (s, *o*-CH, Ph), 128.7 (s, CH4, C_6_H_4_), 127.4 (t, *J*_PH_ = 5.0 Hz, *m*-CH, PPh_3_), 126.9 (s, *m*-CH, Ph), 125.6 (s, *p*-CH, Ph), 121.9 (s, CH6, C_6_H_4_), 119.4 (s, CH5, C_6_H_4_), 109.1 (s, CH3), 77.1 (s, CH_2_). IR (Nujol, cm^–1^) ν̅ 1590 (w), 1231 (m), 1093 (m), 742 (s), 691 (s), 520 (s), 509 (s), 494 (m). Anal. Calcd for C_51_H_41_IOP_2_Pd: C, 63.47; H, 4.28. Found: C, 63.55; H, 4.33.

### Single-Crystal X-ray Structure Determination

Single crystals of complex **4**, suitable for an X-ray diffraction study, were obtained by slow diffusion of *n*-pentane into a solution of **4** in CHCl_3_.

#### Data Collection

A crystal suitable for X-ray diffraction was mounted in inert oil on a glass fiber and transferred to a Bruker diffractometer. Data were recorded at 100(2) K, using graphite-monochromated Mo Kα radiation (λ = 0.71073 Å) and omega and phi scan mode. Multiscan absorption correction was applied.

#### Structure Solution and Refinements

The crystal structure was solved by dual method, and all non-hydrogen atoms were refined anisotropically on *F*^2^ using the program SHELXL-2018/3.^70^ Hydrogen atoms were refined using the riding model.

## Supporting Information Available

Experimental procedures and compound characterization for staring materials **1** and NMR spectra of the new compounds (PDF)

## Accession Codes

CCDC 2132049 contains the supplementary crystallographic data for this paper. These data can be obtained free of charge via www.ccdc.cam.ac.uk/data_request/cif, or by emailing data_request@ccdc.cam.ac.uk, or by contacting The Cambridge Crystallographic Data Centre, 12 Union Road, Cambridge CB2 1EZ, UK; fax: +44 1223 336033.
