# Supplementary material for: Synthesis of Benzofused O- and N-Heterocycles through Cascade Carbopalladation/Cross-Alkylation of Alkynes Involving the C–C Cleavage of Cyclobutanols
Source: Organometallics. 2022 Mar 3;41(5):649–58. doi: 10.1021/acs.organomet.2c00015 (PMC8925021; doi:10.1021/acs.organomet.2c00015)
Supplement: Supplementary file 1 — om2c00015_si_001.pdf [file om2c00015_si_001.pdf]

SUPPORTING INFORMATION for

Synthesis of benzofused *O*- and *N*-heterocycles through cascade carbopalladation/cross-alkylation of alkynes involving the C–C cleavage of cyclobutanols

Marta Pérez-Gómez,† Piedad Herrera-Ramírez,† Delia Bautista,‡ Isabel Saura-Llamas† and José-Antonio García-López*,†

†Grupo de Química Organometálica, Departamento de Química Inorgánica, Facultad de Química, Universidad de Murcia, E-30100 Murcia, Spain. ‡ACTI, Universidad de Murcia, E-30100 Murcia, Spain.

E-mail: joangalo@um.es

- Synthetic procedures and characterization data of the starting materials S1-9
- Optimization table for the cascade reaction S10
- References S11
- ^{1}H- and ^{13}C-NMR spectra of the compounds **1** and **3** S12–37
- NOESY-NMR spectra of the compounds **3d** and **3u** S38
- Representative example of the evolution of ^{1}H-NMR for compounds **3** S39

Synthetic procedures of the starting materials and characterization data

General procedures and characterization for the substrates

The synthesis of the substrates **1** required the use of 2-bromophenol derivatives, propargyl alcohol, triphenylphosphine, diisopropylazodicarboxylate, 2-bromoaniline derivatives, 4-methylbenzene-1-sulfonyl chloride, (3-chloroprop-1-yn-1-yl)benzene, 3-phenylpropiolic acid, which were purchased from commercial sources.

The corresponding cyclobutanols derivatives **2** were synthesized following the procedures previously reported in the literature.[1]

Representative procedure B:

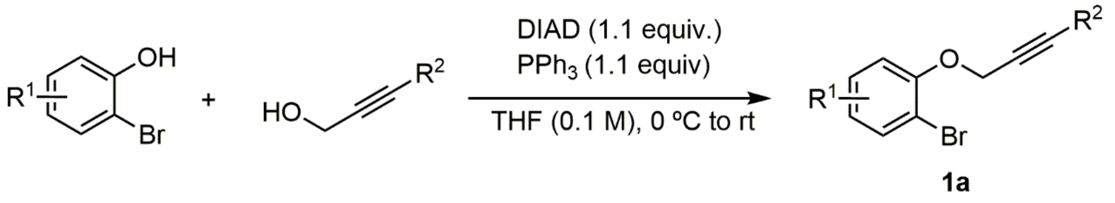

The substrate **1a** was synthesized following an analogous procedure described in the literature for similar alkynyl derivatives.[2]

Diisopropylazodicarboxylate (DIAD, 1mL, 5.1 mmol, 1.1 equiv) was added dropwise to a mixture of 2-bromophenol (534 μL, 4.6 mmol, 1 equiv.), propargyl alcohol (608 μL, 5.1 mmol, 1.1 equiv.) and triphenylphosphine (1300 mg, 5.1 mmol, 1.1 equiv.) in dry THF (mL) at 0 ºC. The reaction was warmed to rt and stirred overnight. The crude was concentrated under reduced pressure and was purified by flash chromatography using EtOAc in Hexane to afford the desired product **1a** as a white solid (1000 mg, 3.5 mmol, 77 %).

Representative procedure C:

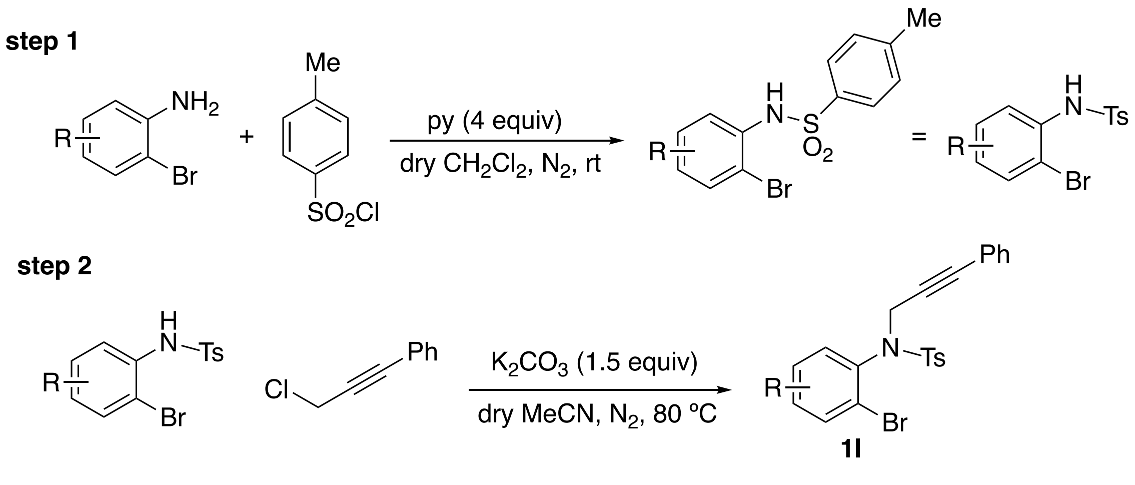

Step 1: The synthesis of the compound *N*-(2-bromophenyl)-4-methylbenzenesulfonamide has been carried out following the procedure described in the literature.[3]

To a solution of 2-bromoaniline (0.95 g, 5.5 mmol) and pyridine (1.5 mL) in dry CH_2Cl_2, 4 methylbenzene-1-sulfonyl chloride (0.95 g, 5.0 mmol) was added under N_2 atmosphere. The mixture was stirred at room temperature overnight. The mixture was diluted with CH_2Cl_2 (25 mL), washed with 10 % aq. HCl (2 x 40 mL) and 0.5 M aq. $NaHCO_3$ (2 x 40 mL). The organic layer was dried over $MgSO_4$, filtered and concentrated

to get *N*-(2-bromophenyl)-4-methylbenzenesulfonamide (1.3 g, 4.0 mmol, 83 %) which was used in the next step without further purification.

Step 2: Synthesis of *N*-(2-bromophenyl)-4-methyl-*N*-(3-phenylprop-2-yn-1-yl)benzenesulfonamide **1l**. To a solution of *N*-(2-bromophenyl)-4-methylbenzenesulfonamide (260 mg, 0.80 mmol) and K_2CO_3 (160 mg, 1.20 mmol) in CH_3CN (5 mL), (3-chloroprop-1-yn-1-yl)benzene (154 µL, 1.12 mmol) was added. The reaction mixture was refluxed for 16 h in a Carius tube under nitrogen atmosphere. The mixture was diluted with EtOAc (30 mL) and washed with water (2 x 30 mL). The organic phase was dried over anhydrous $MgSO_4$ and filtered. The solvent was removed under vacuum and the crude was purified by flash column chromatography to afford the compound **1** as a white solid (185 mg, 0.42 mmol, 52 %).

Representative procedure D: The synthesis of the *N*-(2-bromophenyl)-*N*-methyl-3-phenylpropiolamide **1m** has been carried out following the procedure described in the literature.[4]

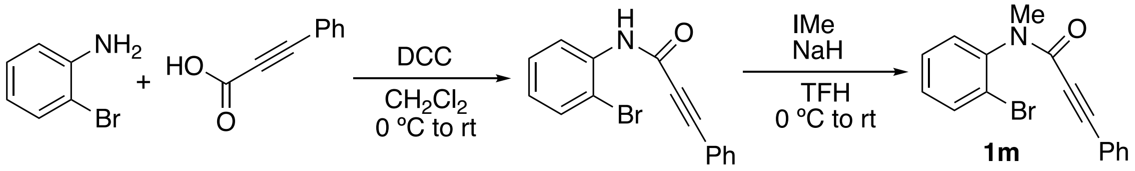

Step 1: Dicyclohexyl carbodimide (DCC, 1056 mg, 5.2 mmol, 1.3 equiv.) was added to a stirred solution of 2-bromoaniline (0.43 mL, 4 mmol, 1 equiv.) and 3-phenylpropiolic acid (758 mg, 5.2 mmol, 1.3 equiv.) in CH_2Cl_2 (5 mL) at 0 ºC. The reaction was then warmed to room temperature and stirred for 16 h. The mixture was quenched with aq. NH_4Cl (30 mL) and washed with EtOAc (2 x 30 mL). The combined organic layers were washed with brine, dried over $MgSO_4$ and filtered. The solvent was removed under vacuum and the resulting crude was used in the next step without further purification.

Step 2: NaH (60% in mineral oil, 232 mg, 5.8 mmol, 2.0 equiv.) was added in portions to a solution of the crude amide obtained in the first step 1 in dry THF (15 mL) under N_2 atmosphere in a Schleck tube at 0 °C. After stirring for 30 min at 0°C, MeI (8.7 mmol, 0.5 mL, 3.0 equiv.) was added. The reaction mixture was further stirred overnight. The reaction was quenched with water (50 mL) and the residue was extracted with EtOAc (2 x 50 mL). The combined organic layers were washed with brine, dried over $MgSO_4$, filtered and concentrated in vacuum. The crude was purified by column chromatography (silica-gel, hexane/EtOAc, gradient from 0 to 10% EtOAc) to afford the amide **1m** as a dense yellow oil, (595 mg, 1.90 mmol, 48 % yield).

Representative Procedure E: The synthesis of 1-iodo-2-(((3-phenylprop-2-yn-1-yl)oxy)methyl)benzene **1k** has been carried out following the procedure described in the literature.[5]

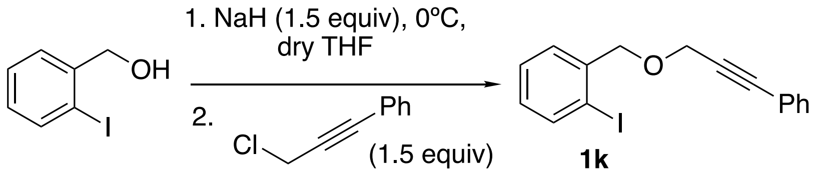

To a suspension of NaH (60% dispersión in mineral oil) (36 mg, 0.90 mmol, 1.5 equiv.) in anhydrous THF (5 mL) was carefully added a solution of (2-iodophenyl)methanol (140 mg, 0.60 mmol) in dry THF (5 mL) under N_2 atmosphere at 0 ºC. The reaction mixture was stirred at room temperature during 30 minutes. (3-chloroprop-1-yn-1-yl)benzene (124 µL, 0.90 mmol, 1.5 equiv.) was then added to the reaction and the resulting was further stirred at room temperature overnight. The reaction was quenched with H_2O and extracted with EtOAc (15 mL x 2). The collected organic phase was washed with brine (2 x 10 mL), dried over $MgSO_4$, filtered and concentrated under vacuum. The crude was purified by column chromatography (silica-gel, petroleum hexane/EtOAc, gradient from 0 to 5% EtOAc) to afford the compound **1k** as a light-yellow solid, (134 mg, 0.38 mmol, 64 % yield).

Characterization data for the starting materials (1a-1p).

Compound 1-bromo-2-((3-phenylprop-2-yn-1-yl)oxy)benzene (1a) was obtained

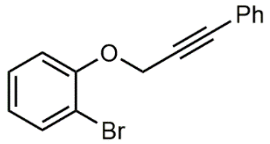

from 2-bromophenol (534 µL, 4.62 mmol) and 3-phenylprop-2-yn-1-ol (608 µL, 5.08 mmol) following the procedure **B** as a white solid (1g, 3.50 mmol, 76 %). ^{1}H-NMR (600 MHz, $CDCl_3$): δ 7.54 (dd, J = 7.9, 1.6 Hz, 1 H), 7.44 – 7.38 (m, 2 H), 7.31 – 7.22 (m, 4 H), 7.12 (dd, J = 8.3, 1.4 Hz, 1 H), 6.85 (td, J = 7.6, 1.4 Hz, 1 H), 4.96 (s, 2 H). ^{13}C-NMR (151 MHz, $CDCl_3$): δ 154.2 (s, C_q), 133.5 (s, CH), 131.7 (s, CH), 128.7 (s, CH),128.3 (s, CH), 128.2 (s, CH),122.7 (s, CH), 122.1 (s, C_q), 114.5 (s, CH), 112.5 (s, C_q), 87.7 (s, C_q), 83.4 (s, C_q), 57.7 (s, CH_2).This compound has been previously reported in the literature.[2,6]

Compound 1-iodo-2-((3-phenylprop-2-yn-1-yl)oxy)benzene (1b) was obtained from

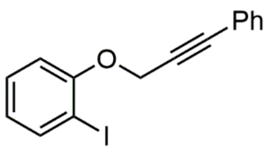

2-iodophenol (154 µL, 1.36 mmol) and 3-phenylprop-2-yn-1-ol (133 µL, 1.50 mmol) following the procedure **B** as a white solid (350 mg, 1.05 mmol, 77 %). ^{1}H-NMR (400 MHz, $CDCl_3$): δ 7.79 (dt, J = 7.9, 1.1 Hz, 1 H), 7.42 (dd, J = 7.7, 2.1 Hz, 2 H), 7.36 – 7.26 (m, 4 H), 7.09 (dd, J = 8.3, 1.3 Hz, 1 H), 6.75 (td, J = 7.6, 1.3 Hz, 1 H), 4.98 (s, 2 H). ^{13}C-NMR (101 MHz, $CDCl_3$): δ 156.6 (s, C_q), 139.6 (s, CH), 131.8 (s, CH), 129.4 (s, CH), 128.7 (s, CH), 128.3 (s, CH), 123.3 (s, CH), 122.1 (s, C_q), 113.3 (s, CH), 87.7 (s, C_q), 86.7 (s, C_q), 83.4 (s, C_q), 57.8 (s, CH_2). This compound has been previously reported in the literature.[6,7]

Compound 2-bromo-4-methoxy-1-((3-phenylprop-2-yn-1-yl)oxy)benzene (1c) was

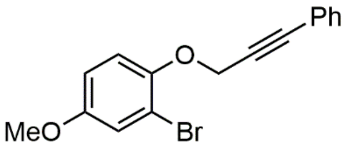

obtained from 2-bromo-4-methoxyphenol (325 mg, 1.60 mmol) and 3-phenylprop-2-yn-1-ol (156 µL, 1.76 mmol) following the procedure **B** as a white solid (324 mg, 1.02 mmol, 64 %). IR (cm^{-1}): $\bar{\nu}$ 2215 (s), 1638 (s), 1487 (s), 1369 (s), 1313 (m), 1209 (m), 1128 (m), 1031 (m), 998 (m), 764 (s), 727 (s), 691 (s). ^{1}H-NMR (300 MHz, $CDCl_3$): δ 7.45 – 7.39 (m, 2 H), 7.34 – 7.23 (m, 3 H), 7.15 – 7.05 (m, 2 H), 6.83 (dd, J = 9.0, 3.0 Hz, 1 H), 4.92 (s, 2 H), 3.76 (s, 3 H). ^{13}C-NMR (151 MHz, $CDCl_3$): δ 155.0 (s, C_q), 148.6 (s, C_q), 131.8 (s, CH), 128.7 (s, CH), 128.3 (s, CH), 122.3 (s, C_q), 118.8 (s, CH), 116.8 (s, CH), 113.7 (s, CH), 113.5 (s, C_q), 87.6 (s, C_q), 83.8 (s, C_q), 58.9 (s, CH_2), 55.9 (s, CH_3). HR-MS (+ESI) *m/z* calculated for $C_{16}H_{13}BrNaO_2$ $[M+Na]^+$ 338.9991, found 338.9975.

Compound 2-bromo-4-methyl-1-((3-phenylprop-2-yn-1-yl)oxy)benzene (1d) was obtained from 2-bromo-4-methylphenol (162 µL, 1.34 mmol) and 3-phenylprop-2-yn-1-ol

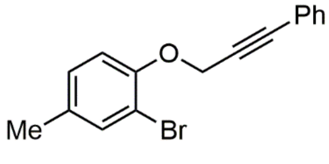

(133 µL, 1.47 mmol) following the procedure **B** as light yellow solid (296 mg, 0.99 mmol, 74 %). IR (cm^{-1}): $\bar{\nu}$ 2237 (m), 1774 (m), 1601 (m), 1488 (s), 1442 (m), 1363, 1228 (s), 1052 (s), 1015 (s), 996 (s), 962 (s), 798 (s), 758 (s). ^{1}H-NMR (300 MHz, $CDCl_3$): δ 7.46 – 7.39 (m, 2 H), 7.37 (dt, J = 1.8, 0.6 Hz, 1 H), 7.33 – 7.25 (m, 3 H), 7.10 – 6.97 (m, 2 H), 4.95 (s, 2 H), 2.27 (s, 3 H). ^{13}C-NMR (75.45 MHz, $CDCl_3$): δ 152.1 (s, C_q), 133.7 (s, CH), 132.5 (s, C_q), 131.7 (s, CH), 128.7 (s, CH), 128.6 (s, CH), 128.2 (s, CH), 122.1 (s, C_q), 114.6 (s, CH), 112.2 (s, C_q), 87.5 (s, C_q), 83.6 (s, C_q), 57.9 (s, CH_2), 20.1 (s, CH_3). This compound has been previously reported in the literature.[7]

Compound 4-fluoro-2-iodo-1-((3-phenylprop-2-yn-1-yl)oxy)benzene (1e) was

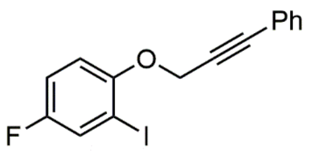

obtained from 4-fluoro-2-iodophenol (380 mg, 1.59 mmol) and 3-phenylprop-2-yn-1-ol (156 µL, 1.76 mmol) following the procedure **B** as a white solid (312 mg, 0.88 mmol, 55 %). IR (cm^{-1}): $\bar{\nu}$ 2360 (m), 2234 (m), 1588 (s), 1475 (s), 1367 (m), 1290 (m), 1235 (m), 1037 (s), 855 (s), 794 (s), 758 (s), 726 (s), 689 (s). ^{1}H-NMR (400 MHz, $CDCl_3$): *δ* 7.53 (ddd, *J* = 7.6, 2.4, 0.9 Hz, 1 H), 7.47 – 7.41 (m, 2 H), 7.37 – 7.29 (m, 3 H), 7.09 – 7.00 (m, 2 H), 4.94 (s, 2 H). ^{13}C-NMR (101 MHz, $CDCl_3$): *δ* 157.2 (d, J_{CF} = 245.0 Hz, C_q), 153.1 (d, J_{CF} = 2.5 Hz, C_q), 131.7 (s, CH), 128.8 (s, CH), 128.3 (s, CH), 126.2 (d, *J* = 25 Hz, CH), 121.9 (s, C_q), 115.5 (d, *J* = 22.7 Hz, CH), 113.9 (d, *J* = 8.2 Hz, CH), 87.8 (s, C_q), 86.4 (d, J_{CF} = 8.5 Hz, C_q), 83.2 (s, C_q), 58.5 (s, CH_2).
^{19}F-NMR (376.5 MHz, $CDCl_3$): *δ* -120.9 (s). HR-MS (+ESI) *m/z* calculated. for $C_{15}H_{10}FINaO$ $[M+Na]^+$ 374.9653 found 374.9644. This compound has been previously reported in the literature.[6]

Compound 2-bromo-1-((3-phenylprop-2-yn-1-yl)oxy)-4-(trifluoromethyl)benzene

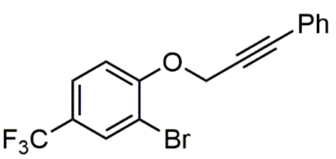

(1f) was obtained from 2-bromo-4-(trifluoromethyl)phenol (143 µL, 1.04 mmol) and 3-phenylprop-2-yn-1-ol (101 µL, 1.14 mmol) following the procedure **B** as a light yellow solid (226 mg, 0.63 mmol, 61 %). IR (cm^{-1}): $\bar{\nu}$ 2364 (m), 2243 (m), 1607 (s), 1494 (s), 1444 (s), 1320 (s), 1265 (s), 1073 (s), 996 (s), 762 (s). ^{1}H-NMR (300 MHz, $CDCl_3$): *δ* 7.83 – 7.81 (m, 1 H), 7.57 (ddq, *J* = 8.6, 2.2, 0.8 Hz, 1 H), 7.47 – 7.38 (m, 2 H), 7.37 – 7.28 (m, 3 H), 7.22 (dd, *J* = 8.7, 0.8 Hz, 1 H), 5.05 (s, 2 H). ^{13}C-NMR (75.45 MHz, $CDCl_3$): *δ* 156.7 (s, C_q), 131.8 (s, CH), 130.8 (q, *J* = 3.3 Hz, CH), 129.0 (s, CH), 128.4 (s, CH), 125.7 (q, *J* = 3.4 Hz, CH), 125.2 – 124.0 (m, C_q), 121.7 (s, C_q), 121.6 (s, C_q), 113.4 (s, CH), 112.5 (s, C_q), 88.5 (s, C_q), 82.3 (s, C_q), 57.8 (s, CH_2). ^{19}F-NMR (282.4 MHz, $CDCl_3$): *δ* -61.7 (s). HR-MS (+ESI) *m/z* calculated for $C_{16}H_{10}BrF_3NaO$ $[M+Na]^+$ 376.9759, found 376.9762.

Compound 2-bromo-3-((3-phenylprop-2-yn-1-yl)oxy)benzene (1g) was obtained

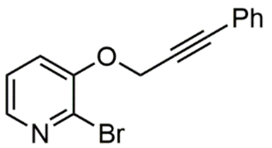

from 2-bromopyridin-3-ol (277 mg, 1.16 mmol) and 3-phenylprop-2-yn-1-ol (113 µL, 1.27 mmol) following the procedure **B** as a light yellow solid (200 mg, 0.69 mmol, 59 %). IR (cm^{-1}): $\bar{\nu}$ 2372 (m), 1563 (s), 1489 (s), 1412 (s), 1371 (s), 1280 (s), 1203 (s), 1028 (s), 994 (s), 758 (s). ^{1}H NMR (300 MHz, $CDCl_3$): *δ* 8.05 (dd, *J* = 4.7, 1.6 Hz, 1 H), 7.48 – 7.37 (m, 3 H), 7.39 – 7.27 (m, 3 H), 7.31 – 7.20 (m, 1 H), 5.04 (s, 2 H). ^{13}C NMR (75.45 MHz, $CDCl_3$): *δ* 151.3 (s, C_q), 142.1 (s, CH), 133.2 (s, C_q), 131.8 (s, CH) , 129.0 (s, CH), 128.4

(s, CH) , 123.2 (s, CH) , 121.7 (s, C_q), 121.1 (s, CH) , 88.7 (s, C_q), 82.2 (s, C_q), 57.7 (s, CH_2). HR-MS (+APCI) *m/z* calculated for $C_{14}H_{11}BrNO$ $[M+H]^+$ 288.0018, found 288.0019.

Compound (3-(2-bromophenoxy)prop-1-yn-1-yl)trimethylsilane (1h) was obtained

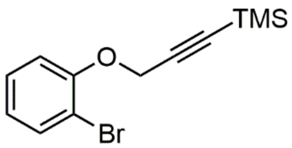

from 2-bromophenol (185 µL, 1.73 mmol) and 3-(trimethylsilyl)prop-2-yn-1-ol (383 µL, 1.91 mmol) following the procedure **B** as a white solid (306 mg, 1.08 mmol, 62 %). IR (cm^{-1}): $\bar{\nu}$ 2364 (m), 2181 (m), 1574 (m), 1475 (s), 1248 (s), 1227 (s), 1054 (s), 1028 (s), 985 (s), 843 (s), 750 (s). ^{1}H-NMR (300 MHz, $CDCl_3$): δ 7.55 (dd, *J* = 7.9, 1.6 Hz, 1 H), 7.27 (ddd, *J* = 8.3, 7.4, 1.6 Hz, 1 H), 7.09 (dd, *J* = 8.3, 1.4 Hz, 1 H), 6.88 (ddd, *J* = 7.9, 7.4, 1.5 Hz, 1 H), 4.76 (s, 2 H), 0.17 (s, 9 H). ^{13}C-NMR (75.45 MHz, $CDCl_3$): δ 154.2 (s, C_q), 133.4 (s, CH), 128.2 (s, CH), 122.7 (s, CH), 114.7 (s, CH), 112.5 (s, C_q), 99.5 (s, C_q), 93.5 (s, C_q), 57.8 (s, CH_2), -0.37 (s, CH_3). This compound has been previously reported in the literature.[8]

Compound (1-bromo-2-(but-2-yn-1-yloxy)benzene (1i) was obtained from 2-

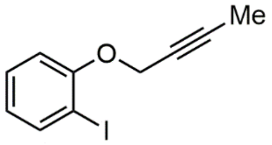

iodophenol (103 µL, 0.91 mmol) and 1-bromobut-2-yne (88 µL, 1.00 mmol) following the procedure **C** as a white solid (135 mg, 0.50 mmol, 55 %).

^{1}H NMR (300 MHz, $CDCl_3$): δ 7.76 (dd, *J* = 7.8, 1.6 Hz, 1 H), 7.28 (ddd, *J* = 8.3, 7.3, 1.6 Hz, 1 H), 6.96 (dd, *J* = 8.3, 1.4 Hz, 1 H), 6.71 (td, *J* = 7.6, 1.4 Hz, 1 H), 4.69 (q, *J* = 2.3 Hz, 2 H), 1.83 (t, *J* = 2.4 Hz, 3 H). This compound has been previously reported in the literature.[7]

Compound *N*-(2-bromophenyl)4-methyl-*N*-(3-phenylprop-2-yn-1-yl)benzenesulfonamide (1j) was obtained from *N*-(2-bromophenyl)-4-

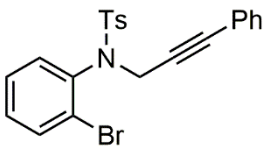

methylbenzenesulfoamide (260 mg, 0.80 mmol) and (3-chloroprop-1-yn-1-yl)benzene (154 µL, 1.12 mmol) following the procedure **C** as a light yellow solid (185 mg, 0.42 mmol, 52 %). IR (cm^{-1}): $\bar{\nu}$ 2361 (m), 2334 (m), 1597 (s), 1470 (s), 1346 (s), 1163 (s), 1088 (s), 892 (s), 751 (s). ^{1}H-NMR (300 MHz, $CDCl_3$): δ 7.79 – 7.74 (m, 2 H), 7.71 – 7.63 (m, 1 H), 7.35 – 7.14 (m, 10 H), 4.99 (d, *J* = 18.2 Hz, 1 H), 4.36 (d, *J* = 18.2 Hz, 1 H), 2.41 (s, 3 H). ^{13}C-NMR (75.45 MHz, $CDCl_3$): δ 143.8 (s, C_q), 137.6 (s, C_q), 136.9 (s, C_q), 133.8 (s, CH), 131.9 (s, CH), 131.4 (s, CH), 130.3 (s, CH), 129.5 (s, CH), 128.4 (s, CH), 128.2 (s, CH), 128.1 (s, CH), 127.8 (s, CH), 126.0 (s, C_q), 122.3 (s, C_q), 85.6 (s, C_q), 83.1 (s, C_q), 41.3 (s, CH_2), 21.6 (s, CH_3). HR-MS (+ESI) *m/z* calculated. for $C_{22}H_{19}BrNO_2S$ $[M+H]^+$ 440.0314, found 440.0321. This compound has been previously reported in the literature.[9]

Compound 1-iodo-2-(((3-phenylprop-2-yn-1-yl)oxy)methyl)benzene (1k) was

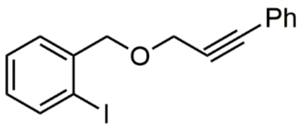

obtained from (2-iodophenyl)methanol (164 mg, 0.60 mmol) and (3-chloroprop-1-yn-1-yl)benzene (124 µL, 0.90 mmol) following the procedure **C** as a light yellow solid (134 mg, 0.39 mmol, 64 %). IR (cm^{-1}): $\bar{\nu}$ 2364, 2227, 1690, 1561, 1489, 1440, 1349, 1278, 1075, 1008, 747, 689. ^{1}H-NMR (400 MHz, $CDCl_3$): δ 7.83 (dd, J = 7.8, 1.2 Hz, 1 H), 7.51 – 7.43 (m, 3 H), 7.34 (td, J = 7.5, 1.1 Hz, 1 H), 7.32 – 7.28 (m, 3 H), 6.98 (tdd, J = 7.9, 1.8, 0.5 Hz, 1 H), 4.67 (s, 2 H), 4.48 (s, 2 H). ^{13}C-NMR (75.45 MHz, $CDCl_3$): δ 139.9 (s, C_q), 139.2 (s, CH), 131.7 (s, CH), 129.4 (s, CH), 129.1 (s, CH), 128.5 (s, CH), 128.3 (s, CH), 128.1 (s, CH), 122.5 (s, C_q), 98.1 (s, C_q), 86.7 (s, C_q), 84.8 (s, C_q), 75.4 (s, CH_2), 58.5 (s, CH_2). This compound has been previously reported in the literature.[11]

Compound *N*-(2-bromobenzyl)-4-methyl-*N*-(3-phenylprop-2-yn-1-yl)benzenesulfonamide (1l) was obtained from *N*-(2-bromobenzyl)-4-

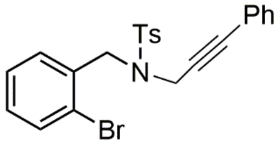

methylbenzenesulfoamide (152 mg, 0.45 mmol) and (3-chloroprop-1-yn-1-yl)benzene (68 µL, 0.49 mmol) following the procedure **C** as a light yellow solid (134 mg, 0.33 mmol, 73 %). IR (cm^{-1}): $\bar{\nu}$ 2332 (m), 1599 (m), 1486 (m), 1431 (m), 1320 (s), 1254 (m), 1089 (s), 1024 (s), 904 (s), 807 (m), 756 (s), 689 (s). ^{1}H-NMR (300 MHz, $CDCl_3$): δ 7.85 – 7.83 (m, 2 H), 7.61 (dd, J = 7.7, 1.7 Hz, 1 H), 7.55 (dd, J = 8.0, 1.3 Hz, 1 H), 7.35 (td, J = 7.5, 1.3 Hz, 1 H), 7.31 – 7.21 (m, 5 H), 7.17 (td, J = 7.7, 1.7 Hz, 1 H), 7.12 – 7.03 (m, 2 H), 4.59 (s, 2 H), 4.21 (s, 2 H), 2.32 (s, 3 H). ^{13}C-NMR (75.45 MHz, $CDCl_3$): δ 143.7 (s, C_q), 135.8 (s, C_q), 134.6 (s, C_q), 133.0 (s, CH), 131.5 (s, CH), 130.1 (s, CH), 129.6 (s, CH), 129.4 (s, CH), 128.4 (s, CH), 128.1 (s, CH), 127.84 (s, CH),127.80 (s, CH), 123.7 (s, C_q), 122.1 (s, C_q), 86.1 (s, C_q), 81.5 (s, C_q), 50.0 (s, CH_2), 37.4 (s, CH_2), 21.4 (s, CH_3). HR-MS (+ESI) *m/z* calculated. for $C_{23}H_{21}BrNO_2S$ $[M+H]^+$ 454.0471, found 454.0474.

Compound *N*-(2-bromophenyl)-*N*-methyl-3-phenylpropiolamide (1m) was obtained

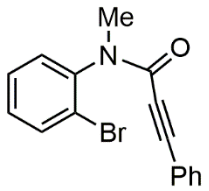

from 2-bromoaniline (0.43 mL, 4.00 mmol) and 3-phenylpropiolic acid (758 mg, 5.20 mmol) following the procedure **D** as a yellow oil (595 mg, 1.90 mmol, 48 %). IR (cm^{-1}): $\bar{\nu}$ 2925 (m), 2219 (m), 1642 (CO, s), 1478 (m), 1130 (m), 1028 (m), 758 (s), 728 (s), 687 (m), 651 (m). ^{1}H-NMR (300 MHz, $CDCl_3$): δ 7.72 (dt, J = 8.2, 0.9 Hz, 1 H), 7.46 – 7.37 (m, 2 H), 7.34 – 7.26 (m, 2 H), 7.26 – 7.17 (m, 2 H), 7.13 – 7.03 (m, 2 H), 3.32 (s, 3 H). ^{13}C-NMR (75.45 MHz, $CDCl_3$): δ 154.4 (s, C_q), 142.1 (s, C_q), 133.5 (s, CH), 132.5 (s, CH), 130.6 (s, CH), 130.0 (s, CH), 129.9 (s, CH), 128.5 (s, CH), 128.3 (s, CH), 123. 9 (s, C_q), 120.2 (s, C_q),

90.4 (s, C_q), 82.1 (s, C_q), 35.1 (s, CH_3). HR-MS (+ESI) *m/z* calculated. for $C_{16}H_{13}BrNO$ $[M+H]^+$ 314.0175, found 314.0185. This compound has been previously reported in the literature.[10]

Compound *N*-(2-bromo-4-methylphenyl)-*N*-methyl-3-phenylpropiolamide (1n) was

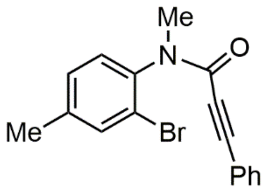

obtained from 2-bromo-4-methylaniline (334 mg, 1.80 mmol) and 3-phenylpropioloyl chloride (295 mg, 1.80 mmol) following the procedure **D** as a yellow oil (272 mg, 0.83 mmol, 46 %). IR (cm^{-1}): $\bar{\nu}$ 2217 (m), 1637 (CO, s), 1491 (m), 1364 (m), 1313 (m), 1124 (m), 1066 (m), 825 (s), 761 (s), 693 (s).

^{1}H-NMR (400 MHz, $CDCl_3$): δ 7.53 (d, J = 1.1 Hz, 1 H), 7.34 – 7.16 (m, 5 H), 7.14 – 7.07 (m, 2 H), 3.29 (s, 3 H), 2.40 (s, 3 H). ^{13}C-NMR (101 MHz, $CDCl_3$): δ 154.2 (s, C_q), 140.4 (s, C_q), 139.4 (s, C_q), 134.0 (s, CH), 132.4 (s, CH), 130.2 (s, CH), 129.8 (s, CH), 129.1 (s, CH), 128.2 (s, CH), 123.3 (s, C_q), 120.3 (s, C_q), 90.2 (s, C_q), 82.2 (s, C_q), 35.2 (s, CH_3), 20.8 (s, CH_3). HR-MS (+ESI) *m/z* calculated for $C_{17}H_{14}BrNNaO$ $[M+Na]^+$ 350.0151; found 350.0135.

Compound *N*-(2-bromo-4-chlorophenyl)-N-methyl-3-phenylpropiolamide (1o) was

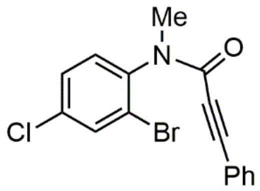

obtained from 2-bromo-4-chloroaniline (372 mg, 1.80 mmol) and 3-phenylpropioloyl chloride (310 mg, 1.80 mmol) following the procedure **D** as a light yellow oil (180 mg, 0.52 mmol, 29 %). IR (cm^{-1}): $\bar{\nu}$ 2215 (m), 1642 (s), 1476 (s), 1378 (s), 1308 (s), 1099 (s), 865 (s), 765 (s). ^{1}H-NMR (300 MHz, $CDCl_3$): δ 7.74 (d, J = 1.8 Hz, 1 H), 7.42 (dd, J = 8.4 , 2.1 Hz, 1 H), 7.37 – 7.33 (m, 2 H), 7.29 – 7.24 (m, 2 H), 7.16 – 7.12 (m, 2 H), 3.29 (s, 3 H). ^{13}C-NMR (75.45 MHz, $CDCl_3$): δ 154.2 (s, C_q), 140.8 (s, C_q), 135.1 (s, C_q), 133.2 (s, CH), 132.5 (s, CH), 131.2 (s, CH), 130.1 (s, CH), 128.7 (s, CH), 128.6 (s, CH), 124.5 (s, C_q), 119.9 (s, C_q), 90.7 (s, C_q), 81.9 (s, C_q), 35.1 (s, CH_3). HR-MS (+ESI) *m/z* calculated. for $C_{16}H_{12}BrClNO$ $[M+H]^+$ 347.9785, found 347.9804.

Optimization of the conditions for the Pd-catalyzed cascade reaction

The compound 1-bromo-2-((3-phenylprop-2-yn-1-yl)oxy)benzene **(1a)** was used as a substrate to check the viability of our proposed cascade process (Table S1). We finally concluded that the best conditions involve the use of [$Pd(PPh_3)_4$] (10 mol%), and Cs_2CO_3 (1.2 equiv.) in toluene at 100 ºC for 16 h, providing the desired cascade product **(3a)** in 70% NMR yield (67 % isolated by colum chromatography) (entry 14).

Table S1: Optimization of reaction conditions

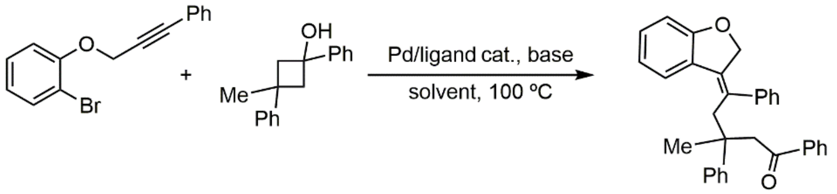

Entry[a]	Pd source (mol%)	Ligand (mol%)	Base (equiv)	Solvent	NMR yield[b] (%)
1	$Pd(OAc)_2$ (10)	PPh_3 (20)	Cs_2CO_3 (1.2 equiv)	Toluene	traces
2	$Pd(dba)_2$ (10)	PPh_3 (20)	Cs_2CO_3 (1.2 equiv)	Toluene	68
3	$Pd(dba)_2$ (10)	Xantphos (20)	Cs_2CO_3 (1.2 equiv)	Toluene	32
4	$Pd(dba)_2$ (10)	PPh_3 (20)	Cs_2CO_3 (1.2 equiv)	THF	62
5	$Pd(dba)_2$ (10)	PPh_3 (20)	Cs_2CO_3 (1.2 equiv)	1,4-Dioxane	traces
6	$Pd(dba)_2$ (10)	PPh_3 (20)	Cs_2CO_3 (1.2 equiv)	1,2-DCE	traces
7	$Pd(dba)_2$ (10)	PCy_3 (20)	Cs_2CO_3 (1.2 equiv)	Toluene	60
8	$Pd(dba)_2$ (10)	JohnPhos (20)	Cs_2CO_3 (1.2 equiv)	Toluene	n.d
9	$Pd(dba)_2$ (10)	PPh_3 (20)	Cs_2CO_3 (1.5 equiv)	Toluene	58
10	$Pd(dba)_2$ (10)	PPh_3 (20)	Cs_2CO_3 (1 equiv)	Toluene	62
11	$Pd(dba)_2$ (10)	PPh_3 (20)	Et_3N (1.2 equiv)	Toluene	-
12	$PdCl_2(PPh_3)_2$ (10)	-	Cs_2CO_3 (1.2 equiv)	Toluene	-
13	$Pd(dba)_2$ (5)	PPh_3 (10)	Cs_2CO_3 (1.2 equiv)	Toluene	10
14	$Pd(PPh_3)_4$ (10)	-	Cs_2CO_3 (1.2 equiv)	Toluene	70(67)[c]

[a] The reactions were carried out using 0.14 mmol of 1-bromo-2-((3-phenylprop-2-yn-1-yl)oxy)benzene (**X**) and 1.2 equiv of 3-methyl,-1,3-diphenylcyclobutan-1-ol in 4 mL of dry solvent under nitrogen atmosphere at 100 ºC in a Carius tube for 16 h. [b] NMR yields using trimethyl benzene-1,3,5-tricarboxylate as standard. [c] Isolated yield.

References

[1] (a) X. Zhao, M. Tian, L. Ji, J. Liu, K. Lu, *Org. Lett.* **2020**, *22*, 863-866; b) B. M. Casey, C. A. Eakin, R. A. Flowers, *Tet. Letters* **2009**, *50*, 1264-1266; c) H. Zeng, P. Pan, J. Chen, H. Gong, C.-J. Li, *Eur. J. Org. Chem.* **2017**, 1070–1073; d) Q. Tian, B. Chen, G. Zhang, *Green Chem.,* **2016**, *18*, 6236-6240.

[2] C. M. Le, P. J. C. Menzies, D. A. Pertone, M. Lautens, *Angew. Chem. In. Ed.* **2015**, *54*, 254-257.

[3] M. Pérez-Gómez, J. A. García-López, *Angew. Chem. Int. Ed.* **2016**, *55*, 46, 14389-14393.

[4] S. Park, K. J. Shin, J. H. Seo, *Synlett*, **2015**, *26*, 2296-2300.

[5] A. Nandakumar, K. Balakrishnan, P. T. Perumal, *Synlett* **2011**, *18*, 2733–2739.

[6] A. Arcadi, F. Blesi, S. Cacchi, G. Fabrizi, A. Goggiamani, F. Marinalli, *J. Org. Chem.* **2013**, *78*, 4490−4498.

[7] K. Paul, S. Jalal, S. Kundal, U. Jana. *J. Org. Chem.* **2016**, *81*, 1164−1174.

[8] J. Barluenga, F. J. Fañanas, R. Sanz, C. Marcos, *Org. Lett.* **2002**, *13*, 2225-2228.

[9] K. Paul, K. Bera, S. Jalal, S. Sarkar, U. Jana. *Org. Lett.* **2014**, 16, 2166-2169.

[10] C.-S. Wang, T. Roisnel, P. H. Dixneuf, J. F. Soulé, *Adv. Synth. Catal.* **2019**, *361*, 445–450.

[11] C. Cheng, X. Zuo, D. Tu, B. Wan, Y. Zhang, *Org. Lett.* **2020**, *22*, 4985−4989.

NMR spectra of non previously reported compounds 1 and 3

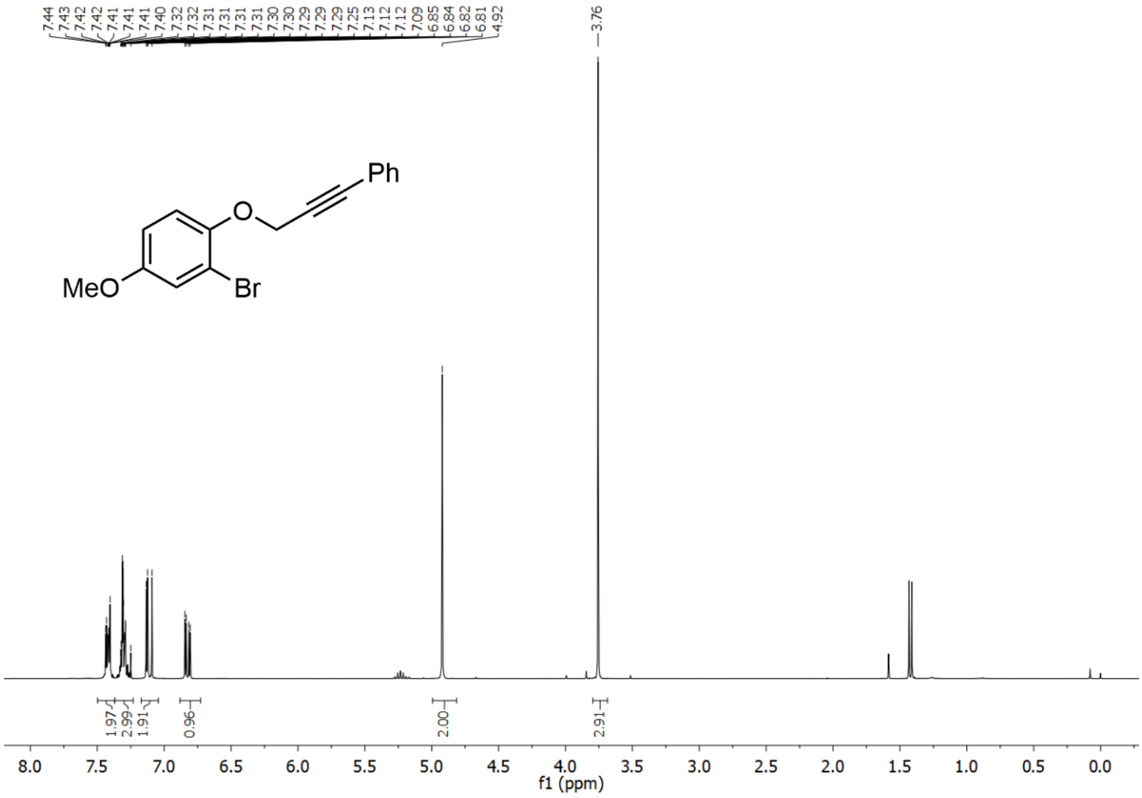

Figure S1. ^{1}H-NMR spectrum (300 MHz, $CDCl_3$) of compound **1c**.

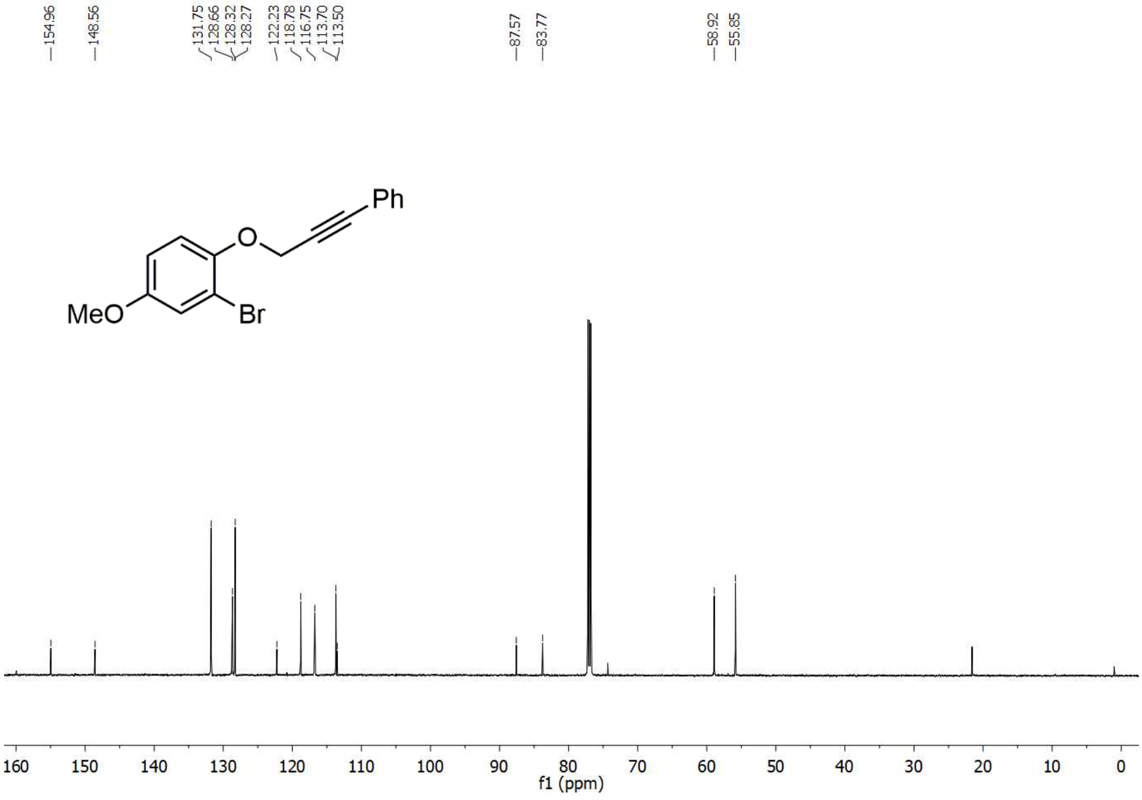

Figure S2. ^{13}C-NMR spectrum (151 MHz, $CDCl_3$) of compound **1c**.

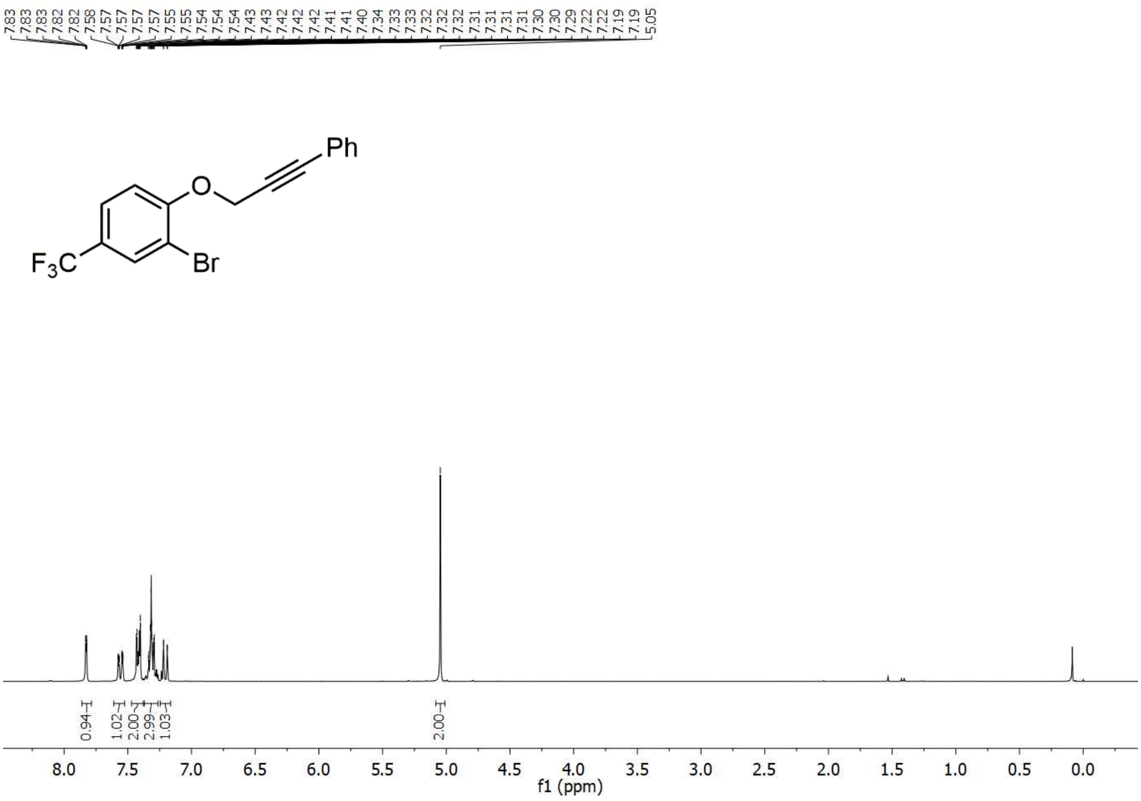

Figure S3. ^{1}H-NMR (300 MHz, $CDCl_3$) spectrum of compound **1f**.

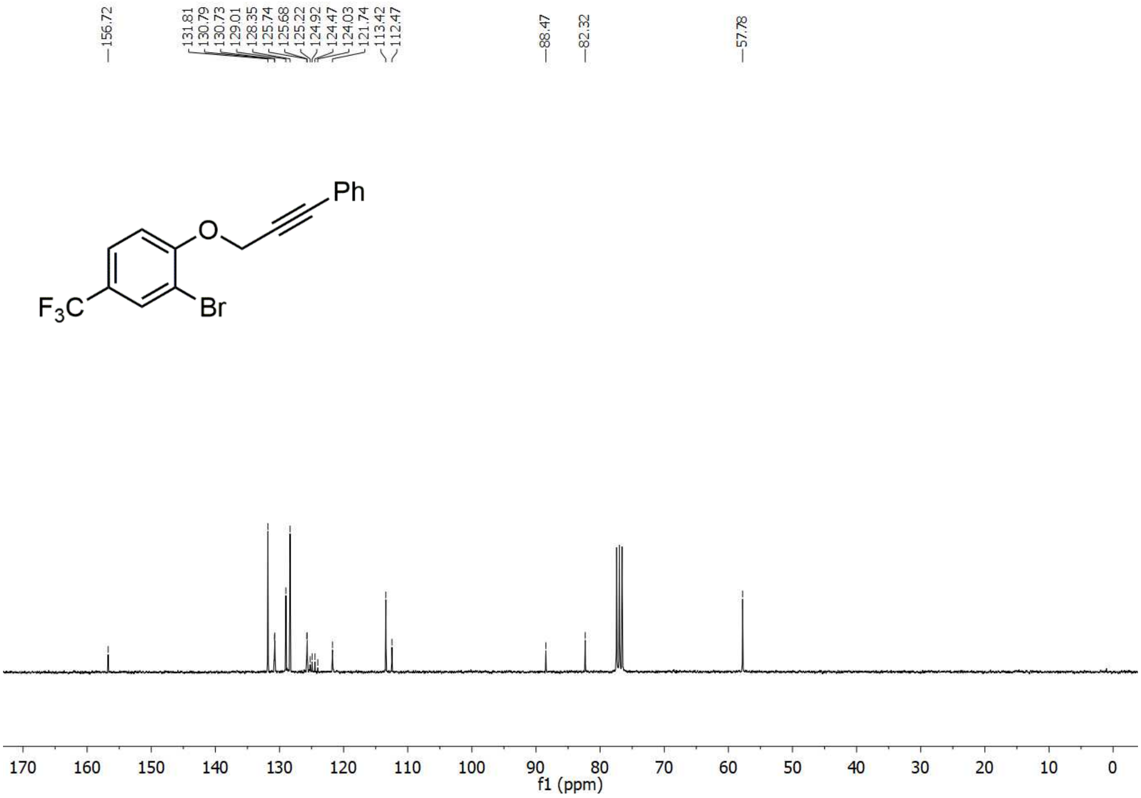

Figure S4. ^{13}C-NMR spectrum (75.45 MHz, $CDCl_3$) of compound **1f**.

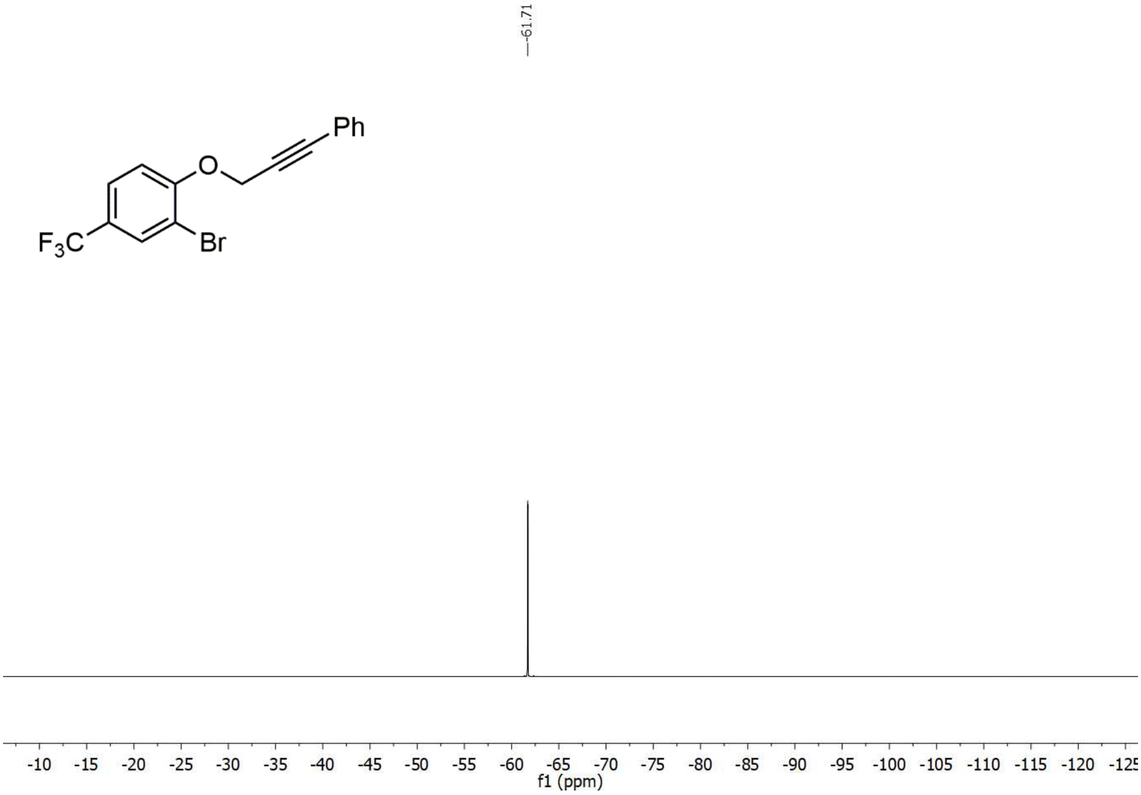

Figure S5. ^{19}F-NMR spectrum (282.4 MHz, $CDCl_3$) of compound **1f**.

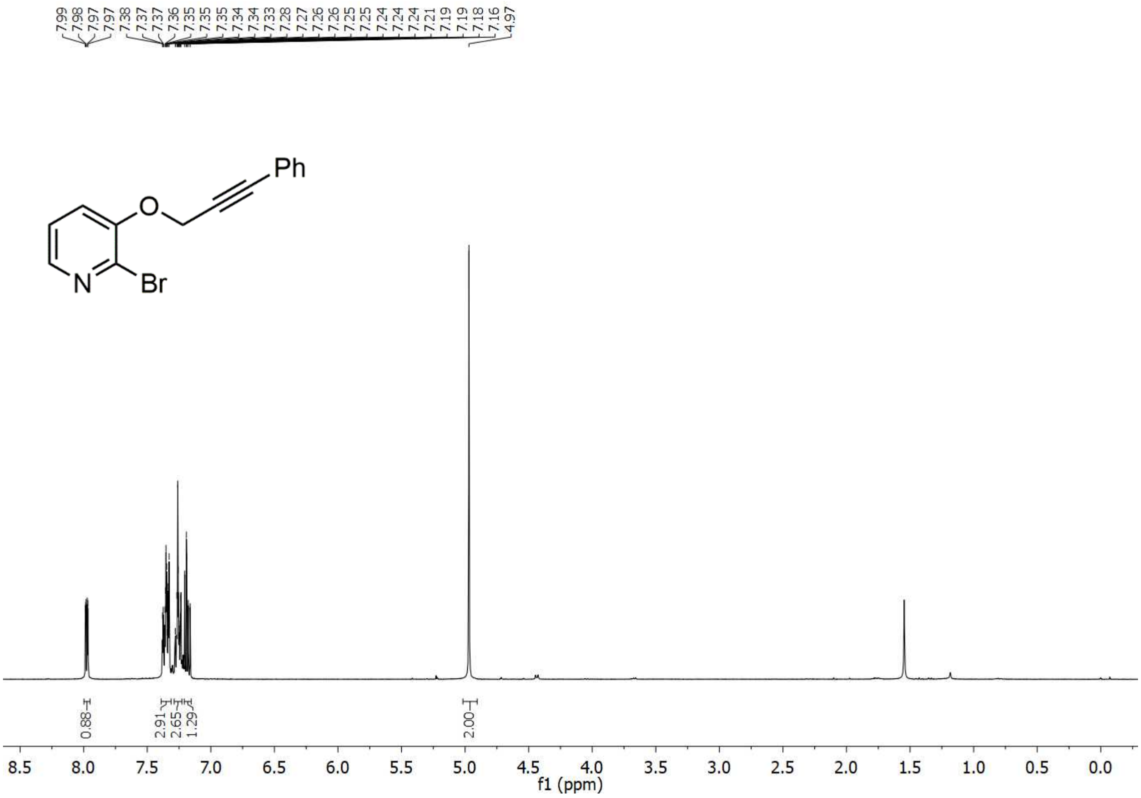

Figure S6. ^{1}H-NMR spectrum (300 MHz, $CDCl_3$) of compound **1g**.

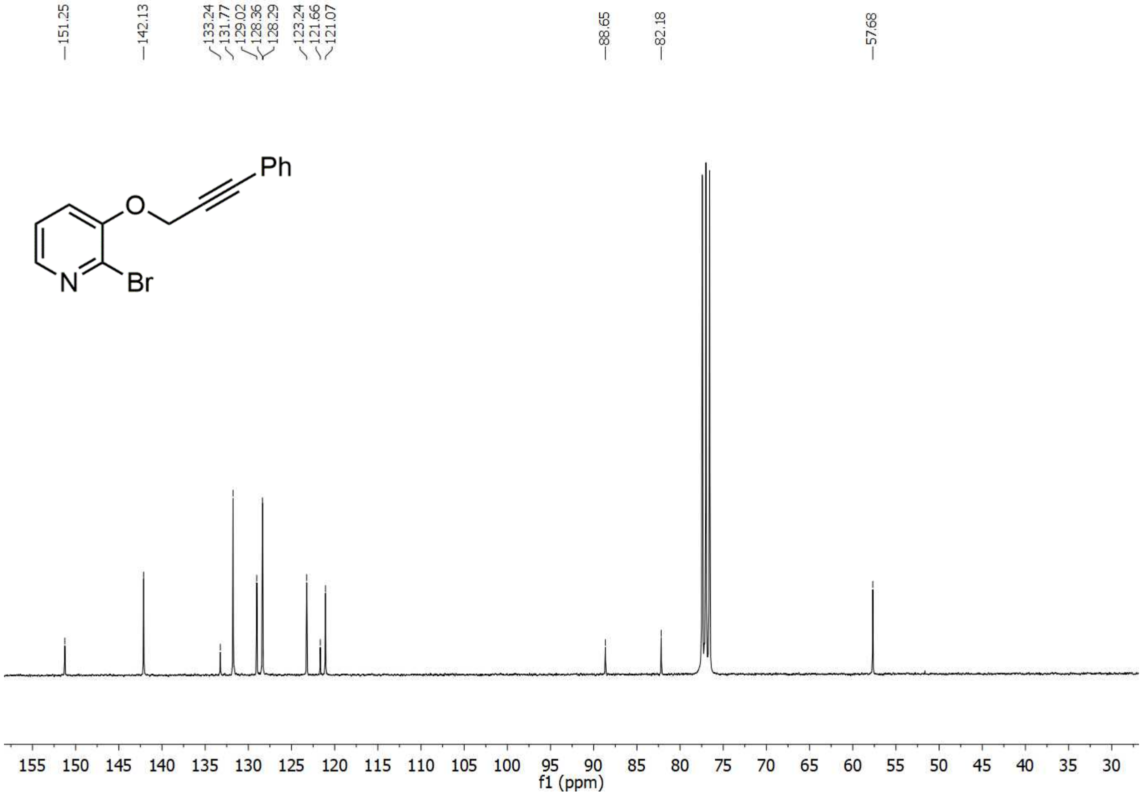

Figure S7. ^{13}C-NMR spectrum (75.45 MHz, $CDCl_3$) of compound **1g**.

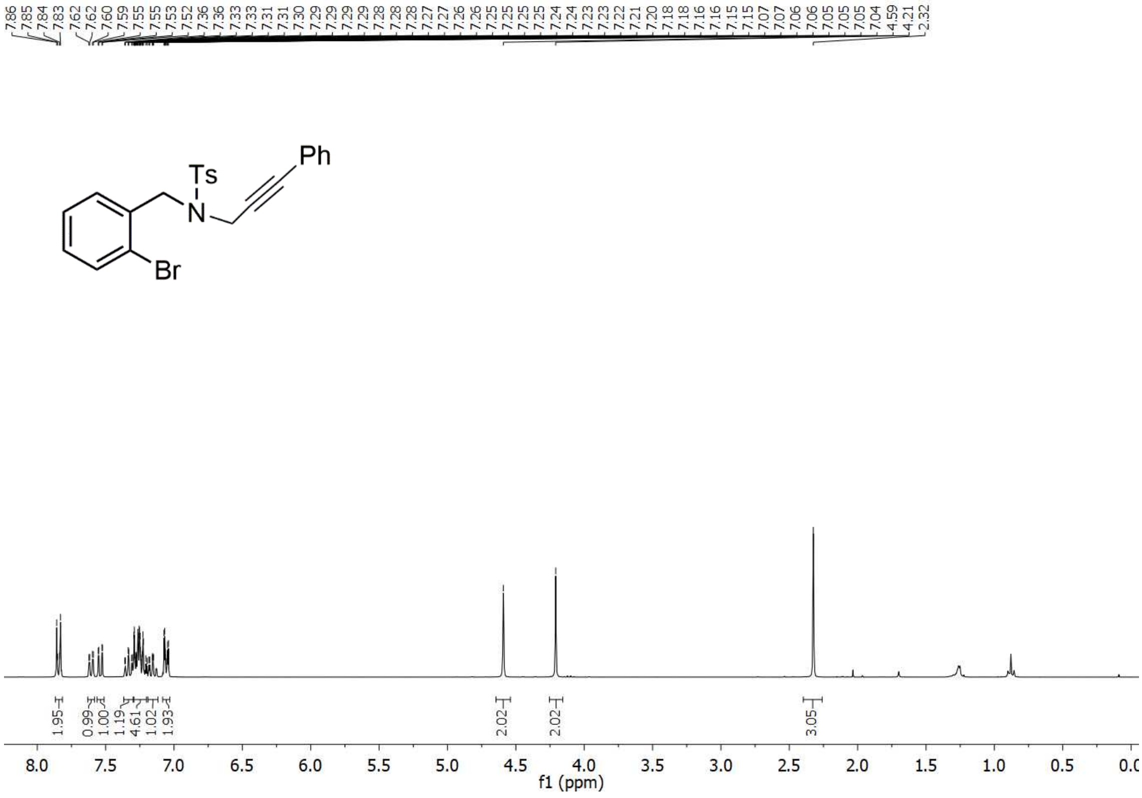

Figure S8. ^{1}H-NMR spectrum (300 MHz, $CDCl_3$) of compound **1l**.

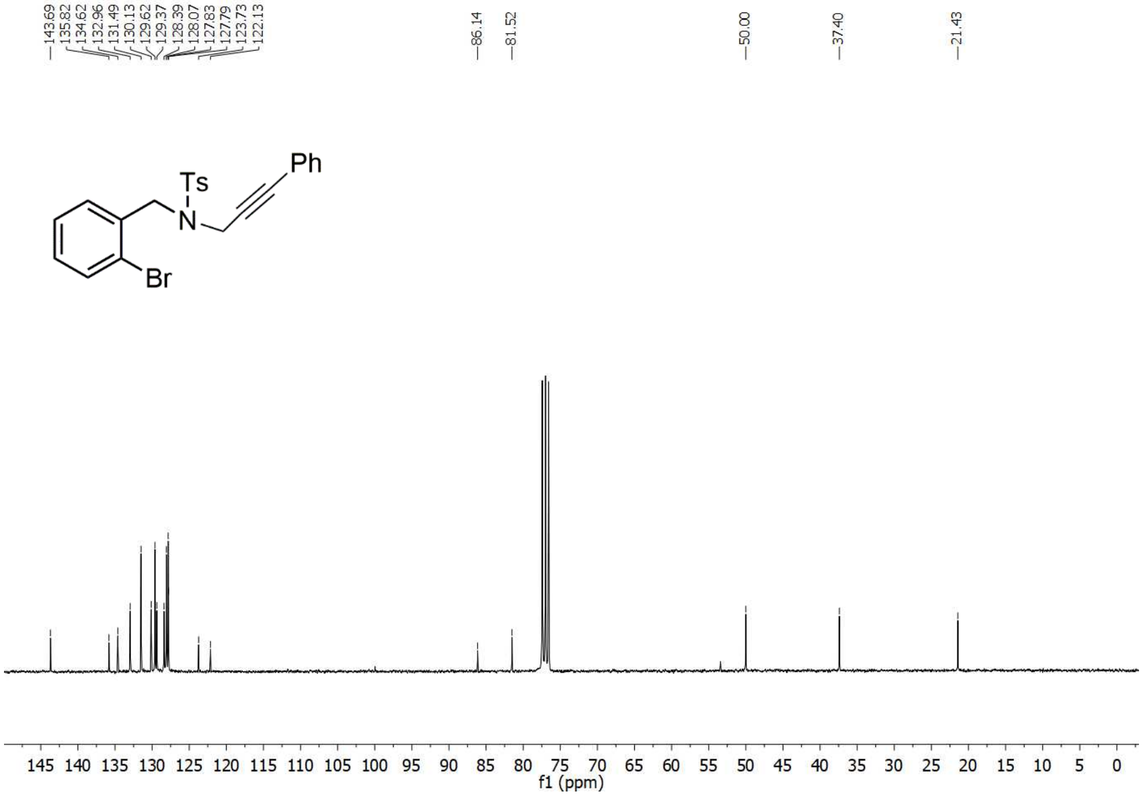

Figure S9. ^{13}C-NMR spectrum (75.45 MHz, $CDCl_3$) of compound **1l**.

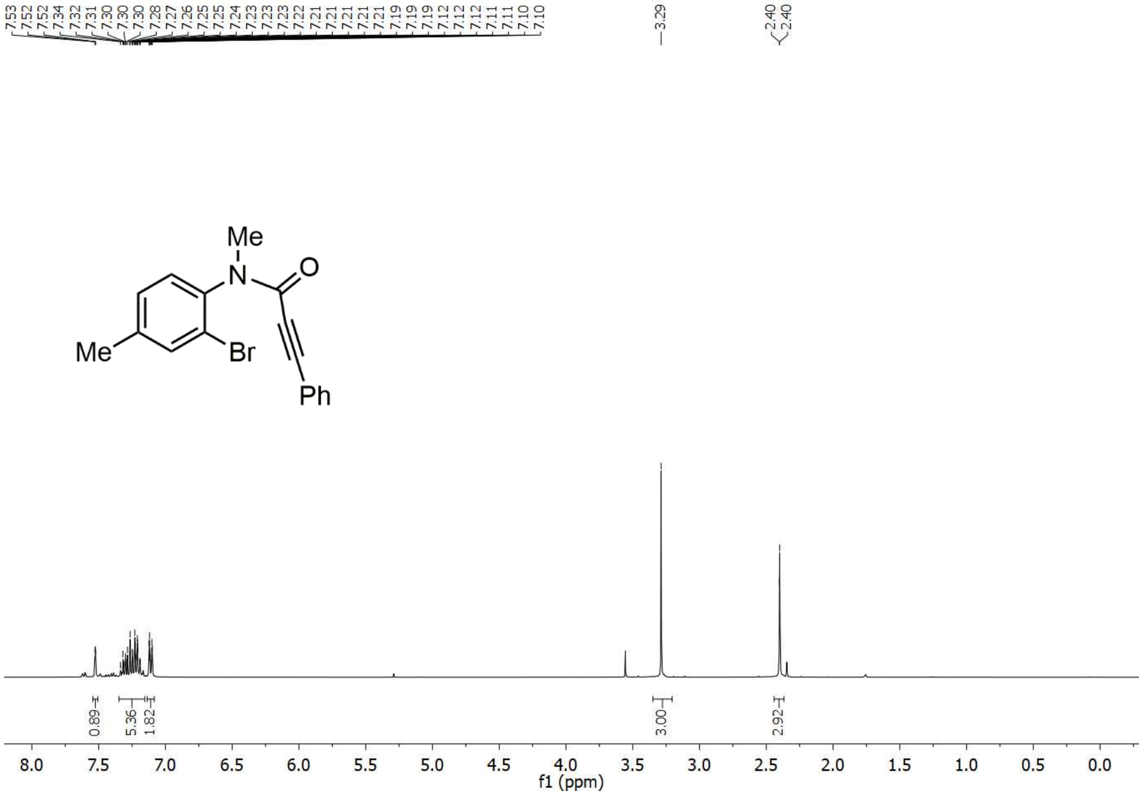

Figure S10. ^{1}H-NMR spectrum (400 MHz, $CDCl_3$) of compound **1n**.

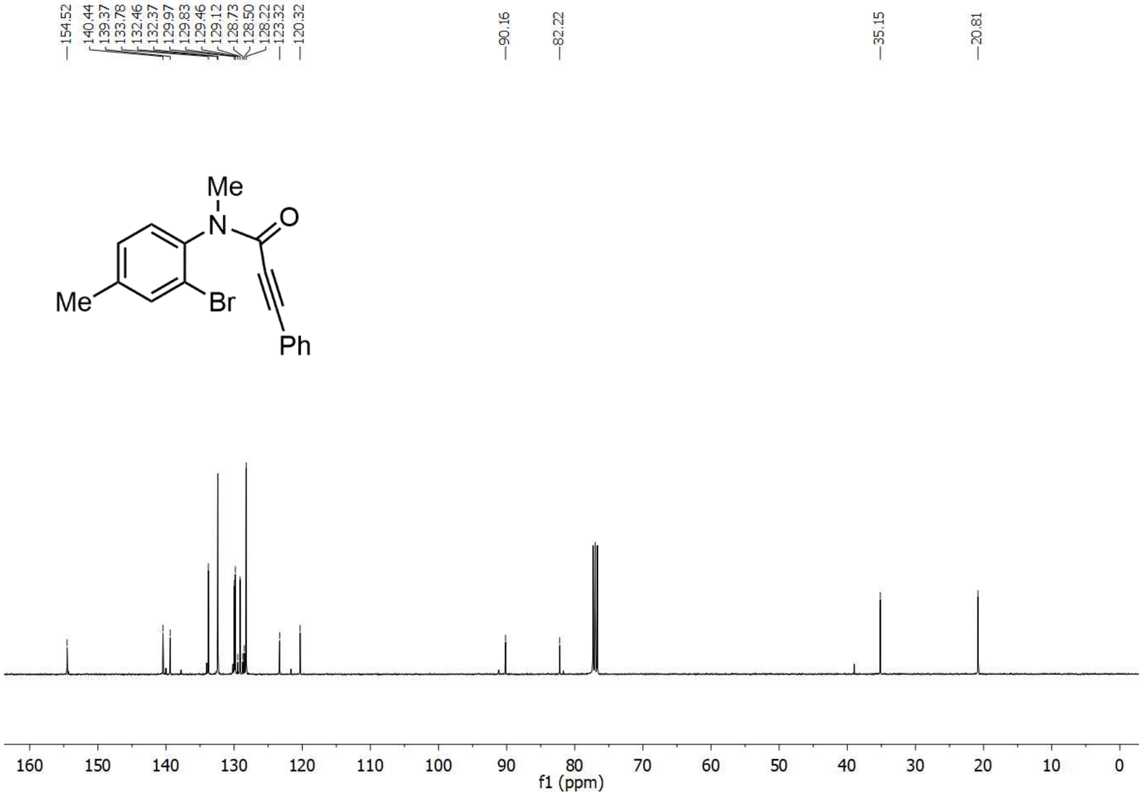

Figure S11. ^{13}C-NMR spectrum (101 MHz, $CDCl_3$) of compound **1n**.

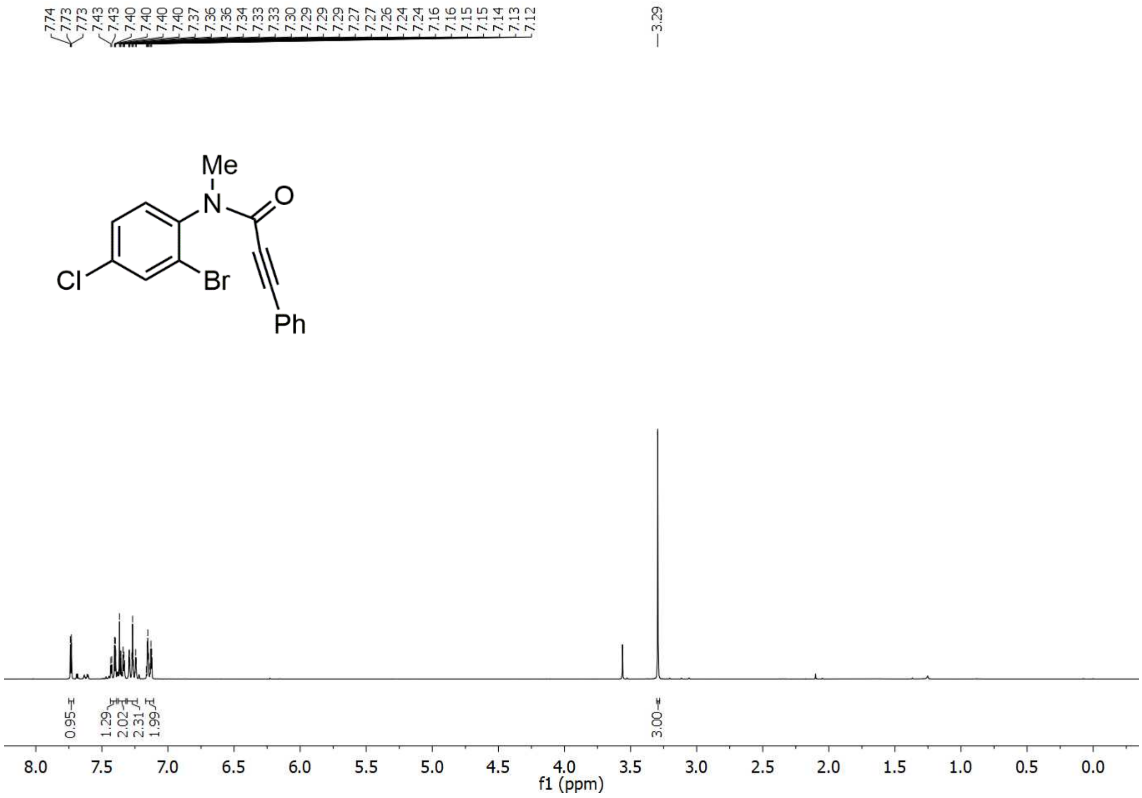

Figure S12. ^{1}H-NMR spectrum (300 MHz, $CDCl_3$) of compound **1o**.

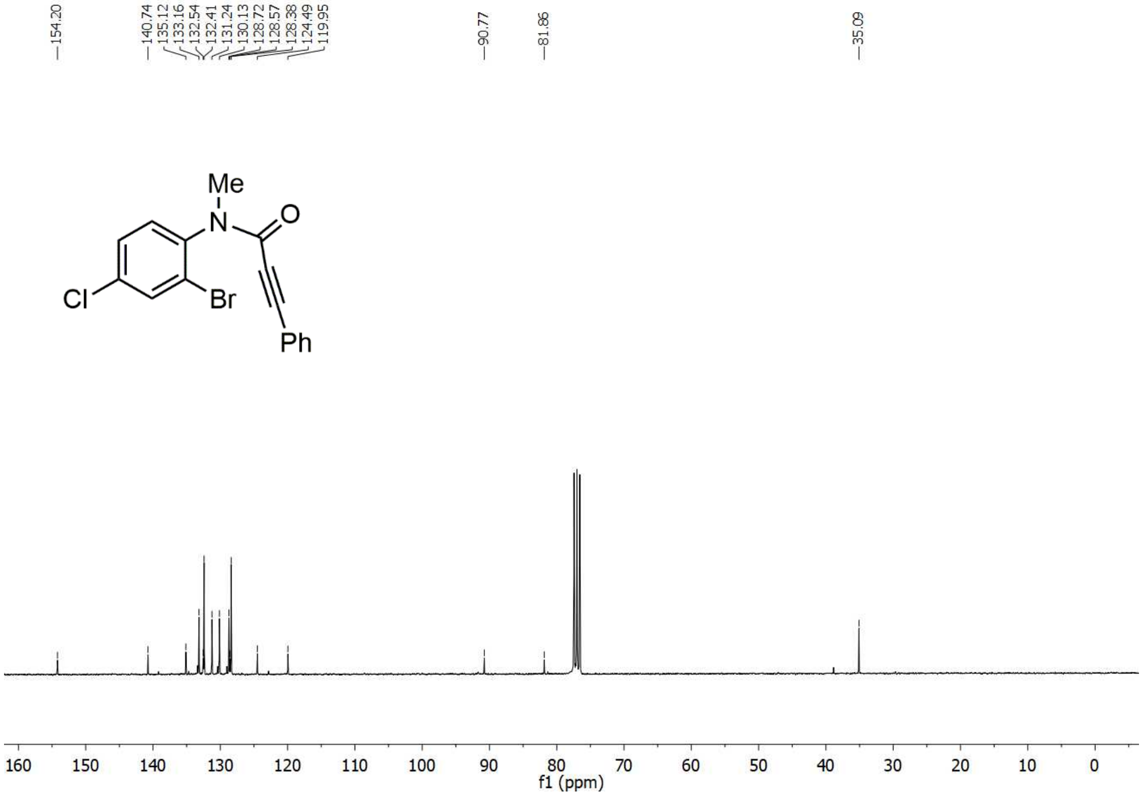

Figure S13. ^{13}C-NMR spectrum (75.45 MHz, $CDCl_3$) of compound **1o**.

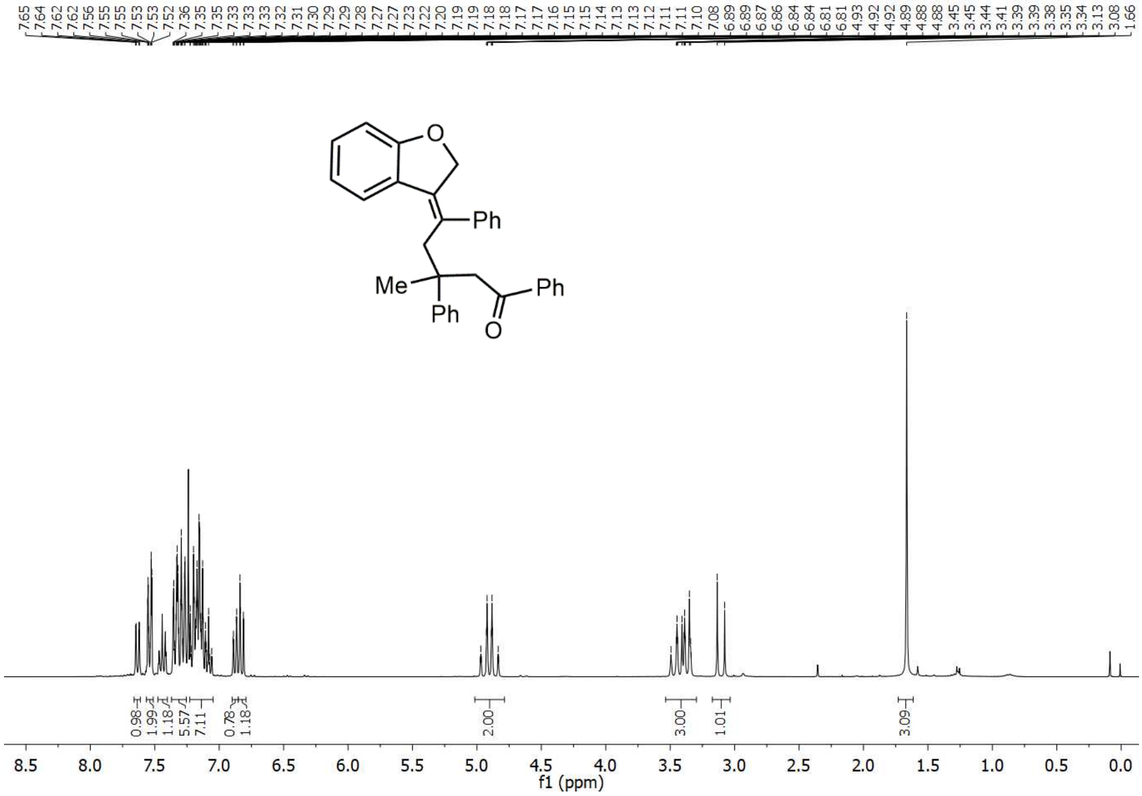

Figure S14. ^{1}H-NMR spectrum (300 MHz, $CDCl_3$) of compound **3a**.

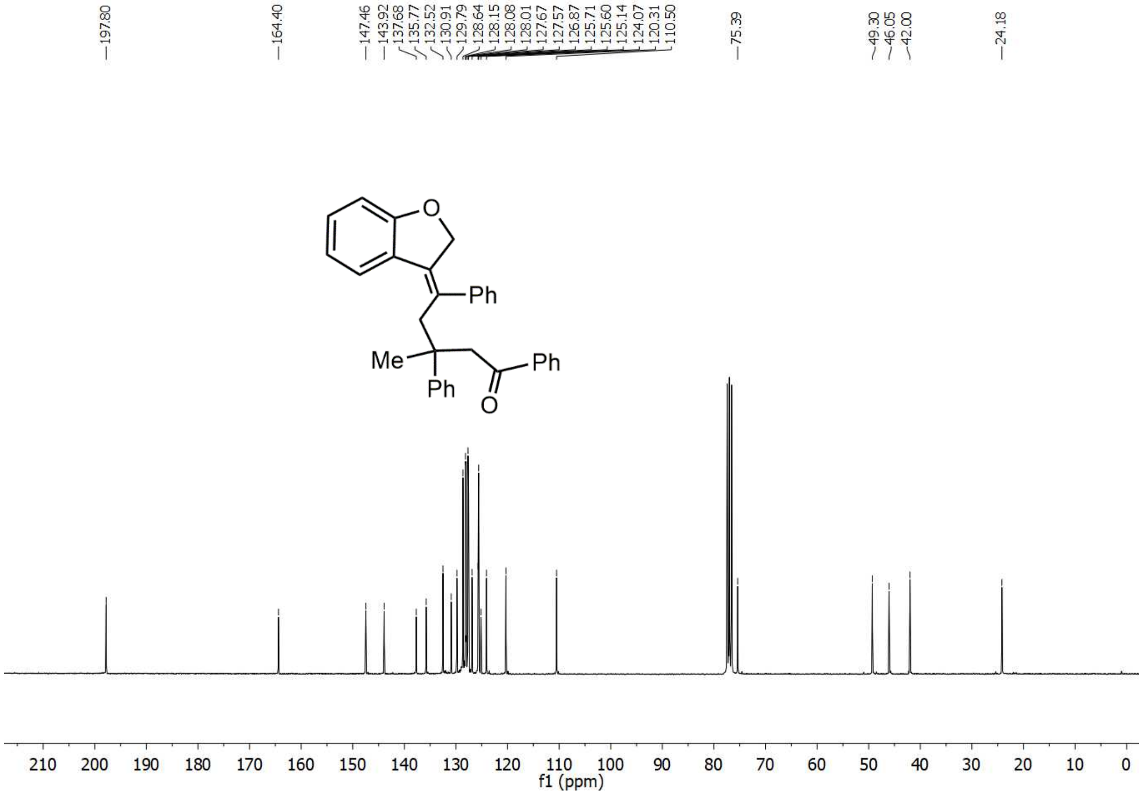

Figure S15. ^{13}C-NMR spectrum (75.45 MHz, $CDCl_3$) of compound **3a**.

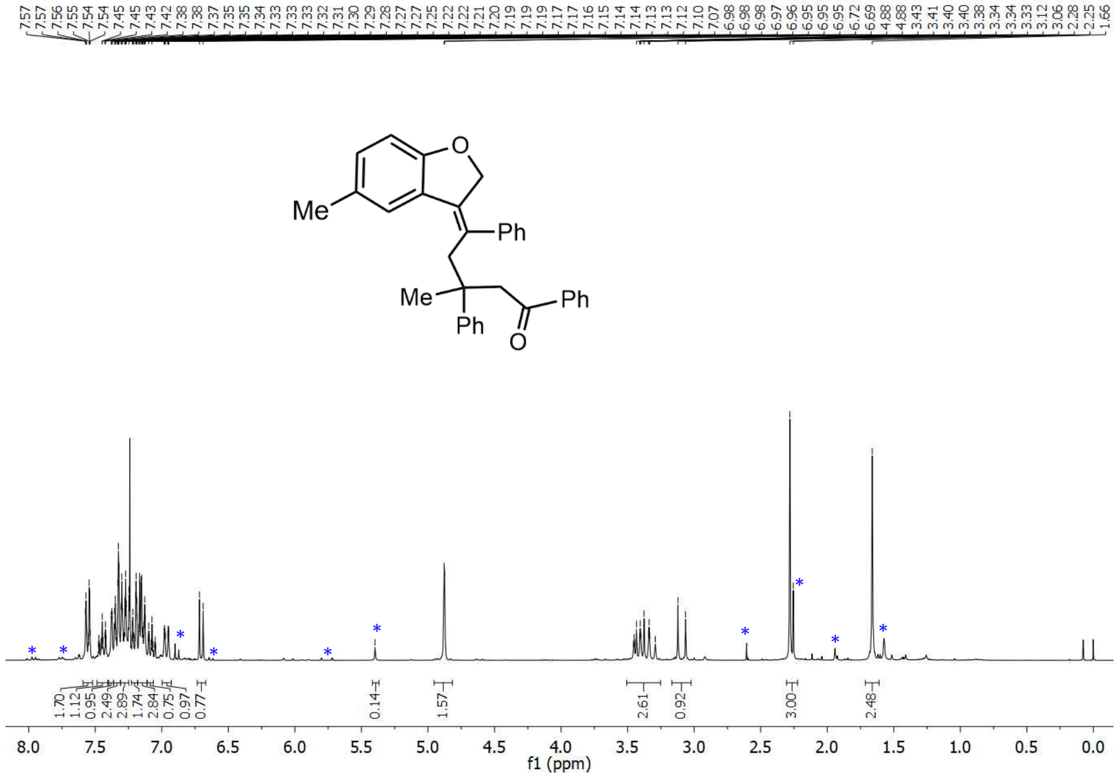

Figure S16. ^{1}H-NMR spectrum (300 MHz, $CDCl_3$) of compound **3b**. Signals marked with blue asterisks correspond to non identified species arising from partial decomposition of **3b**, which were not present in the crude reaction mixture.

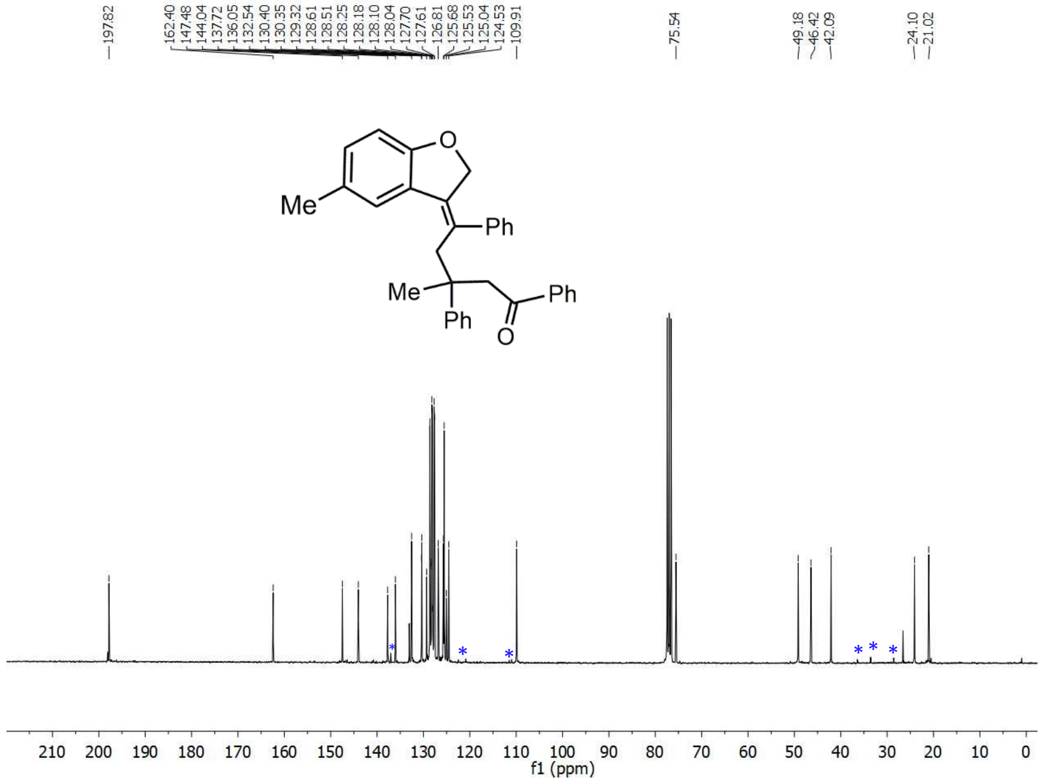

Figure S17. ^{13}C-NMR spectrum (75.45 MHz, $CDCl_3$) of compound **3b**. Signals marked with blue asterisks correspond to non identified species arising from partial decomposition of **3b**, which were not present in the crude reaction mixture.

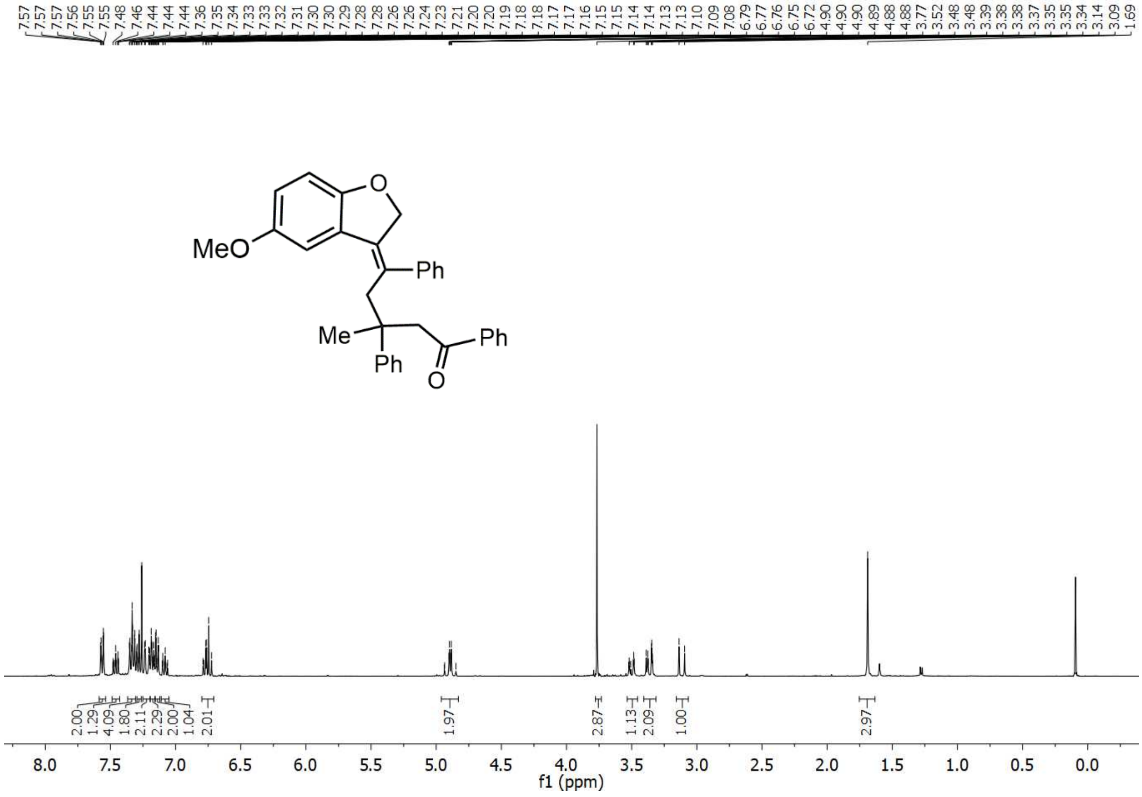

Figure S18. ^{1}H-NMR spectrum (400 MHz, $CDCl_3$) of compound **3c**.

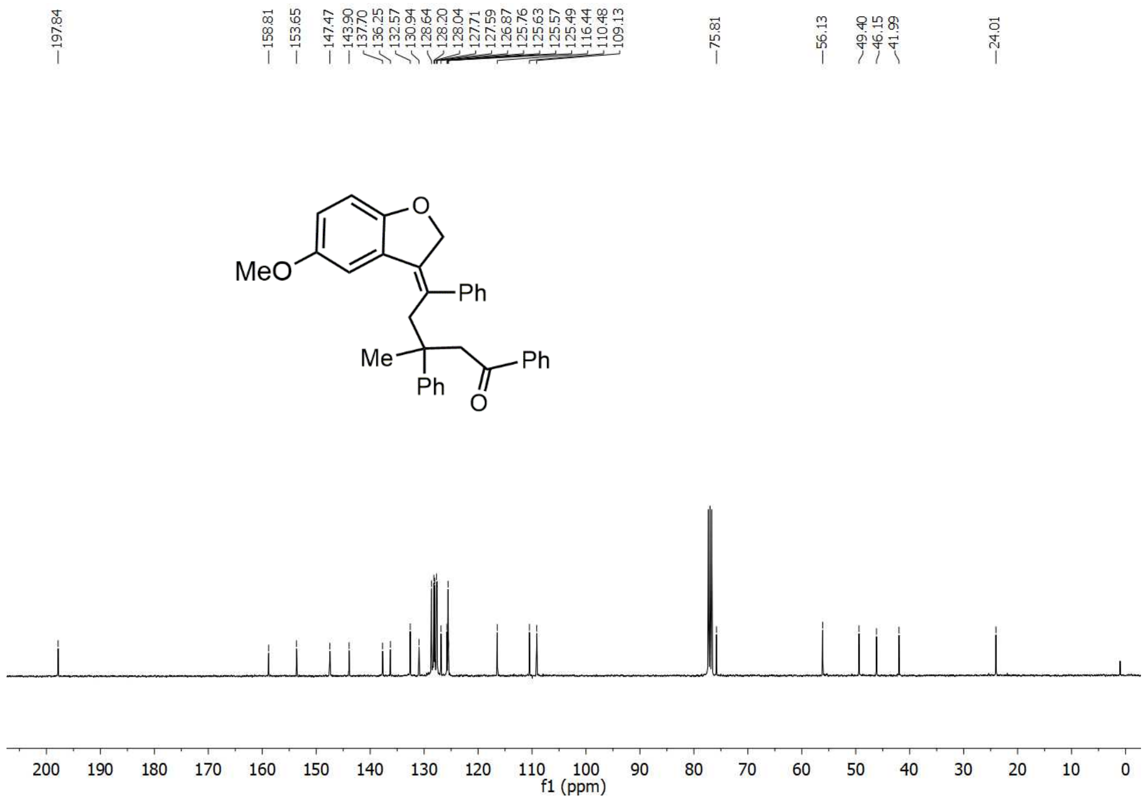

Figure S19. ^{13}C-NMR spectrum (101 MHz, $CDCl_3$) of compound **3c**.

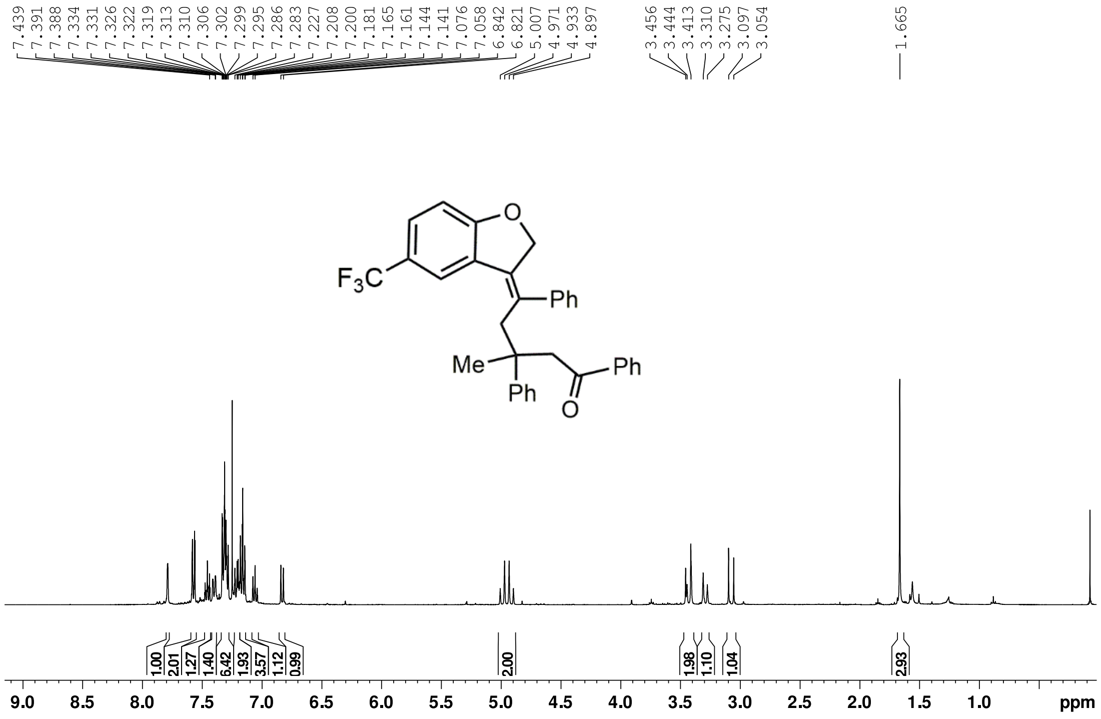

Figure S20. ^{1}H-NMR spectrum (300 MHz, $CDCl_3$) of compound **3d**.

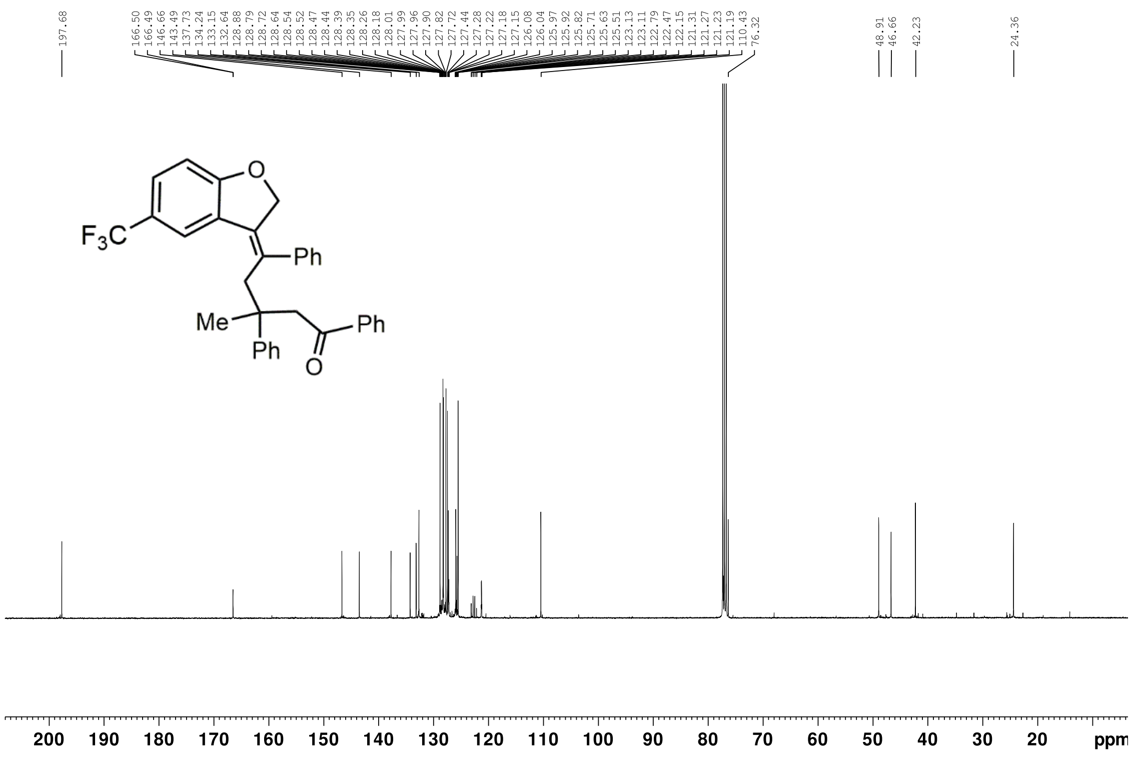

Figure S21. ^{13}C-NMR spectrum (75.45 MHz, $CDCl_3$) of compound **3d**.

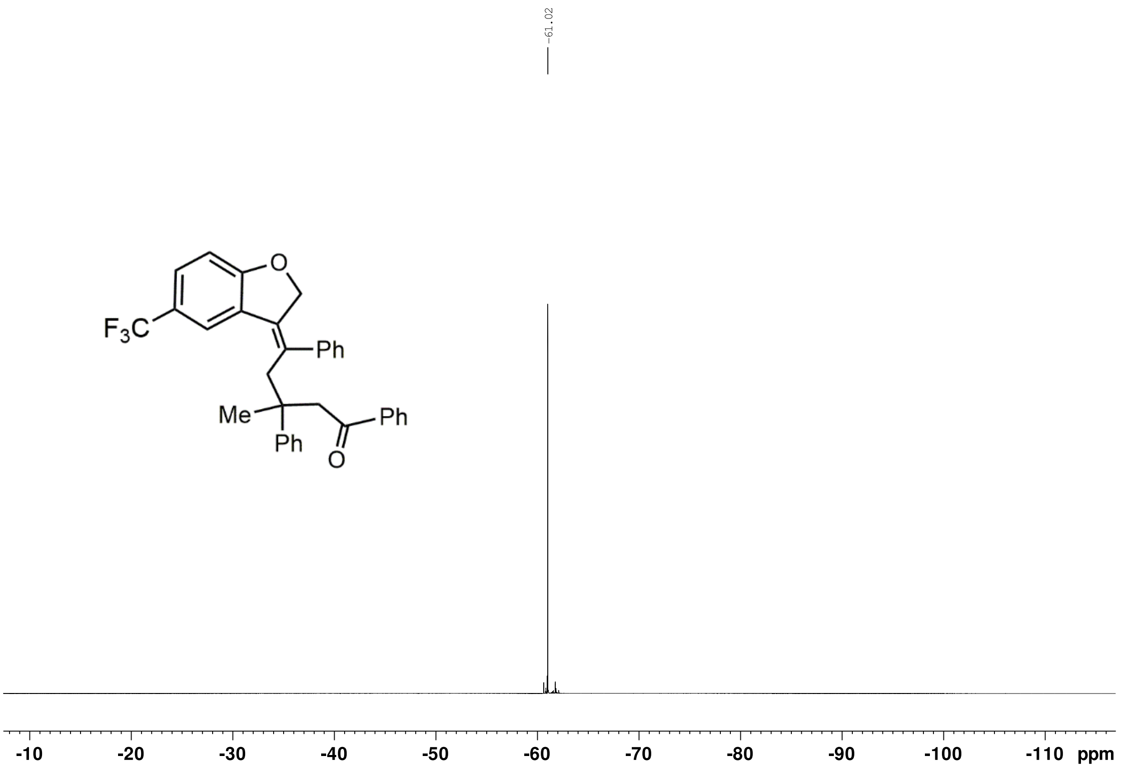

Figure S22. ^{19}F-NMR spectrum (376.5 MHz, $CDCl_3$) of compound **3d**.

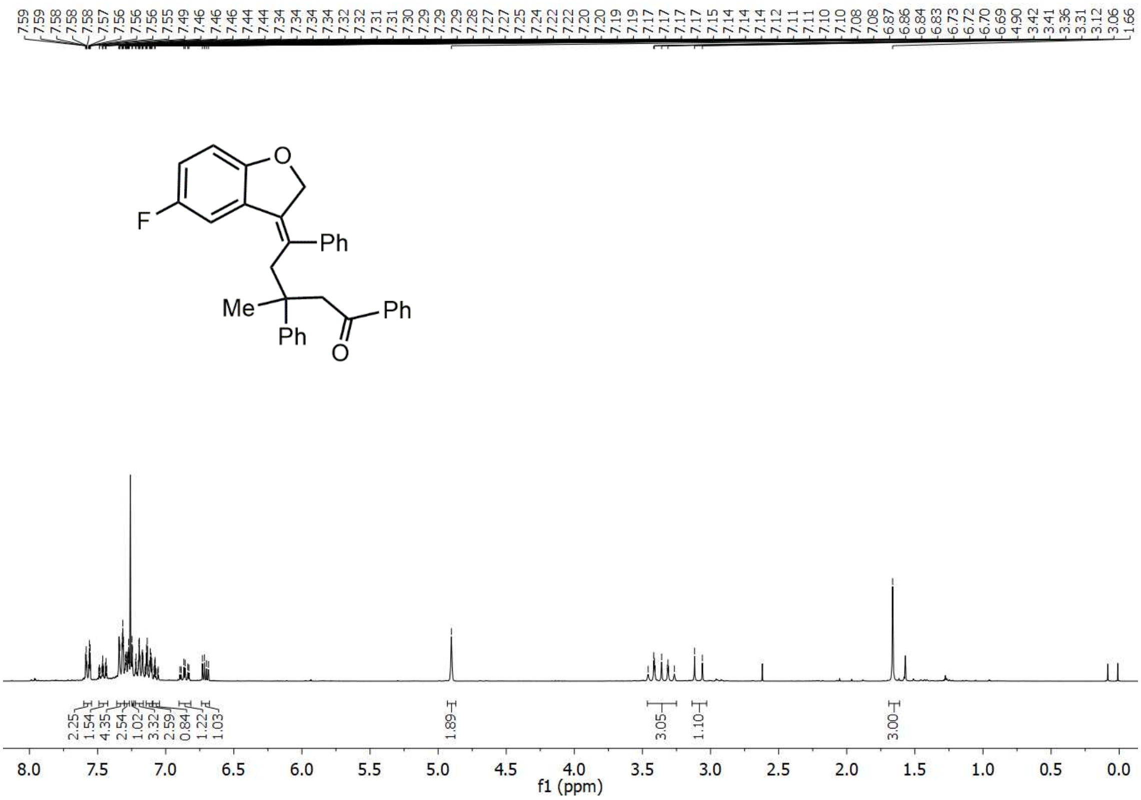

Figure S23. ^{1}H-NMR spectrum (300 MHz, $CDCl_3$) of compound **3e**.

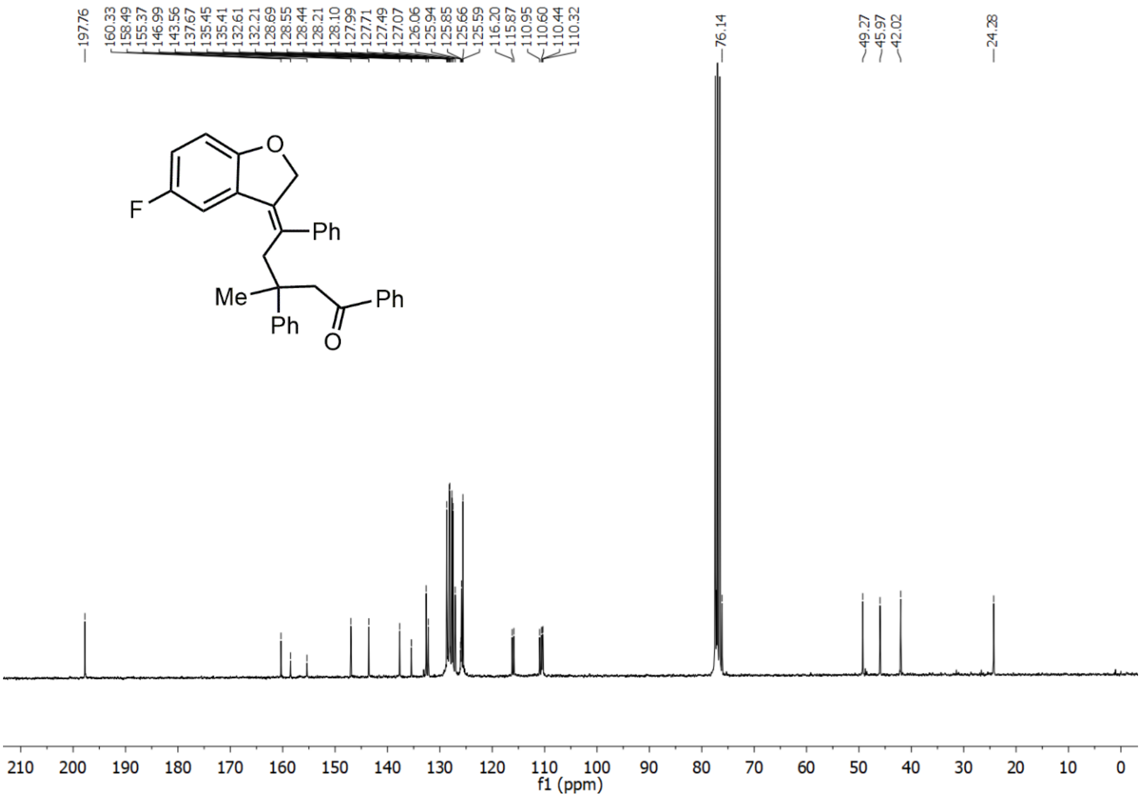

Figure S24. ^{13}C-NMR spectrum (75.45 MHz, $CDCl_3$) of compound **3e**.

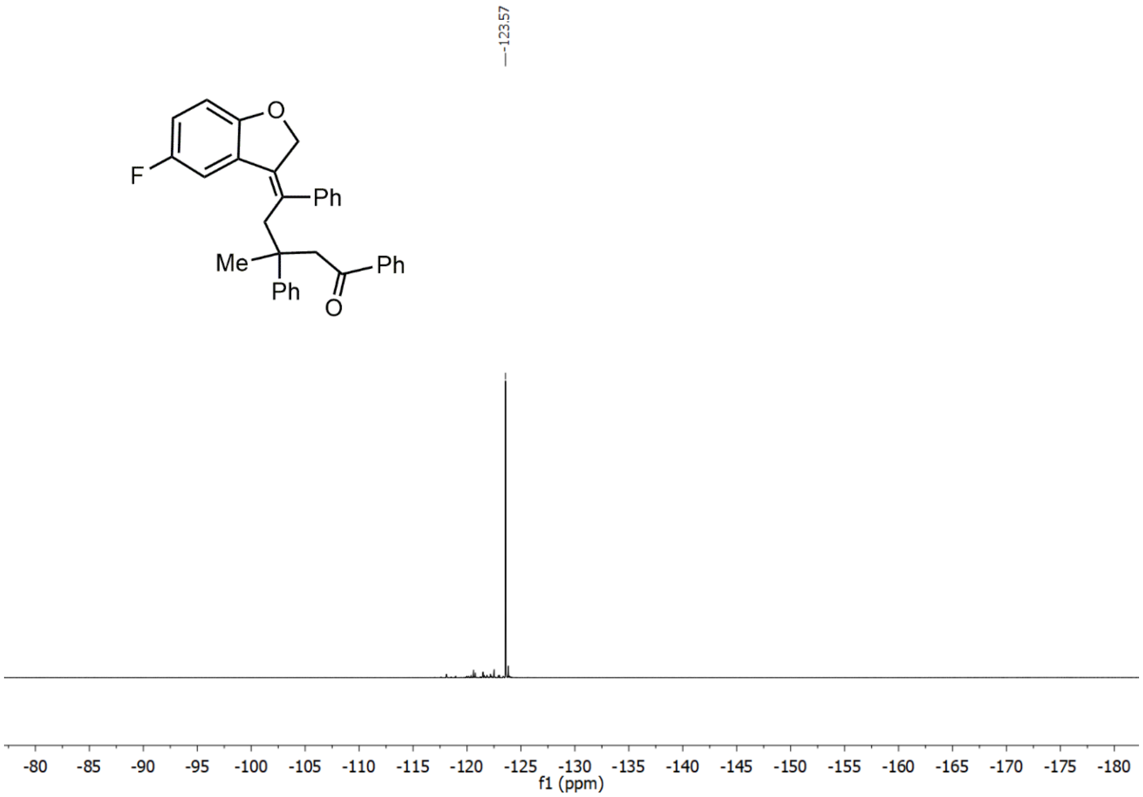

Figure S25. ^{19}F-NMR spectrum (376.5 MHz, $CDCl_3$) of compound **3e**.

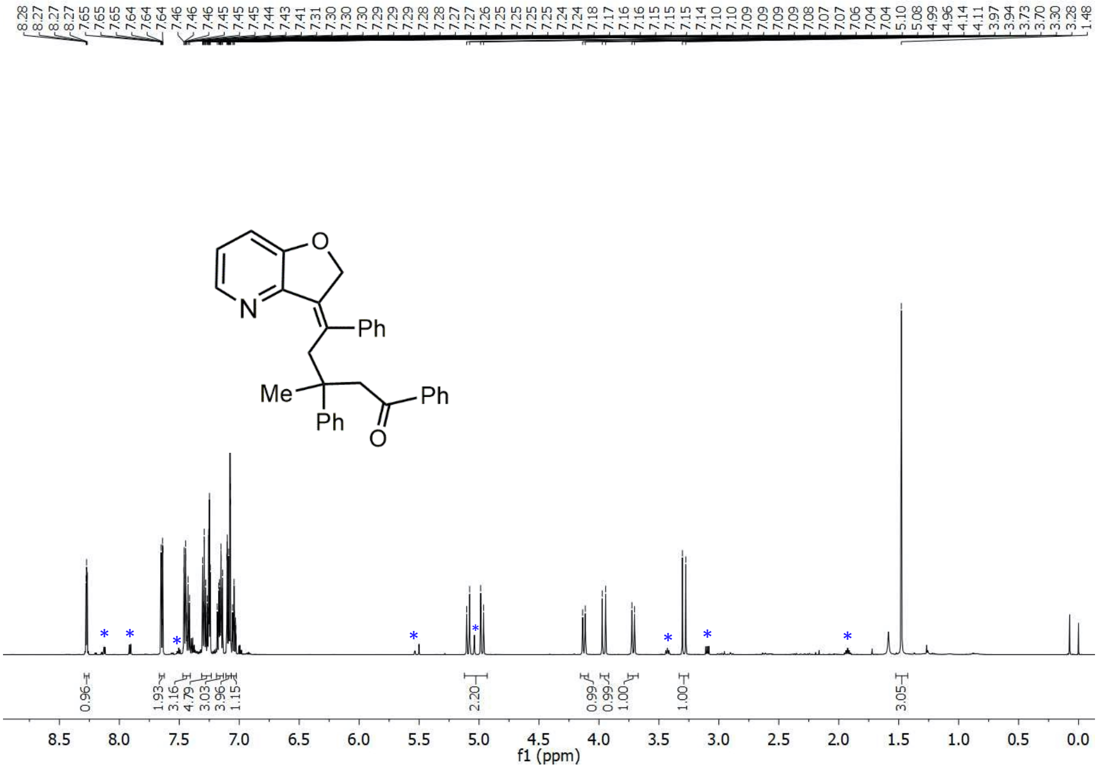

Figure S26. ^{1}H-NMR spectrum (300 MHz, $CDCl_3$) of compound **3f**. Signals marked with blue asterisks correspond to non identified species arising from partial decomposition of **3f**, which were not present in the crude reaction mixture.

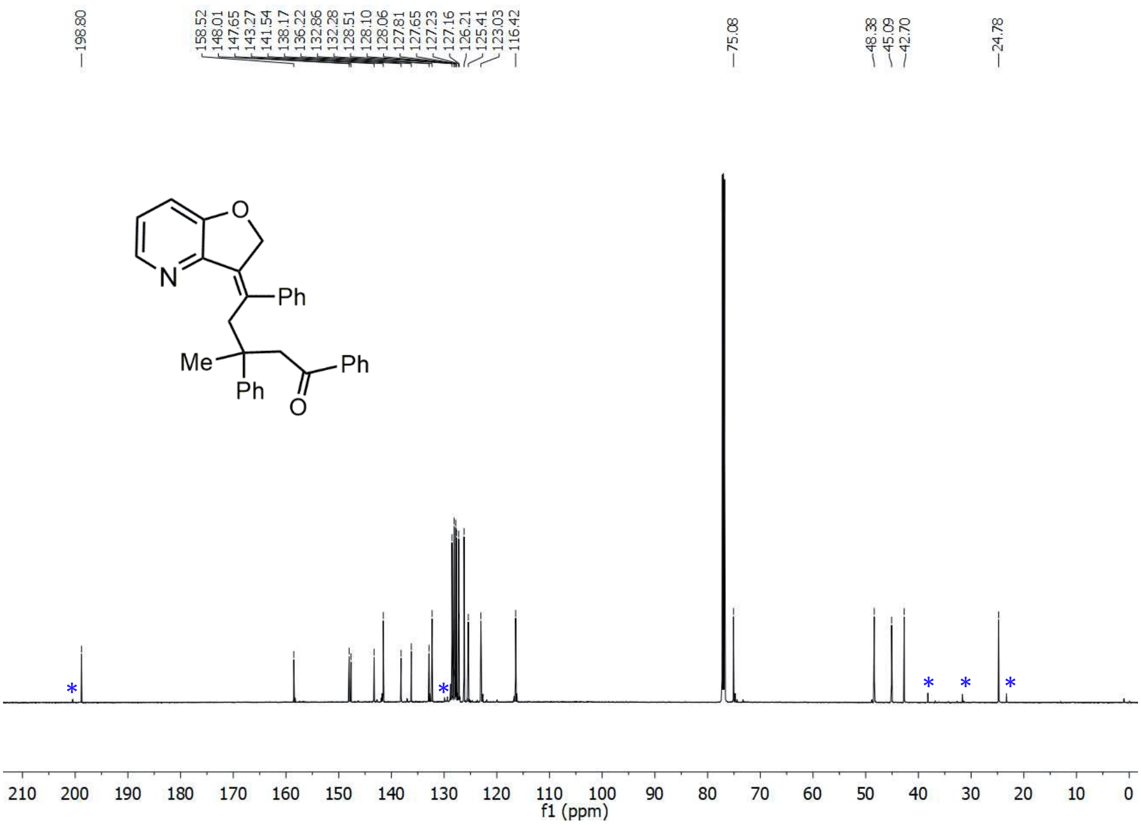

Figure S27. ^{13}C-NMR spectrum (75.45 MHz, $CDCl_3$) of compound **3f**. Signals marked with blue asterisks correspond to non identified species arising from partial decomposition of **3f**, which were not present in the crude reaction mixture.

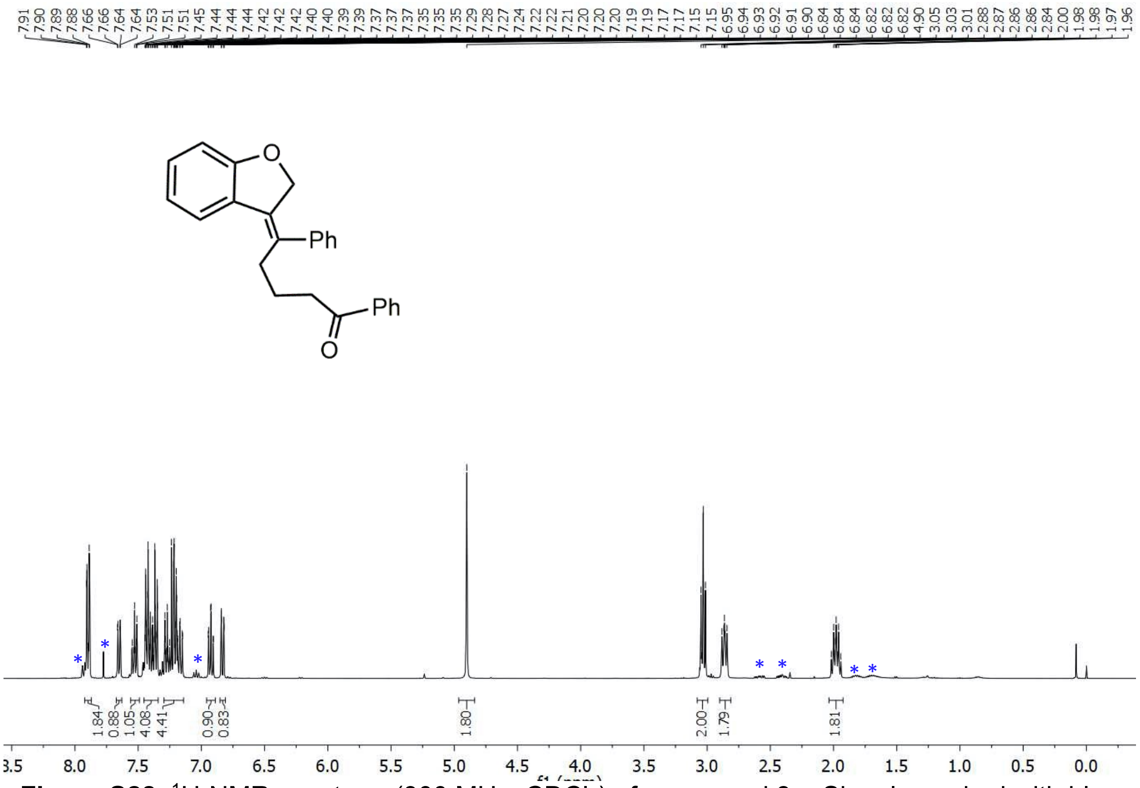

Figure S28. ^{1}H-NMR spectrum (300 MHz, $CDCl_3$) of compound **3g**. Signals marked with blue asterisks correspond to non identified species arising from partial decomposition of **3g**, which were not present in the crude reaction mixture.

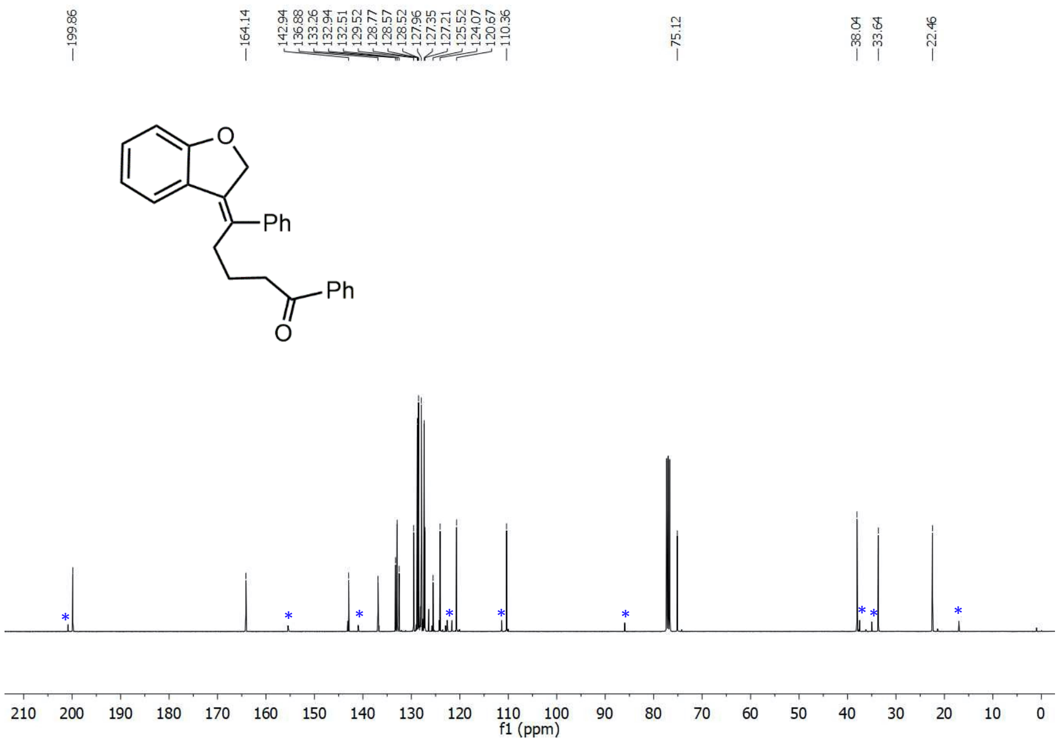

Figure S29. ^{13}C-NMR spectrum (75.45 MHz, $CDCl_3$) of compound **3g**. Signals marked with blue asterisks correspond to non identified species arising from partial decomposition of **3g**, which were not present in the crude reaction mixture.

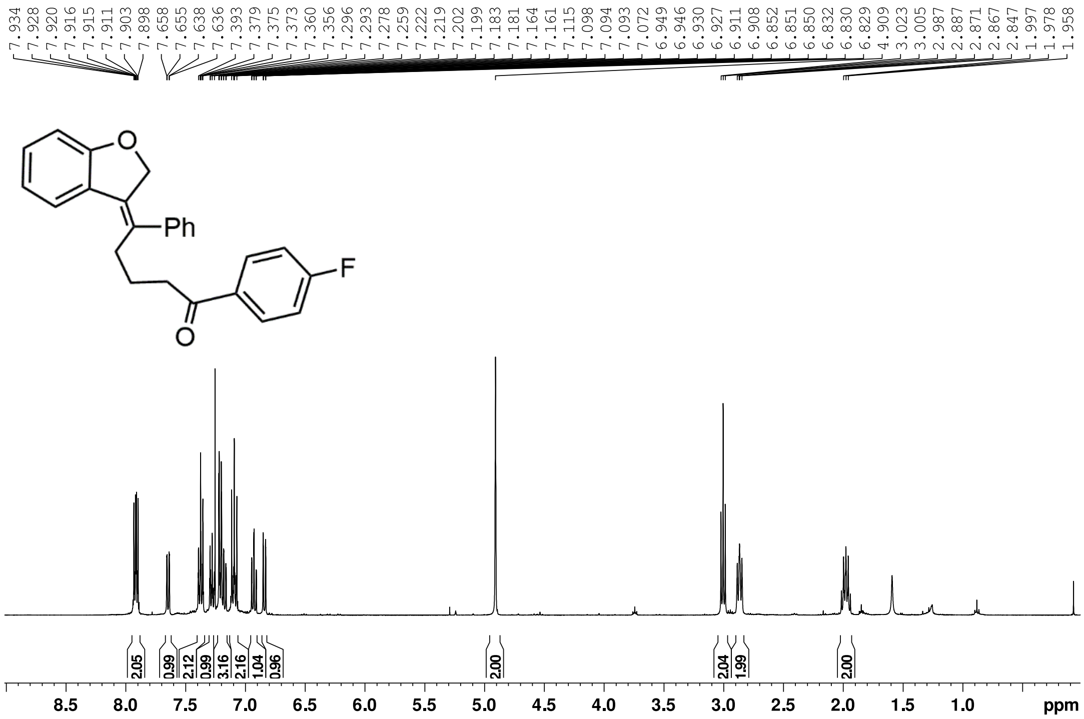

Figure S30. ^{1}H-NMR spectrum (300 MHz, $CDCl_3$) of compound **3h**.

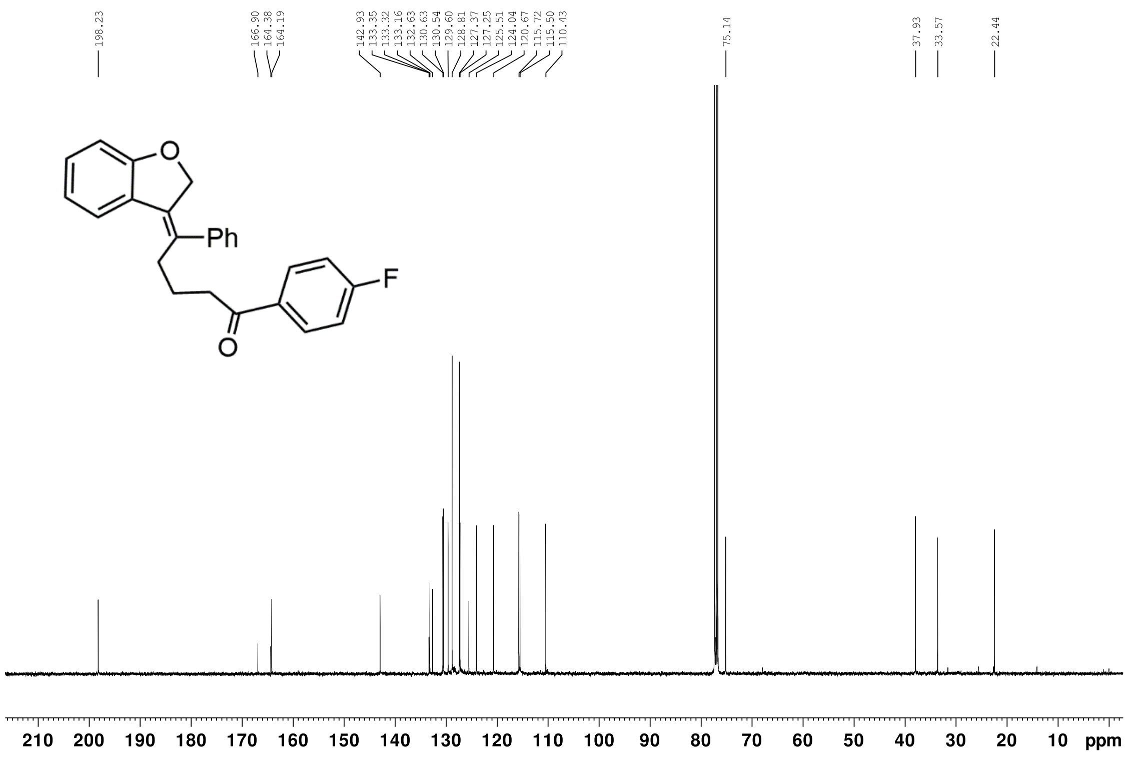

Figure S31. ^{13}C-NMR spectrum (75.45 MHz, $CDCl_3$) of compound **3h**.

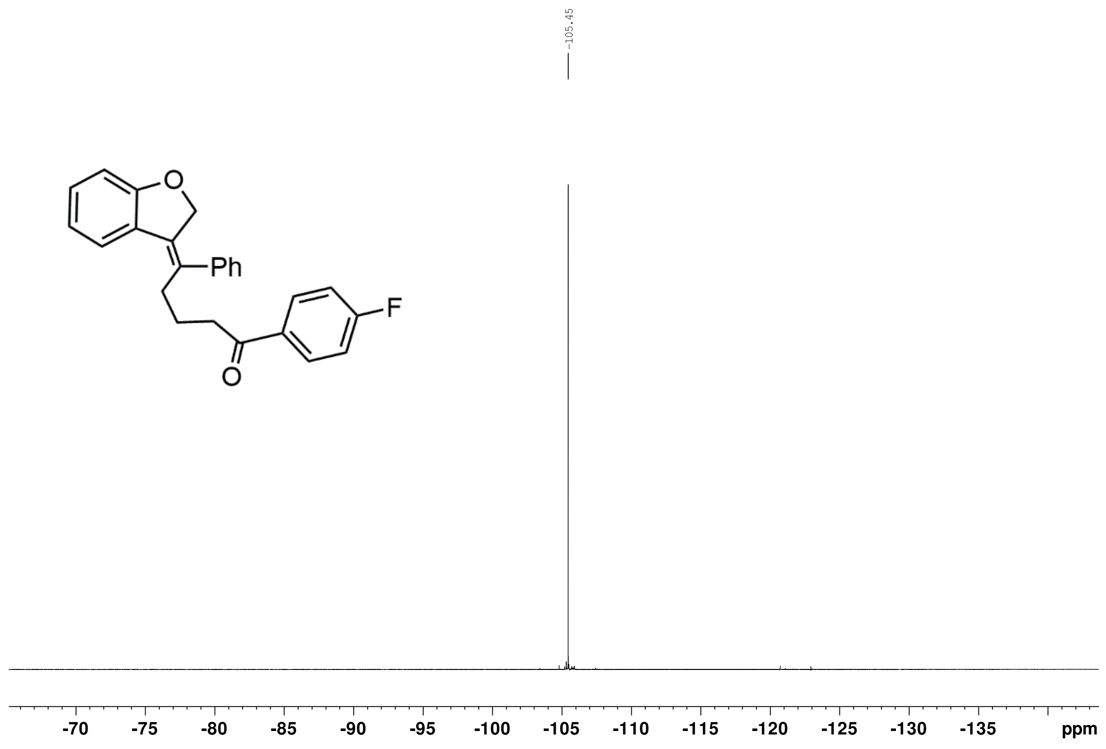

Figure S32. ^{19}F-NMR spectrum (282.4 MHz, $CDCl_3$) of compound **3h**.

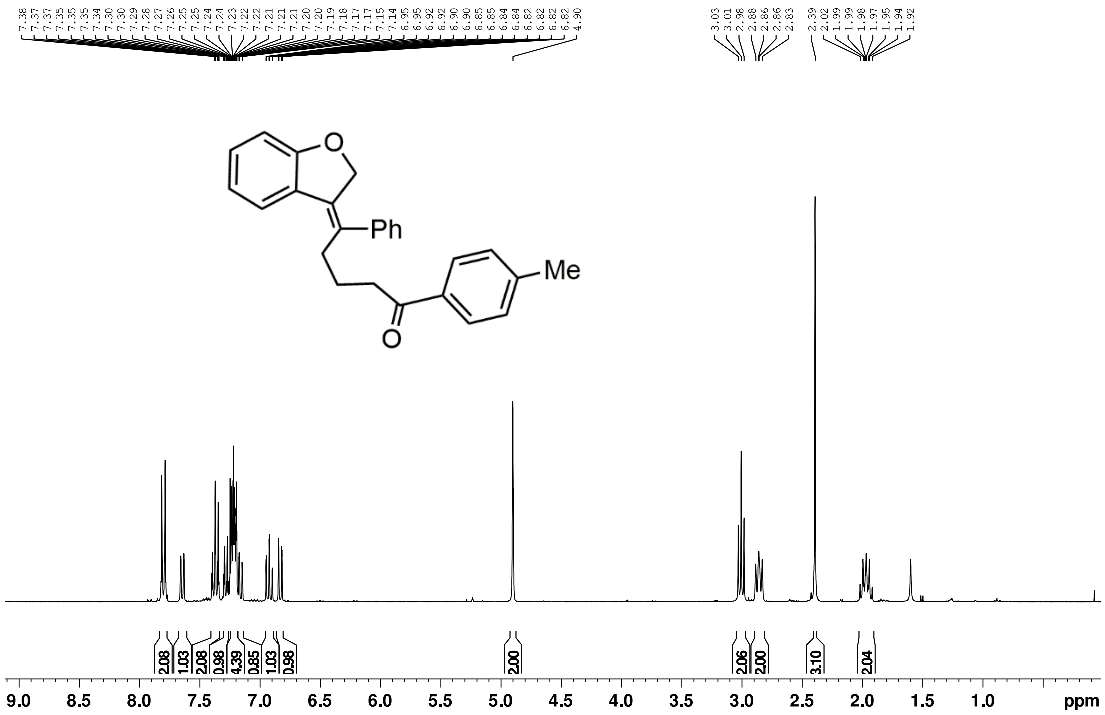

Figure S33. ^{1}H-NMR spectrum (300 MHz, $CDCl_3$) of compound **3i**.

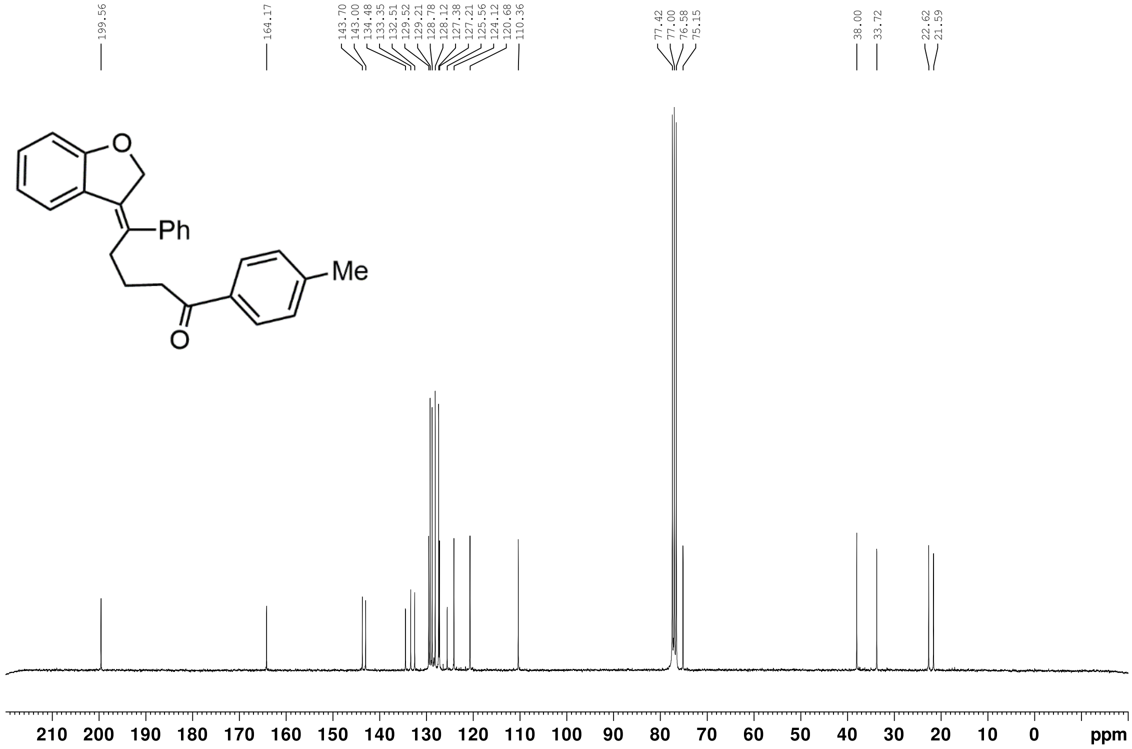

Figure S34. ^{13}C-NMR spectrum (75.45 MHz, $CDCl_3$) of compound **3i**.

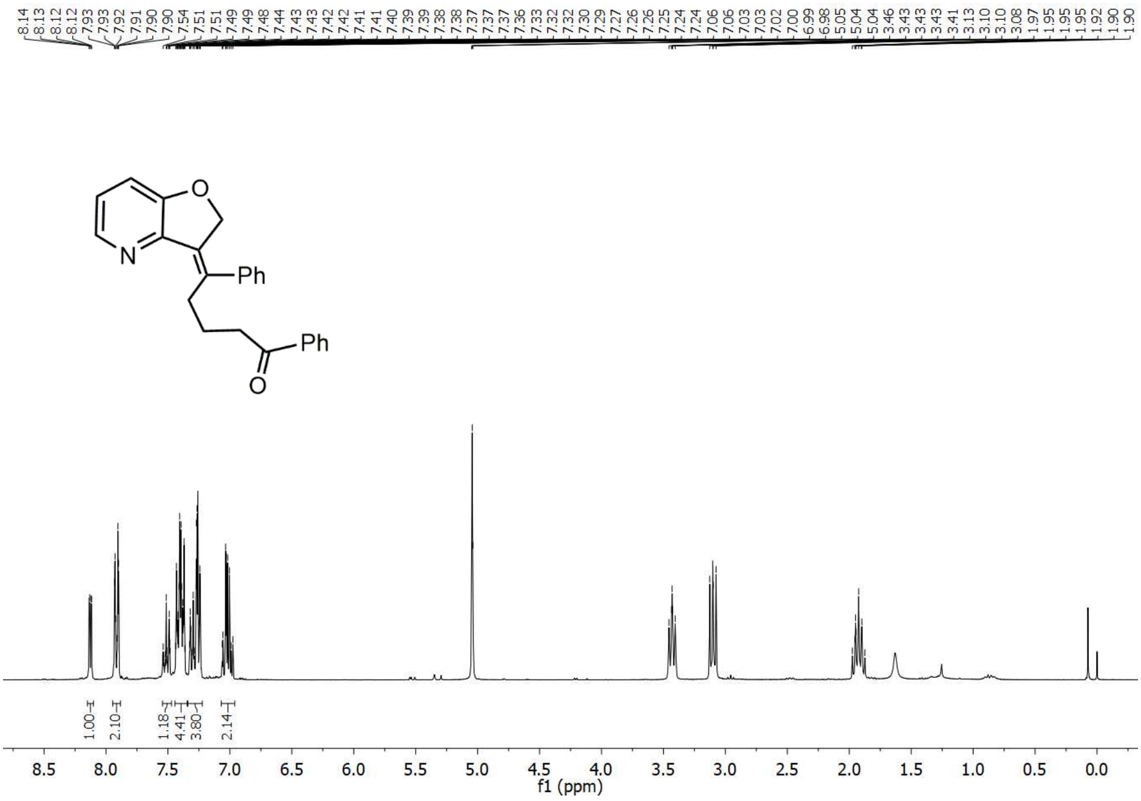

Figure S35. ^{1}H-NMR spectrum (600 MHz, $CDCl_3$) of compound **3j**.

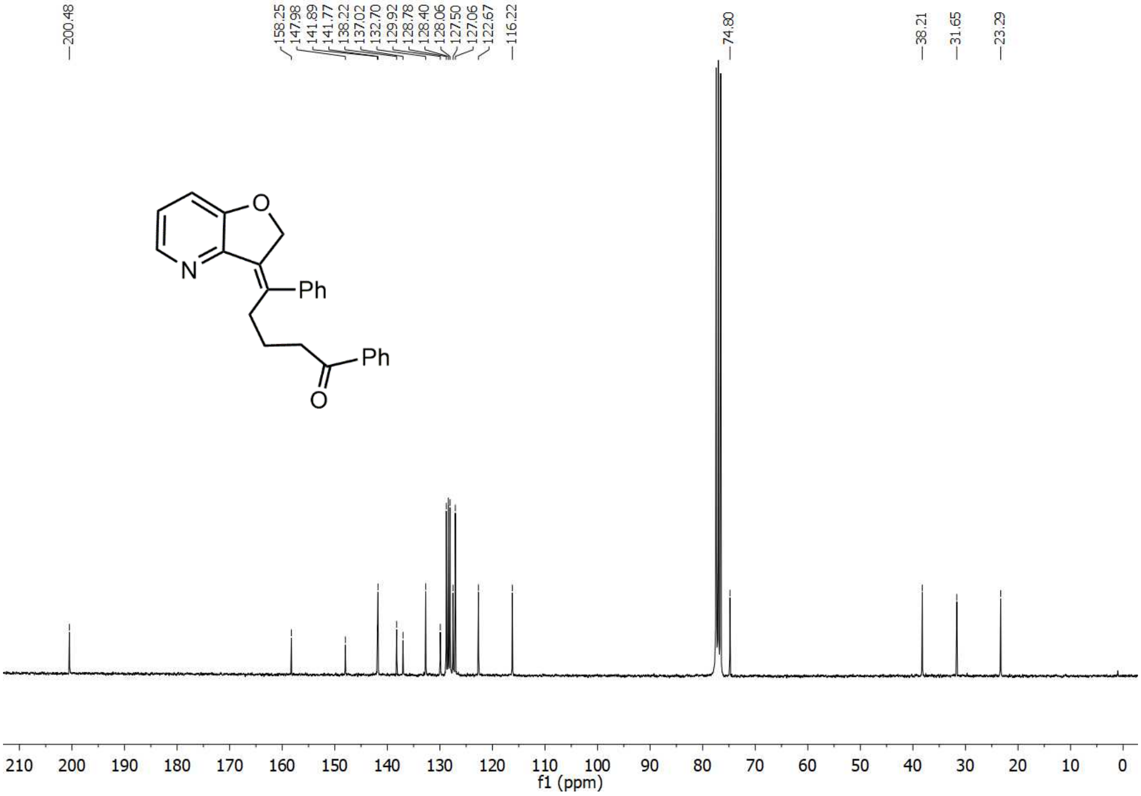

Figure S36. ^{13}C-NMR spectrum (151 MHz, $CDCl_3$) of compound **3j**.

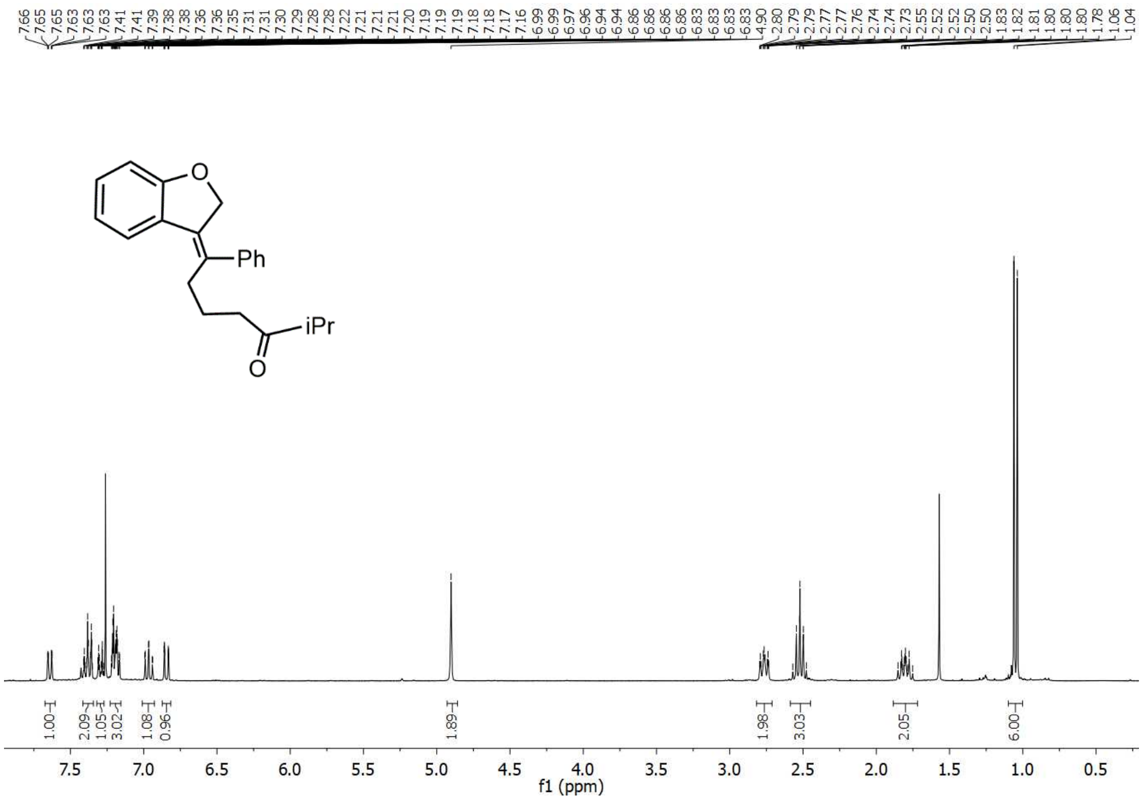

Figure S37. ^{1}H-NMR spectrum (300 MHz, $CDCl_3$) of compound **3l**.

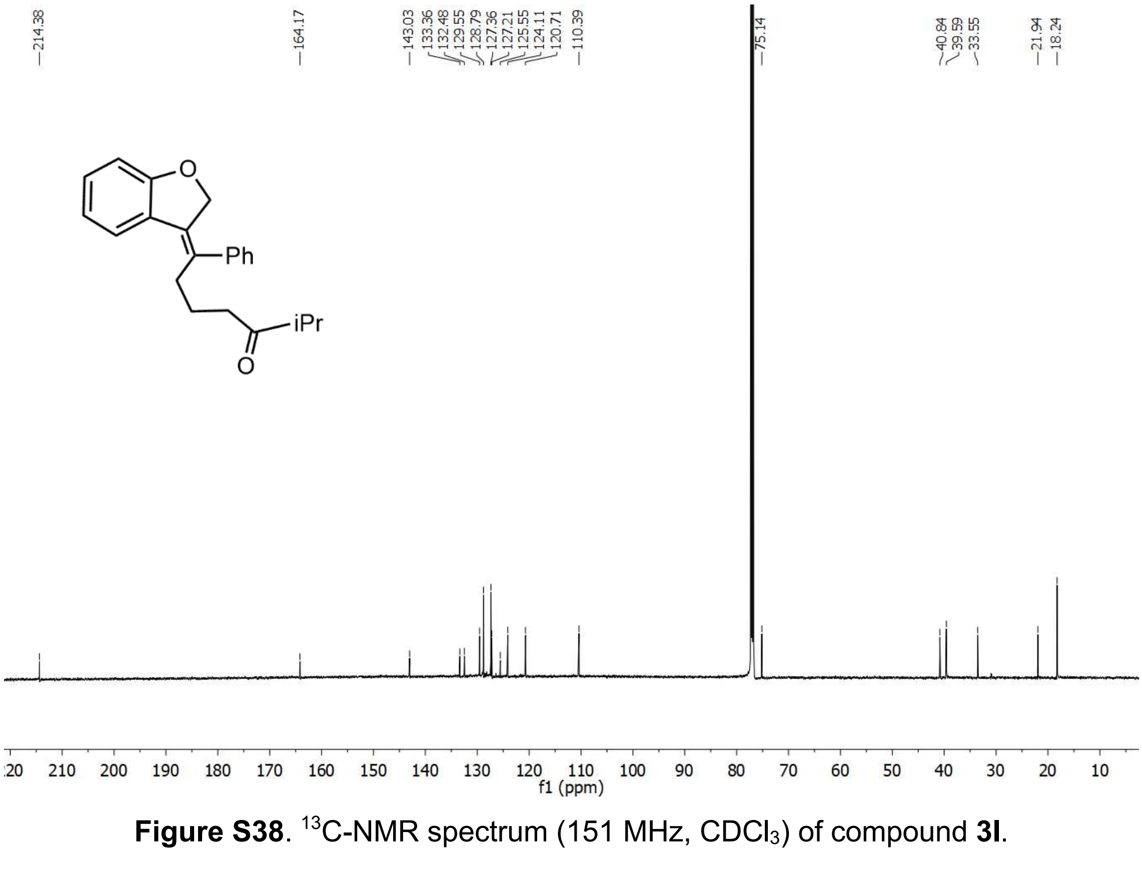

Figure S38. ^{13}C-NMR spectrum (151 MHz, $CDCl_3$) of compound **3l**.

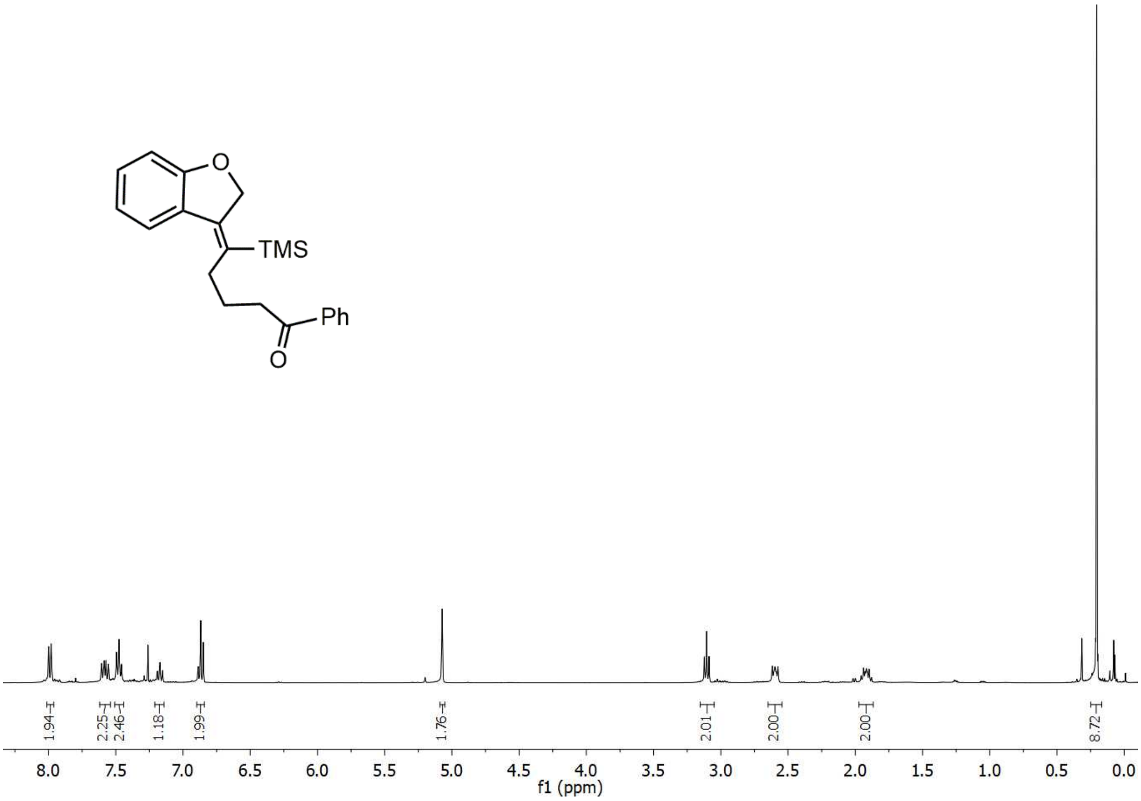

Figure S39. ^{1}H-NMR spectrum (400 MHz, $CDCl_3$) of compound **3m**.

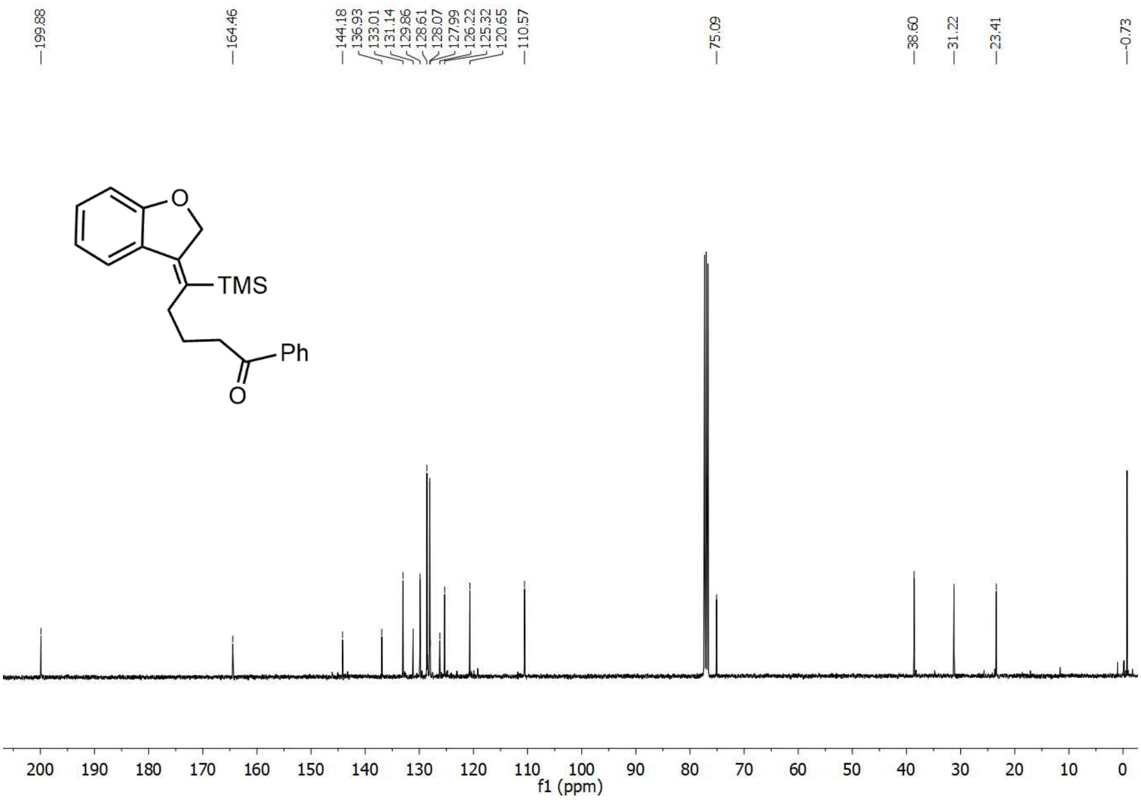

Figure S40. ^{13}C-NMR spectrum (100.1 MHz, $CDCl_3$) of compound **3m**.

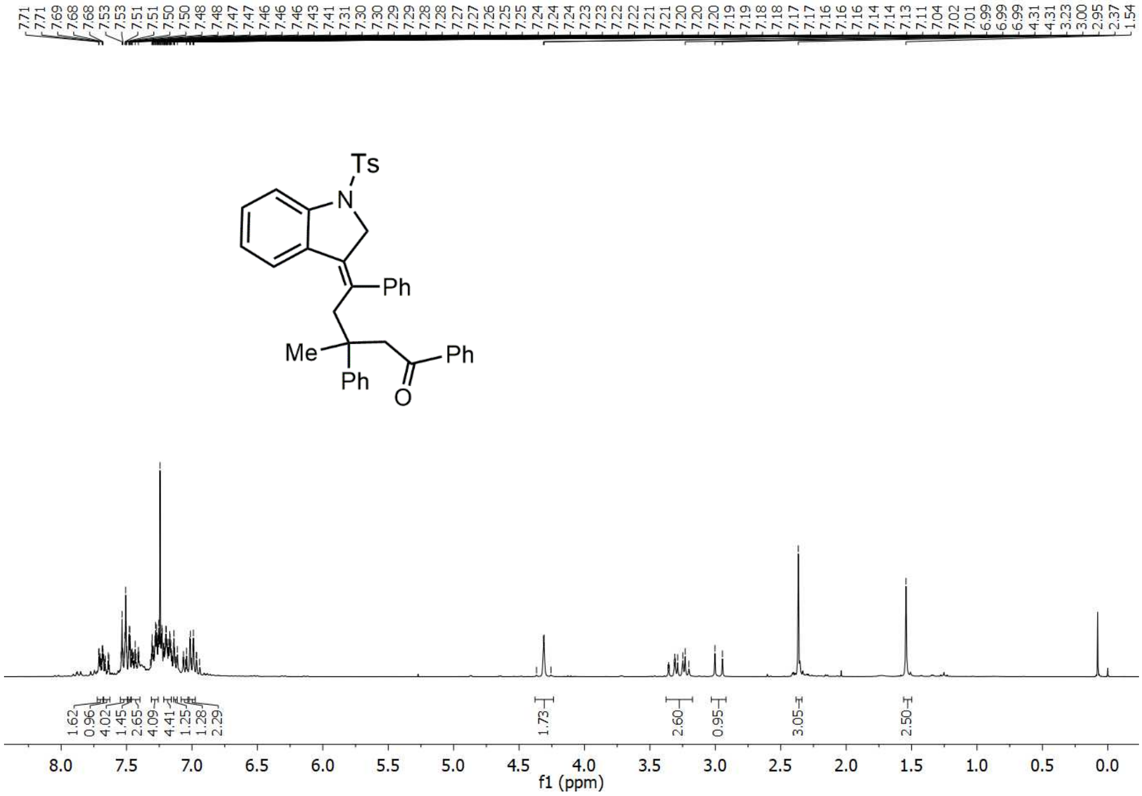

Figure S41. ^{1}H-NMR spectrum (300 MHz, $CDCl_3$) of compound **3o**.

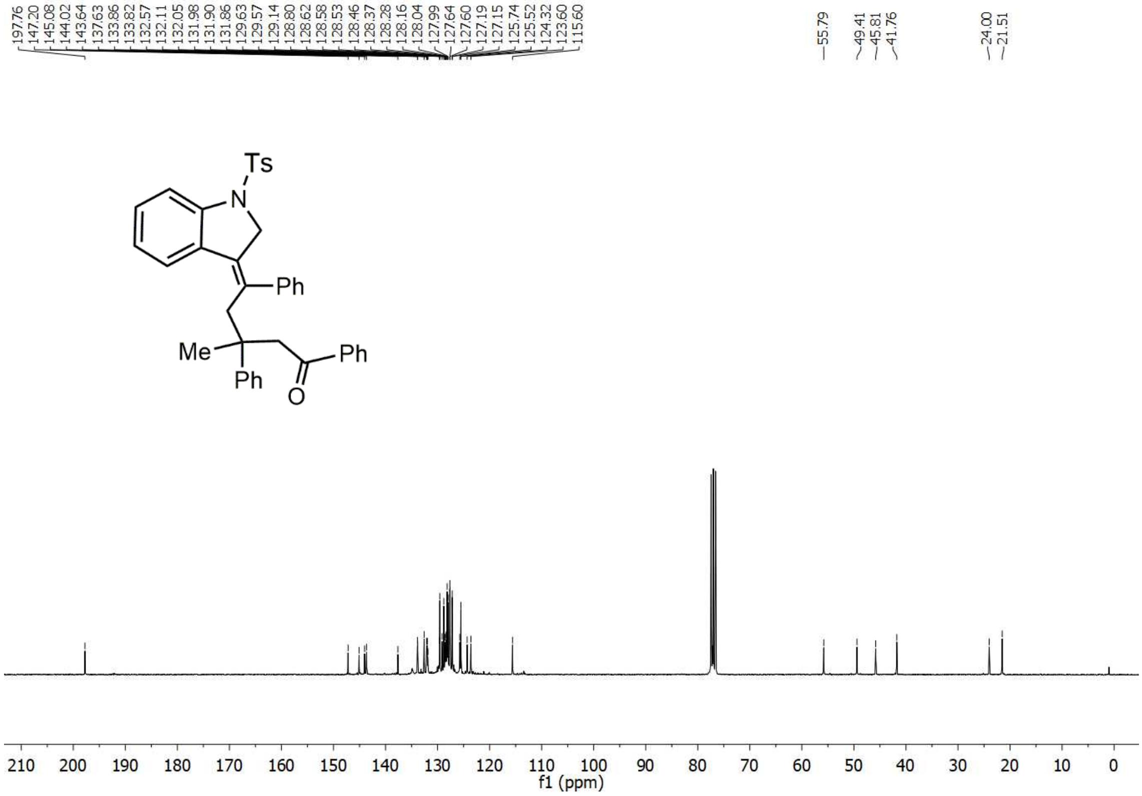

Figure S42. ^{13}C-NMR spectrum (75.45 MHz, $CDCl_3$) of compound **3o**.

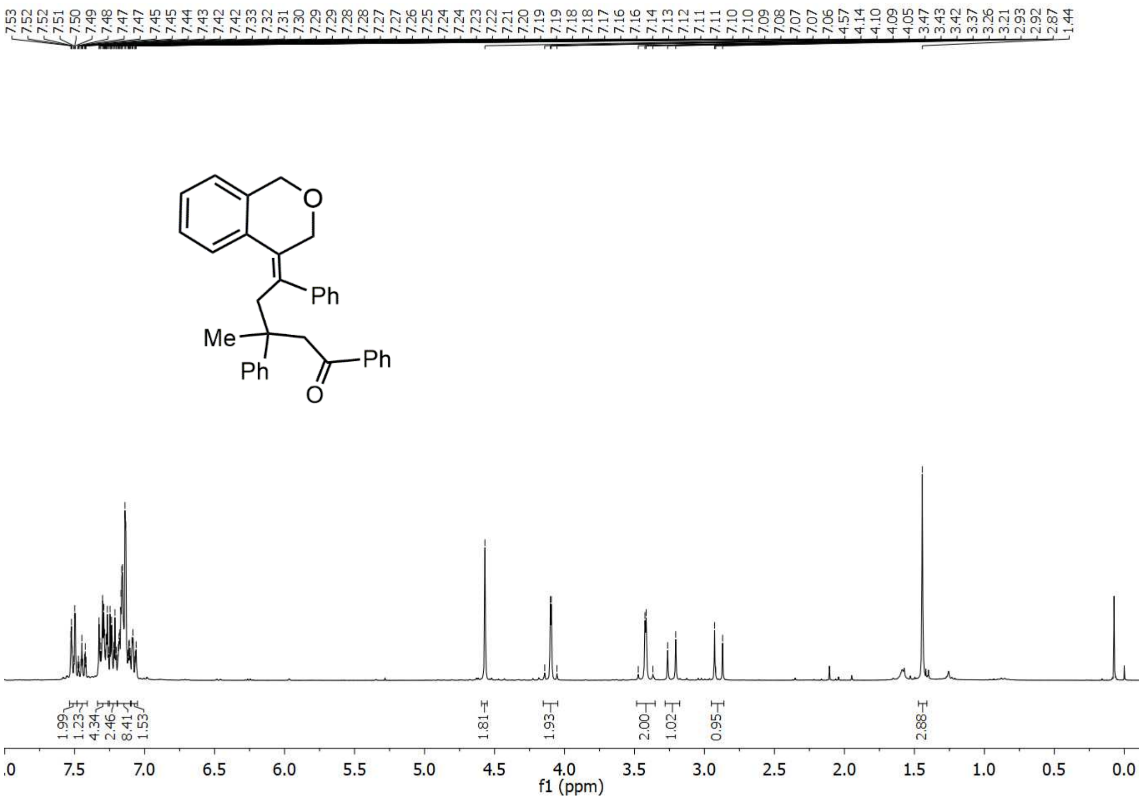

Figure S43. ^{1}H-NMR spectrum (300 MHz, $CDCl_3$) of compound **3q**.

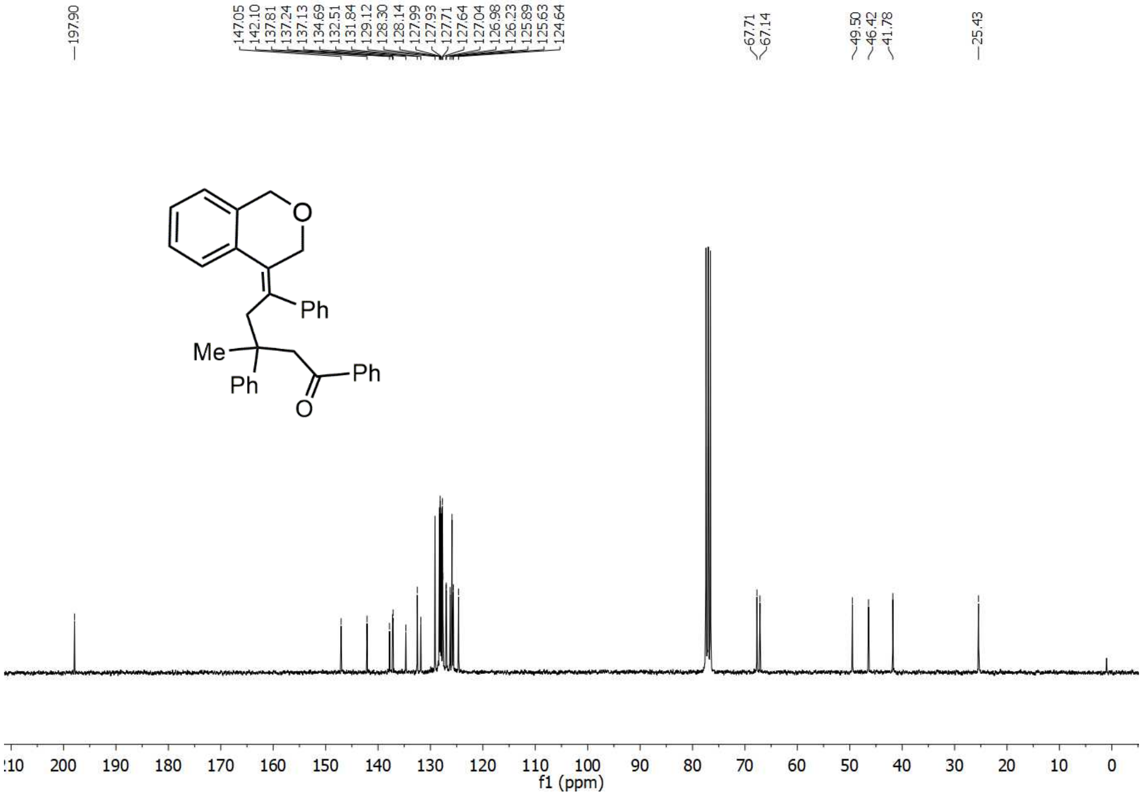

Figure S44. ^{13}C-NMR spectrum (75.45 MHz, $CDCl_3$) of compound **3q**.

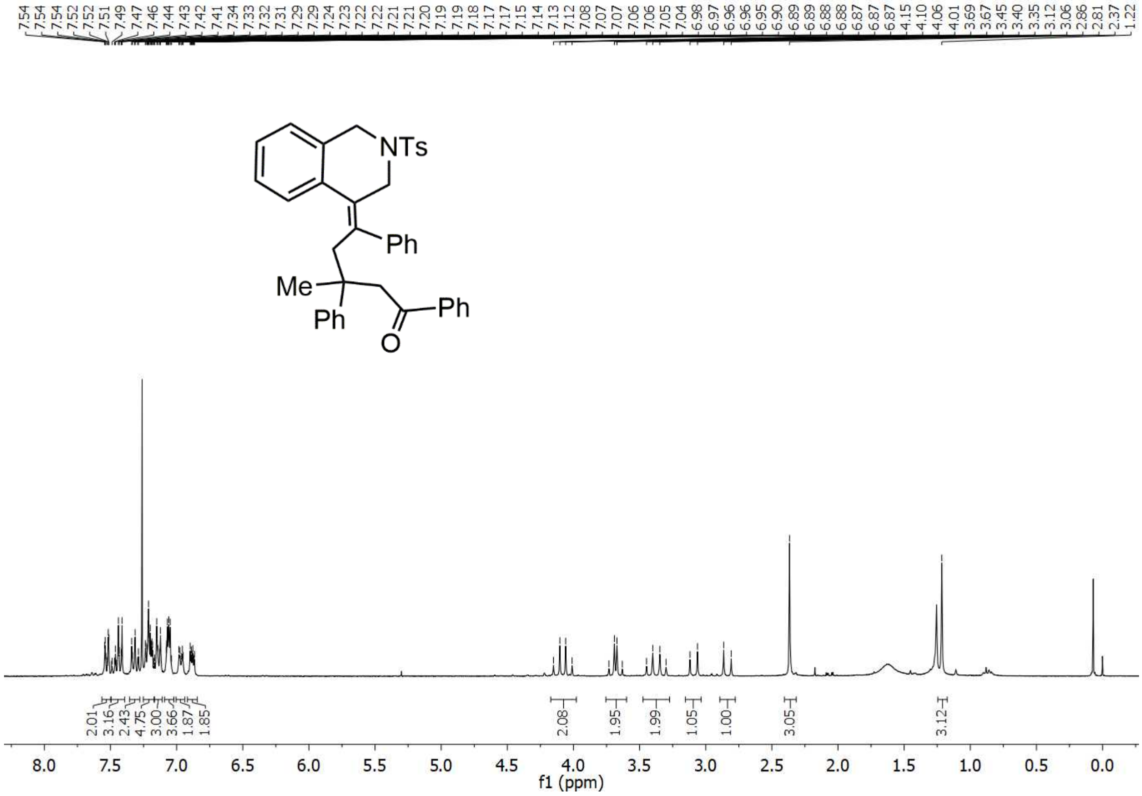

Figure S45. ^{1}H-NMR spectrum (300 MHz, $CDCl_3$) of compound **3r**.

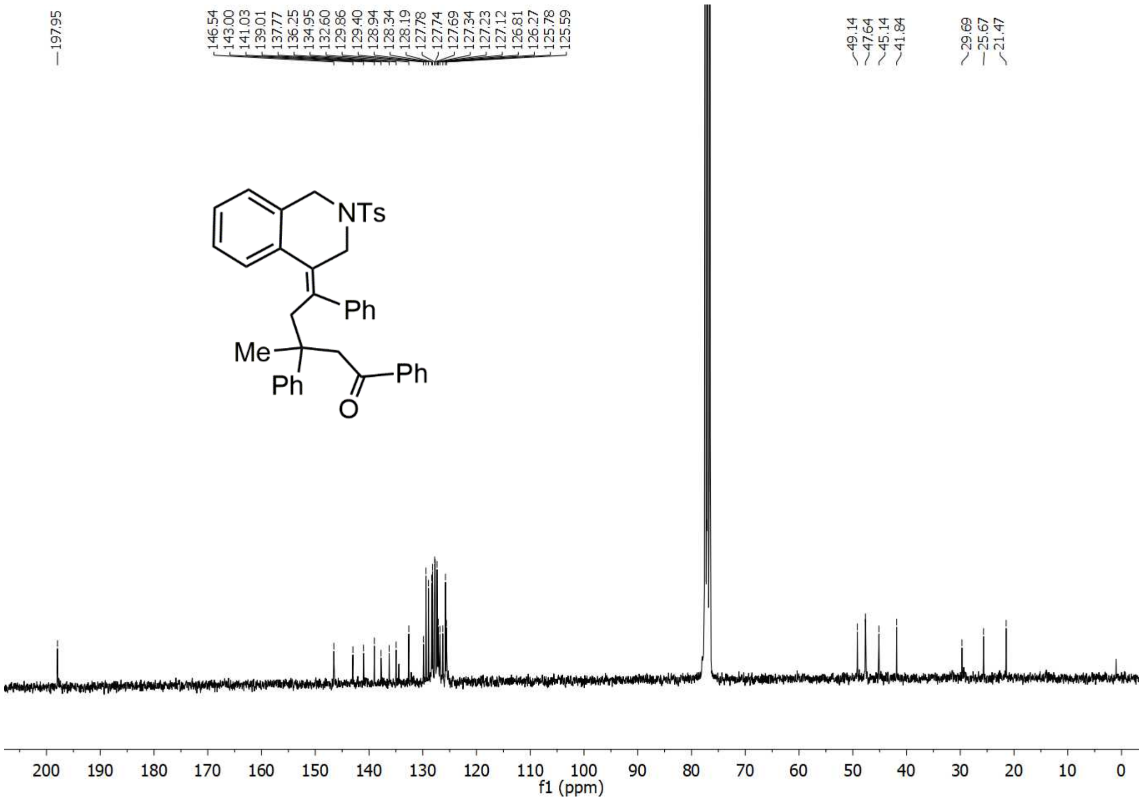

Figure S46. ^{13}C-NMR spectrum (75.45 MHz, $CDCl_3$) of compound **3r**.

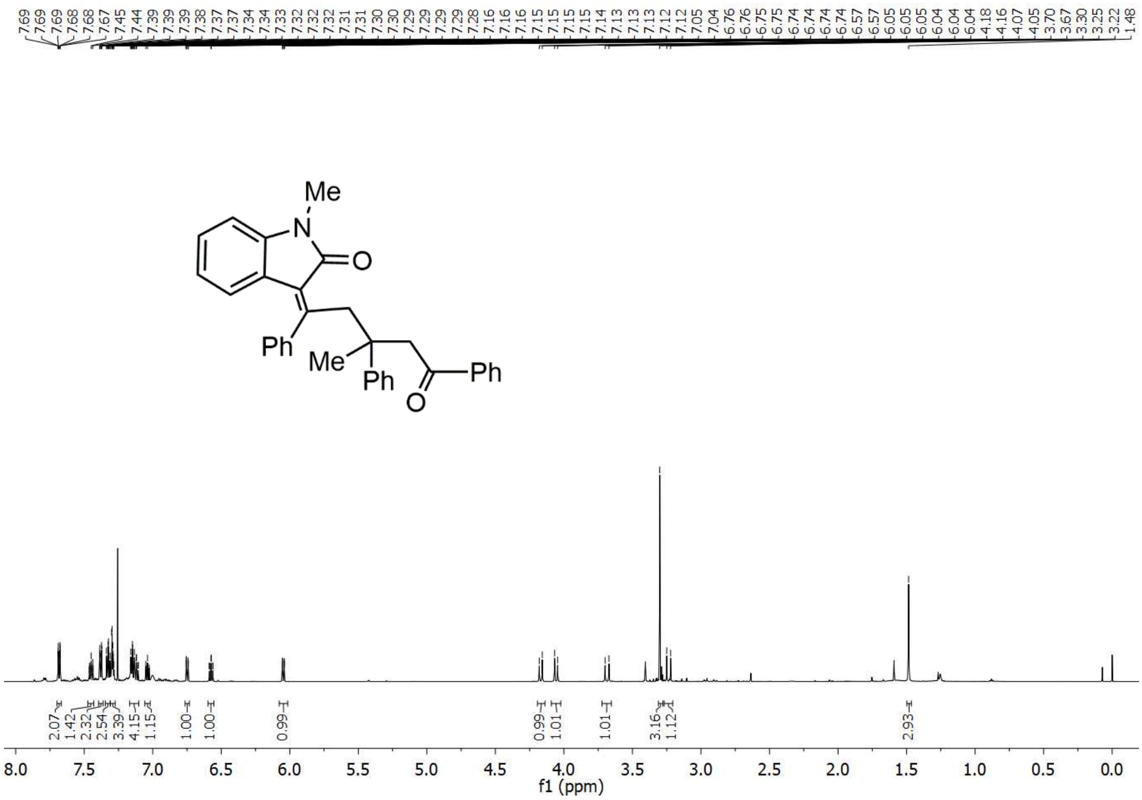

Figure S47. ^{1}H-NMR spectrum (600 MHz, $CDCl_3$) of compound **3s**.

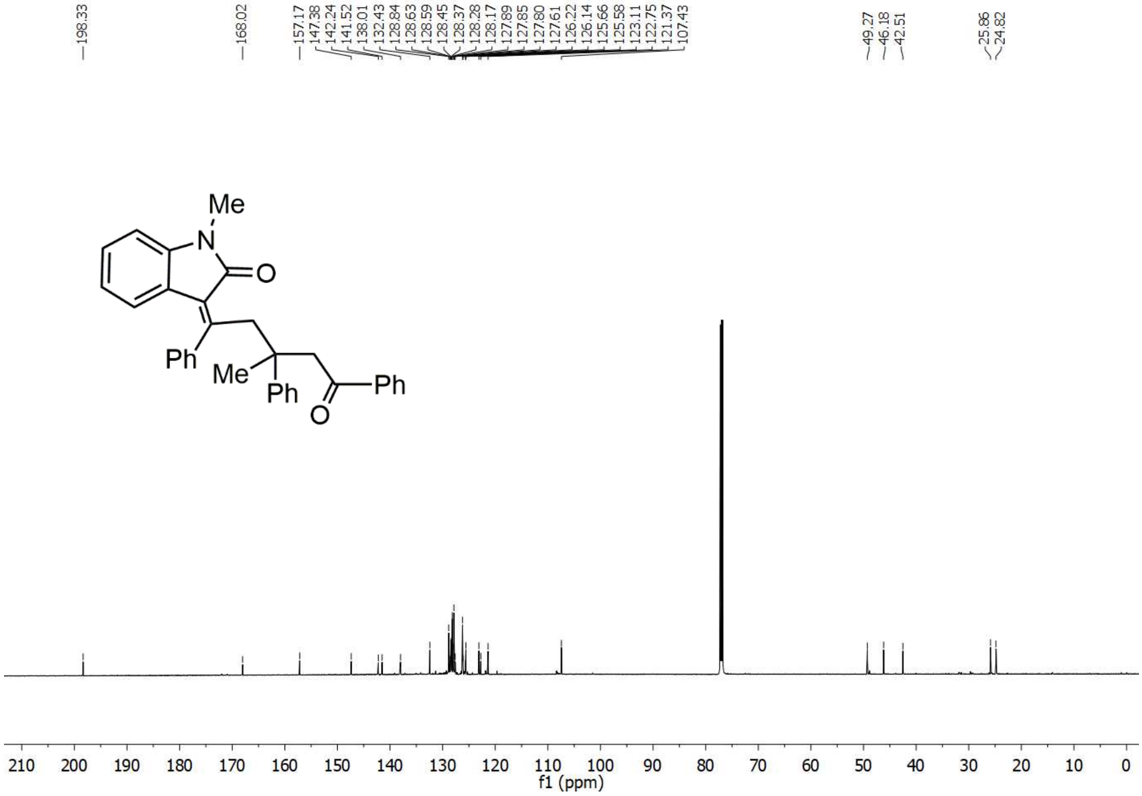

Figure S48. ^{13}C-NMR spectrum (151 MHz, $CDCl_3$) of compound **3s**.

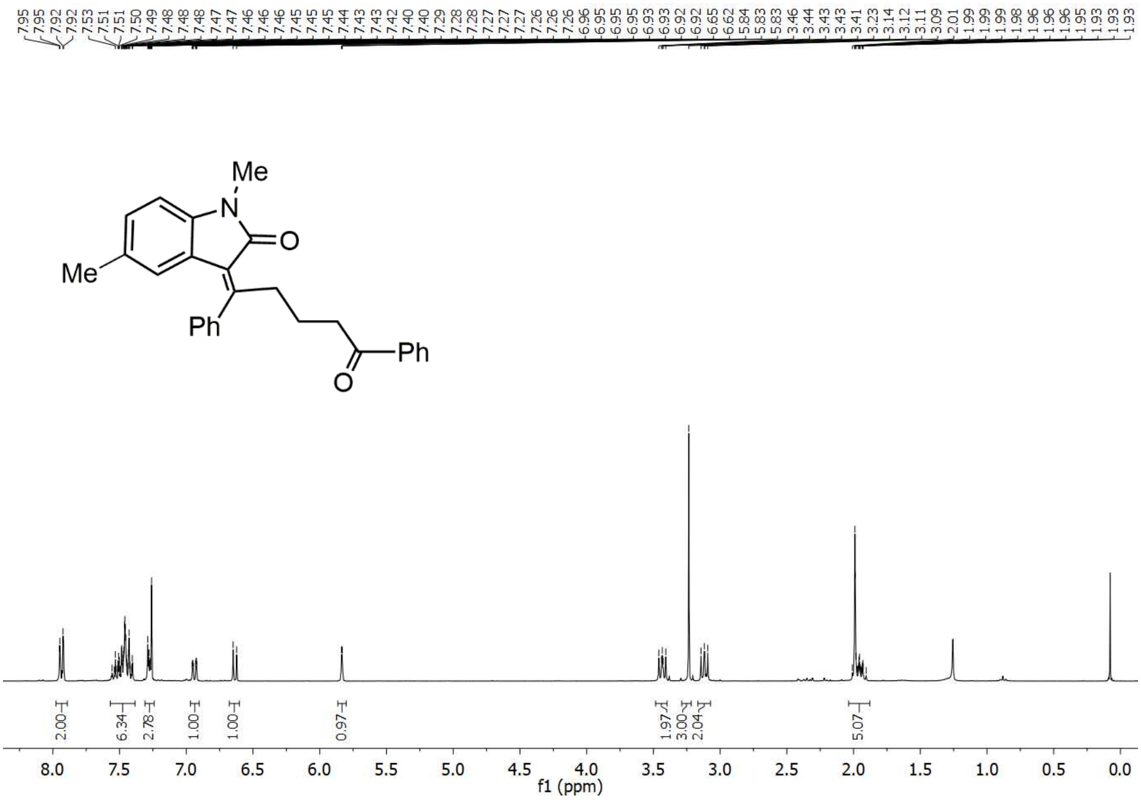

Figure S49. ^{1}H-NMR spectrum (300 MHz, $CDCl_3$) of compound **3t**.

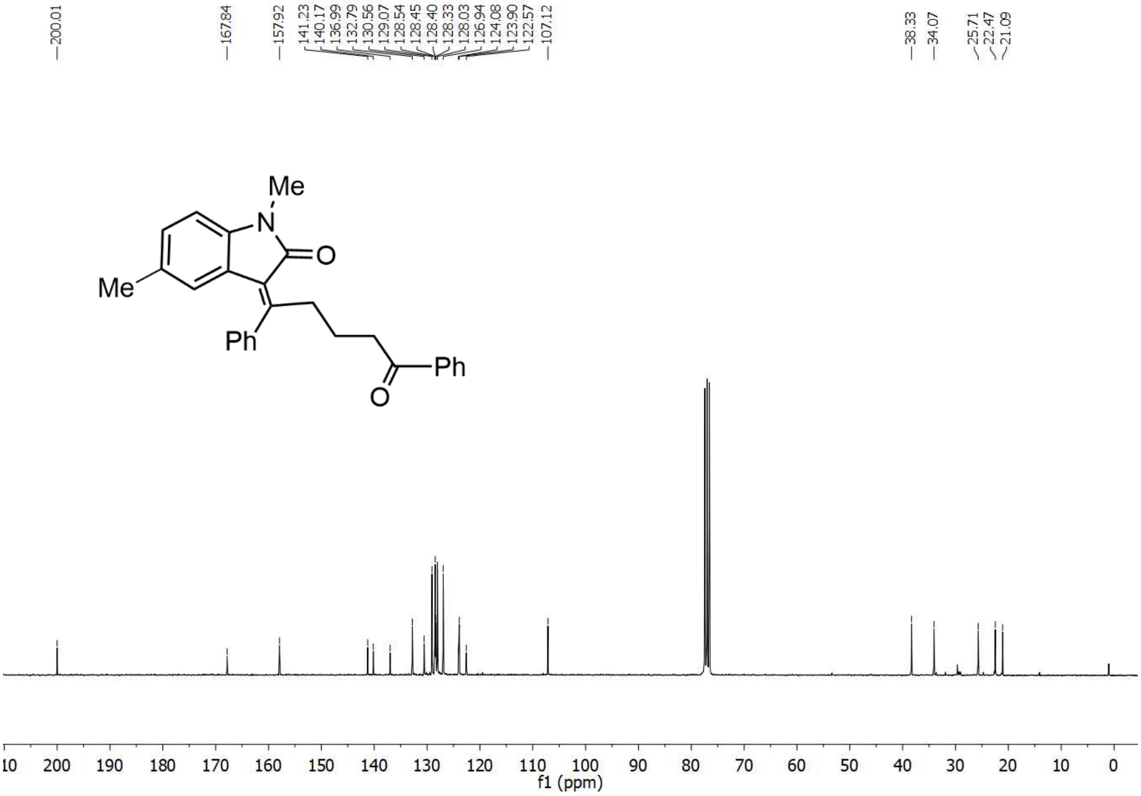

Figure S50. ^{13}C-NMR spectrum (75.45 MHz, $CDCl_3$) of compound **3t**.

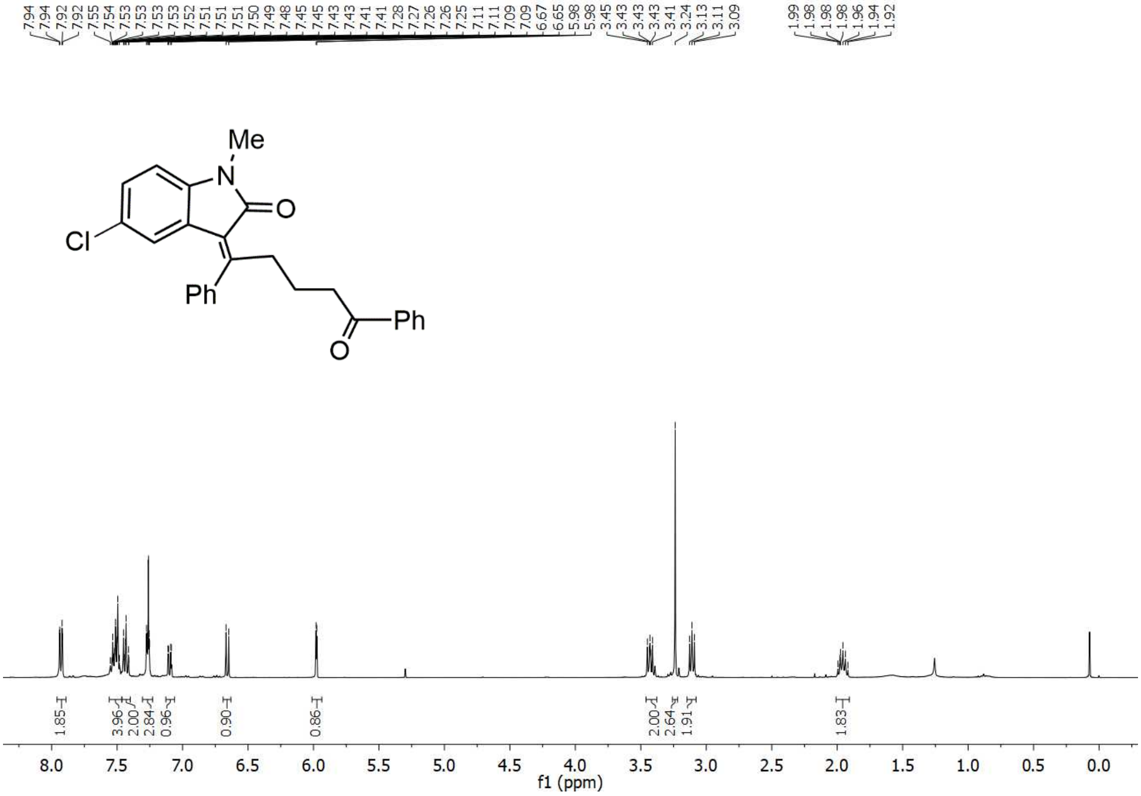

Figure S51. ^{13}C-NMR spectrum (300 MHz, $CDCl_3$) of compound **3u**.

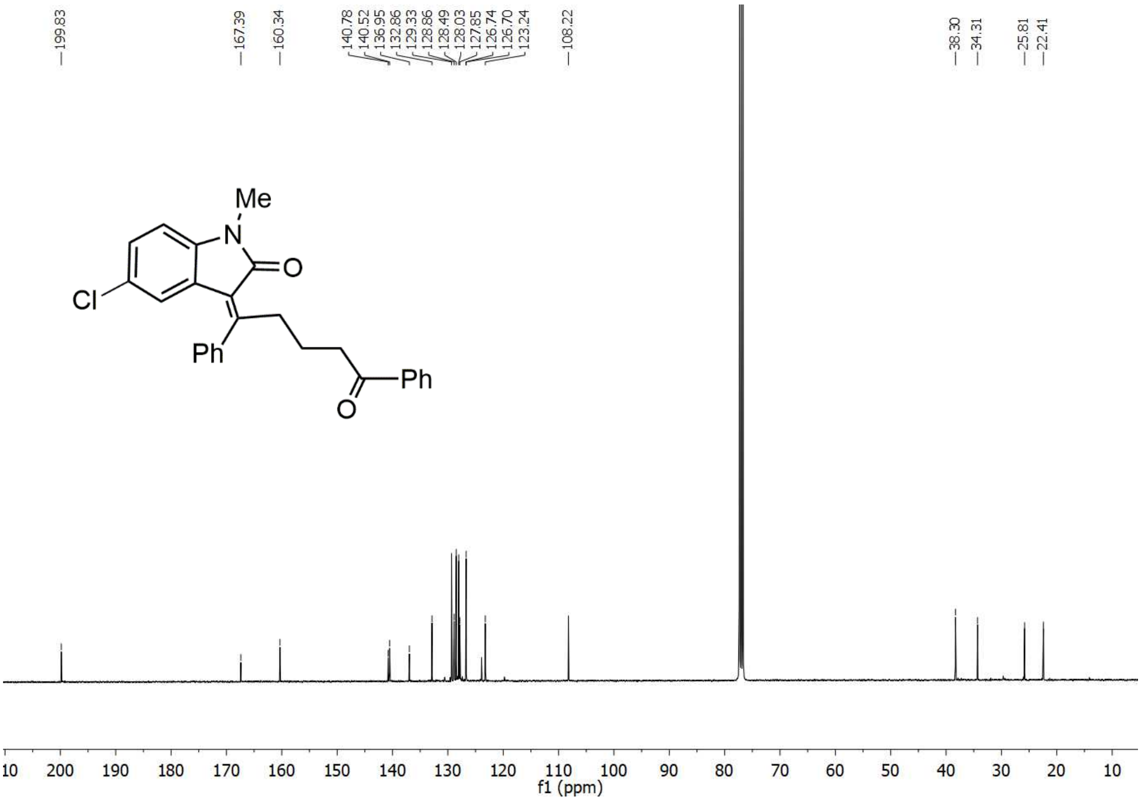

Figure S52. ^{13}C-NMR spectrum (100.1 MHz, $CDCl_3$) of compound **3u**.

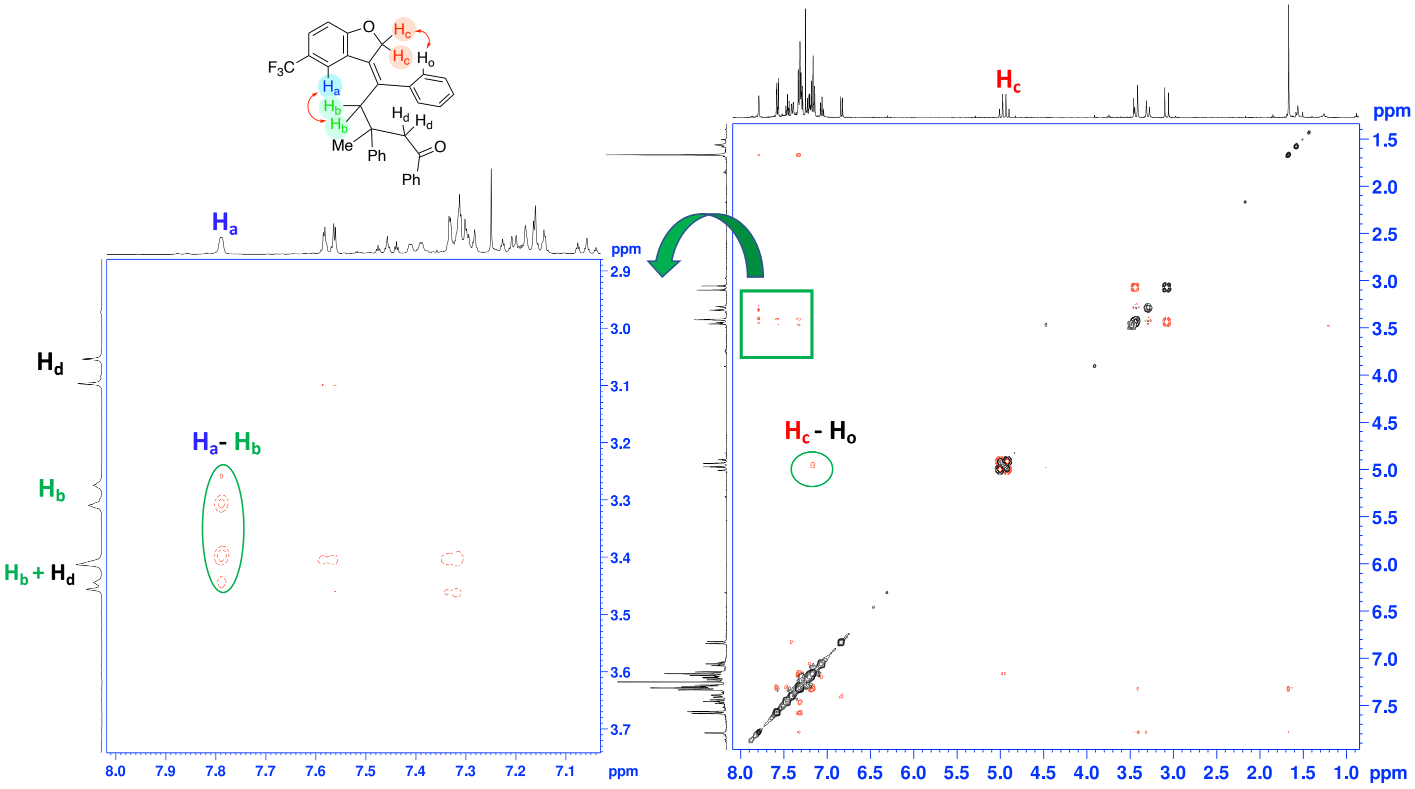

Figure S53. NOESY-NMR spectra (300 MHz, $CDCl_3$) of compound **3d**.

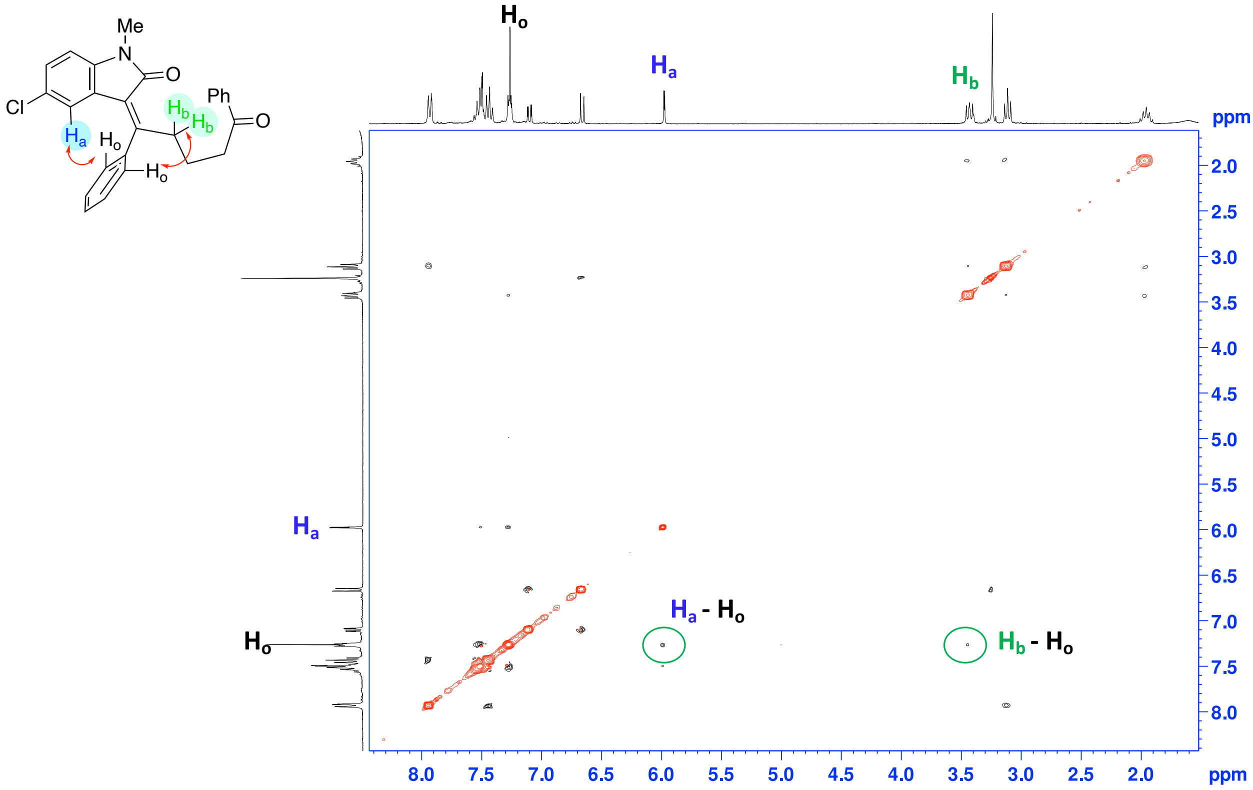

Figure S54. NOESY-NMR spectra (300 MHz, $CDCl_3$) of compound **3u**.

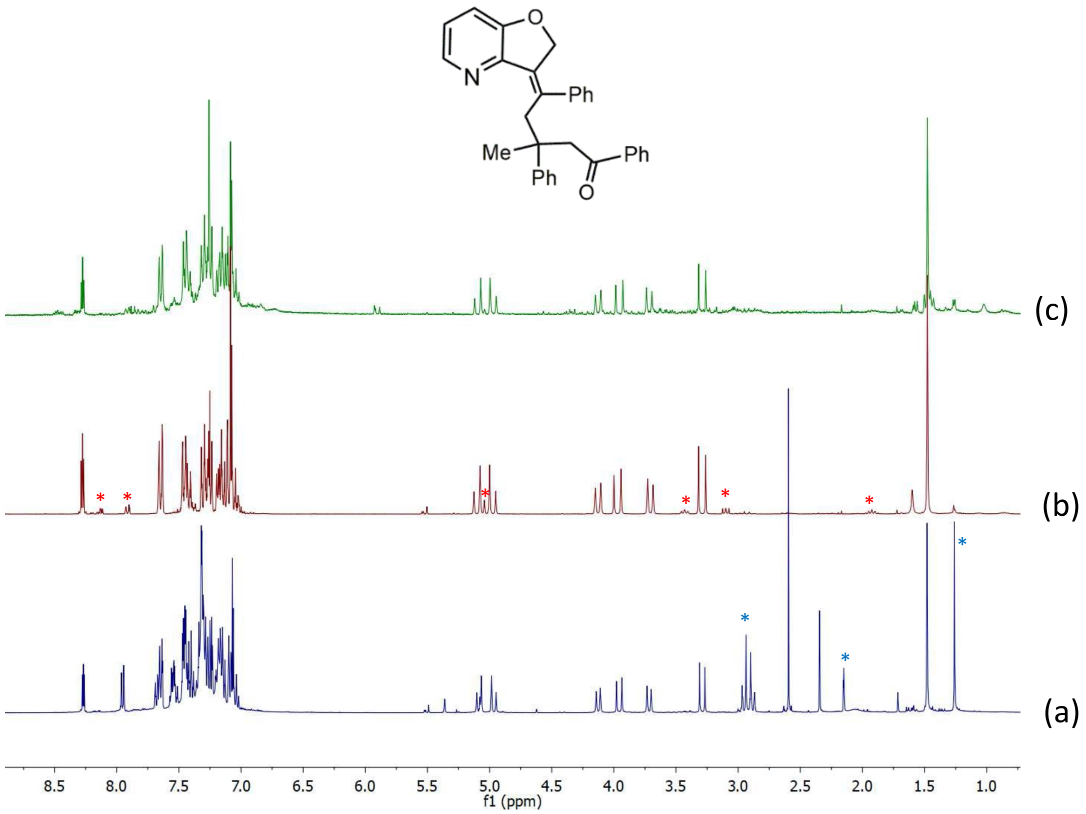

Figure S55. Evolution of ^{1}H-NMR of compound **3f**. (a): ^{1}H-NMR in $CDCl_3$ of the crude reaction mixture leading to compound **3f**. Asterisks in blue correspond to unreacted excess cyclobutanol reagent. (b): ^{1}H-NMR in $CDCl_3$ of the compound **3f** upon purification in silica gel. Asterisks in red correspond to new species not presente in the crude reaction mixture arising from partial decomposition of the compound **3f**. (c): ^{1}H-NMR of the purified compound **3f** upon five days in $CDCl_3$ solution at rt. It can be appreciated that a mixture of new and unidentified compounds appear in the reaction mixture.
